# Causal Relationship Between Circulating Metabolites and Sarcopenia‐Related Traits: A Mendelian Randomization and Experimental Study

**DOI:** 10.1002/fsn3.4624

**Published:** 2025-01-09

**Authors:** Weihui Qi, Xinning Mao, Zhenglin Mei, Li Zhu, Yinyan Shao, Guofen Ge, Gaoyong Jia, Hao Pan, Dong Wang

**Affiliations:** ^1^ Department of Orthopaedics Hangzhou Traditional Chinese Medicine Hospital Affiliated to Zhejiang Chinese Medical University Hangzhou China; ^2^ Department of Orthopaedics Hangzhou Dingqiao Hospital Hangzhou China; ^3^ Institute of Orthopaedics and Traumatology Hangzhou Traditional Chinese Medicine Hospital Affiliated to Zhejiang Chinese Medical University Hangzhou China

**Keywords:** glycine, insulin resistance, mendelian randomization, metabolite, SP

## Abstract

Sarcopenia (SP), an age‐associated condition marked by muscle weakness and loss has been strongly connected with metabolic factors according to substantial evidence. Nevertheless, the causal correlation between SP and serum metabolites, and the biological signaling pathways involved, is still not well understood. We performed a bidirectional two‐sample Mendelian randomization (MR) analysis to examine the causal relationships between 1091 levels and 309 ratios of metabolites with SP traits, alongside investigating the relevant biological signaling pathways. Additionally, we explored the differential expression of plasma metabolites and potential biological signaling pathways in an animal model of SP. When SP was utilized as the outcome, we identified 11 robust causal associations between seven metabolite levels/ratios and SP‐related traits using Bonferroni's correction (threshold: *p* < 0.05). We verified the stable causal association of glycine levels and SP in the validation. As for the reverse MR analysis, there were 11 strong causal relationships with 11 plasma metabolite levels/ratios remaining after multiple contrast correction. Additionally, biological signaling pathway analysis showed that glycine metabolism, insulin resistance, and cAMP signaling pathways may contribute to the connection between metabolites and SP. Mendelian validation of various datasets and observations in animal serum metabolomics suggests a strong association between glycine metabolism and SP. Our results indicate that the identified metabolites and biosignaling pathways could serve as important circulatory metabolic biomarkers for the screening and prevention of SP in clinical settings. Additionally, they represent potential molecules for future exploration of mechanisms and selection of drug targets.

## Graphical abstract

Sarcopenia, characterized by muscle atrophy and frailty, has a complex relationship with metabolic factors that remains poorly understood. Using bidirectional Mendelian randomization, we identified 11 robust causal associations between plasma metabolites, particularly glycine, and sarcopenia‐related traits, alongside significant biological signaling pathways like glycine metabolism and insulin resistance. Our findings highlight these metabolites and pathways as potential biomarkers for sarcopenia screening and targets for future therapeutic exploration.

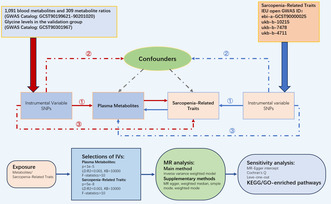

## 1 Introduction

Sarcopenia (SP), a widespread and progressive muscle disorder defined as rapid decline in muscle mass, strength and function with age, was introduced by Rosenberg in 1989 (Cruz‐Jentoft and Sayer 2019; Rosenberg 1997). The granting of an ICD‐10‐CM code to SP in 2016 allowed for its recognition as a disease entity, similar to the early acknowledgment of osteoporosis as a pathological condition (Anker, Morley, and von Haehling 2016; Roman, Mahoney, and Mohamadi 2013). This development has significant implications in enhancing our understanding and facilitating further research on SP. 10%–16% of the elderly population worldwide is affected by SP, and as the global population ages, the number of patients with SP will continue to increase, which will have adverse effects on human health and impose a huge burden on society and healthcare systems (Beard et al. 2016; Yuan and Larsson 2023). Various studies have demonstrated that SP can significantly impair the functional capacity for activities of daily living, lead to mobility disorders, elevate susceptibility to fractures and osteoporosis, and escalate mortality rates among elderly individuals (Kaplan et al. 2020; Malmstrom et al. 2016; Morley et al. 2011; Schaap et al. 2018). Moreover, it is closely associated with endocrine, cardiovascular, respiratory, and neuropsychiatric ailments (Endo et al. 2021; Izzo et al. 2021; Nagano et al. 2021; Ye et al. 2023). The European Working Group on SP in Older People (EWGSOP2) defines reduced strength of muscle as the key feature of SP, employs muscle mass measurement for its diagnosis, and recognizes impaired physical performance as a marker of severity (Cruz‐Jentoft et al. 2019). Despite an increasing number of studies on SP, the underlying pathophysiological mechanisms contributing to its onset and progression remain elusive.

Metabolites are substances produced or consumed during metabolic processes and serve not only as drivers of basic cellular physiological activities but also as functional small molecules that influence the onset and progression of diseases (Johnson, Ivanisevic, and Siuzdak 2016; Schlosser et al. 2023). Liu et al. demonstrated that stearic acid is important in the metabolism of reduced skeletal muscle mass (Liu et al. 2022). Other metabolites, such as docosahexaenoic acid ethanolamine, tryptophan, gluconic acid, L‐alanine, and proline, are also considered potential circulating biomarkers for pathological processes in SP (Kim et al. 2023; Shin, Won, and Kim 2022). The findings of these studies indicate a strong correlation between SP and serum metabolites as well as metabolic processes.

Randomized controlled trials (RCTs) have significant reference value in the investigation of pathogenic factors and disease risks; however, their extensive implementation has been hindered by various clinical constraints. MR methodology uses single‐nucleotide polymorphisms (SNPs) as instrumental variables (IVs) to explore causal relationships between biological factors and diseases (Burgess et al. 2019; Smith and Ebrahim 2003). By excluding the influence of confounder on outcomes, this approach enhances the strength of evidence for causal inference, which is similar to an RCT (Davey Smith and Hemani 2014; Sobczyk et al. 2023). Depending on the continuous reporting and updating of large‐scale genome‐wide association study (GWAS) data sources, large‐sample MR can be performed. Recently, numerous MR researches utilizing GWAS data have been published to investigate disease‐associated metabolites, gut microbiota, immune cells, and inflammatory factors (Bouras et al. 2022; Chen et al. 2023; He et al. 2022; Long et al. 2023).

In this study, we utilized valid genetic variants extracted from GWAS summary data of 1400 blood metabolite levels/ratios published by Chen et al. (2023) in the discover cohort and glycine levels by Karjalainen et al. (2024) in the validation cohort to investigate their association with the traits related to SP, and the direction of causality was conducted by reversing exposure and subsequent outcomes. After that, an untargeted serum metabolomics analysis in mice was performed to observe the alterations of serum metabolites in a SP model and investigate the potential biological pathways of metabolism and SP combined with the above result of MR. The present study employed a combination of two widely used research methodologies to exploring the metabolites and related biological pathways with SP, thereby offering novel insights for the treatment and prevention of patients affected by SP.

## 2 Method

### 2.1 Overview of the Study

**FIGURE 1:**
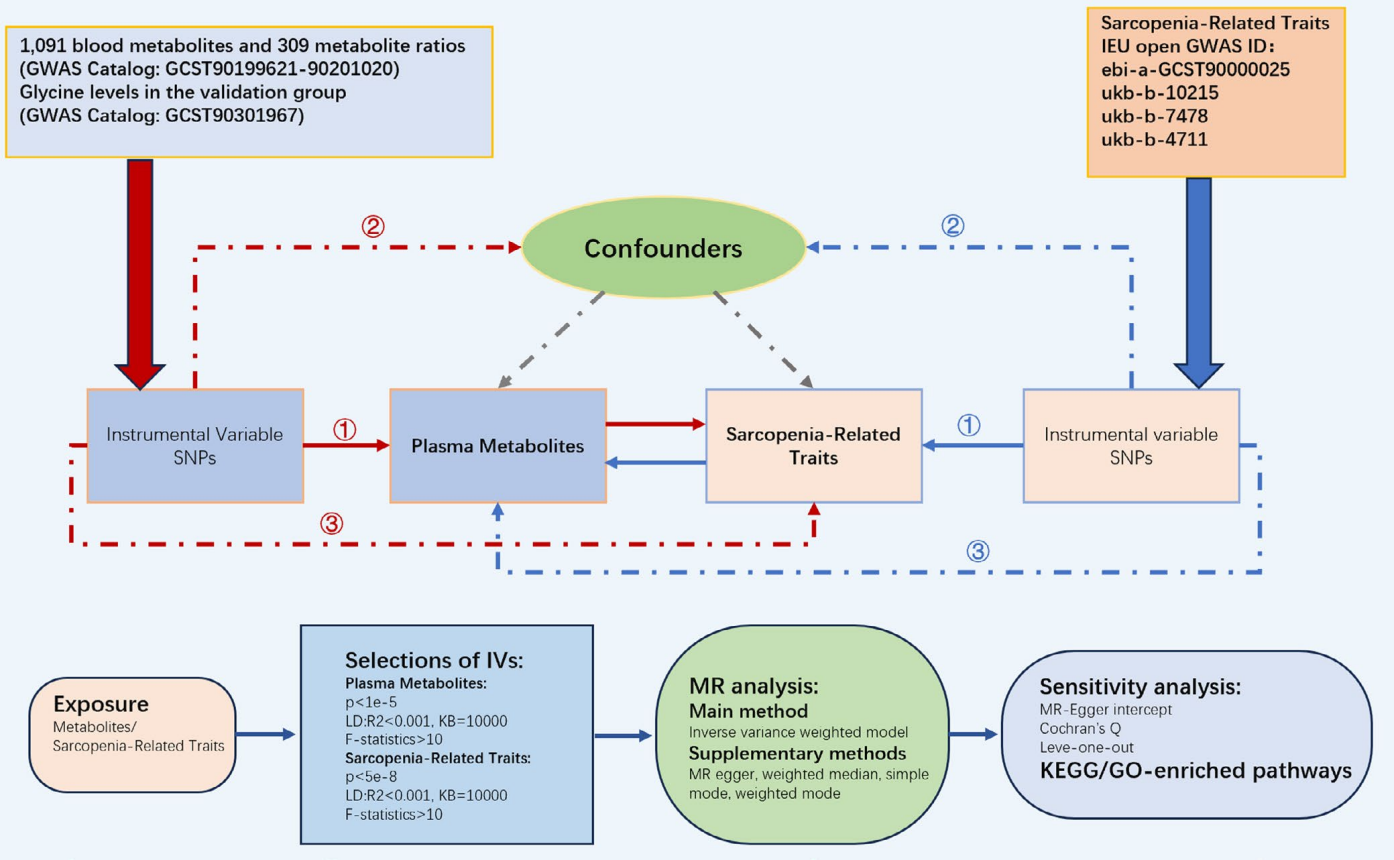
The study design overview of the MR study. Assumption I: The instrumental variables are significantly correlated with exposure; Assumption II: The genetic instruments are not subject to confounder that may affect the association between exposure and outcome; Assumption III: The genetic instruments affect the outcome only through the exposure (Appendicular lean mass: Ebi‐a‐GCST90000025; Hand grip strength [right]: Ukb‐b‐10,215; Hand grip strength [left]: Ukb‐b‐7478; Usual walking pace: Ukb‐b‐4711).

We carried out a bidirectional two‐sample MR analysis to evaluate the influence of plasma metabolites and SP‐related traits, using previously published GWAS summary statistics. The IVs employed in the MR analysis met the key assumptions as follows (Emdin, Khera, and Kathiresan 2017): (1) the IVs are strongly associated with the exposure; (2) the genetic instruments are not influenced by confounders affecting the exposure–outcome relationship; and (3) the genetic instruments impact the outcome solely through their effect on the exposure. We integrated IVs related to blood metabolites and SP traits and identified the genes most strongly associated with SNPs. The overview of the MR study is illustrated in Figure 1. Finally, we performed plasma metabolomic analysis in an animal model of SP and integrated it with MR results to investigate differential metabolites and biological pathways. The R 4.3.2 and R 4.3.3 software and R packages “foreach”, “TwoSampleMR”, and “ggplot2” were used in this study.

### 2.2 Data Source

The metabolites GWAS data in the discovery cohort were obtained from the most recent research by Chen et al., which involved approximately 15.4 million SNPs associated with 1400 levels/ratios of metabolites from 8299 individuals in the CLSA cohort, using UPLC‐MS/MS technology. The levels of glycine in the validation cohort were quantified in 27 European ancestry cohorts as published by Karjalainen et al. using nuclear magnetic resonance spectroscopy. The complete GWAS data are accessible via the GWAS Catalog server (GCST90199621‐90201020 and GCST90301967). GWAS summary statistics of the SP traits from the UK biobank were downloaded from the IEU open GWAS project. Appendicular lean mass (ALM), right hand grip strength (RGS), left hand grip strength (LGS), and usual walking pace (WP) were included as SP‐related traits as defined by EWGSOP2. The GWAS ID of ALM was ebi‐a‐GCST90000025, including 450,243 samples, yielding a total of 18,071,518 SNPs (Pei et al. 2020). The GWAS IDs of RGS, LGS, and WP were ukb‐b‐10,215, ukb‐b‐7478, and ukb‐b‐4711 including 461,089 cases and 9,851,867 SNPs, respectively. The study population exclusively comprised individuals of European descent, thereby mitigating any potential bias related to racial admixtures.

### 2.3 Selection of IVs

SNPs strongly associated with plasma metabolites were selected as potential IVs, and in order to retain a sufficient number of IVS, the threshold was *p* value < 1 × 10^−5^. To mitigate linkage disequilibrium (LD), we set the parameters to *r* ^2^ < 0.001 and a physical distance of 10,000 kb. SNPs with *r* ^2^ values greater than 0.001 within 10,000 kb of the most significant SNP were excluded. Subsequently, we obtained 1352 metabolites levels/ratios for further analysis. When SP‐related traits were used as exposure, the threshold was set at P‐value < 5 × 10^−8^, and other parameters were consistent as before. Then we deleted SNPs with missing data and palindromic SNPs with intermediate allele frequencies. Additionally, to assess the strength of IVs, we employed the following formula to calculate the *F*‐statistic for each SNP (*F* = *R* ^2^(*N* − 2)/(1 − *R* ^2^); *R* ^2^ = (2 × EAF × (1 − EAF) × *β* ^2^)/[(2 × EAF(1 − EAF) × *β* ^2^) + (2 × EAF × (1 − EAF) × *N* × SE^2^)]; *N*=Sample size; EAF = Effect allele frequency; *β* = genetic effect on the outcome; SE = Standard error of the genetic effect) (Burgess and Thompson 2011). An *F*‐statistic below 10 indicates weak IVs, which would be excluded from further MR analysis to ensure reliability.

### 2.4 Bidirectional Two‐Sample Mendelian Randomization

Initially, the method of random effects inverse variance weighted (IVW) was applied, using the Wald ratio to estimate the effect of exposure for each SNP, followed by a weighted linear regression analysis (Hemani et al. 2018). A *P*‐value from the IVW method (*P*_IVW_ < 0.05) was regarded as a potential causal relationship, thereby facilitating screening for metabolites potentially linked to SP. To minimize the risk of false‐positive findings, Bonferroni correction tests were used. In this study, 1400 metabolite levels/ratios were included, thus the adjusted *P* value was calculated as *P*_Bonferroni_ = 1400 × *P*_IVW_ and *P*_Bonferroni_ < 0.05 was considered to be a strong significant causal association. Second, to ensure accuracy and robustness, weighted median, MR‐Egger, weighted mode, and simple mode methods were used as complementary methods (Bowden, Davey Smith, and Burgess 2015; Bowden et al. 2016). Cochran's *Q* statistical analysis was applied to the IVW and MR‐Egger methods, along with the leave‐one‐out method to evaluate heterogeneity and determine whether a single SNP influenced the association (Liao et al. 2022; Sun et al. 2023). The MR‐Egger regression intercept was utilized to detect potential horizontal pleiotropy (Bowden, Davey Smith, and Burgess 2015). Finally, Scatter plots, funnel plots, and forest plots were generated to visually present the MR results.

### 2.5 Exploration of Potential Biological Signaling Pathways

To explore the possible biological signaling pathways between metabolites and SP, we extracted the SNPs in each strongly correlated causal association with a threshold of *p* < 0.05. After eliminating duplicated SNPs, we utilized Open Targets Genetics to integrate functional genomic data and quantitative trait loci from multiple sources. This allowed us to calculate the overall variant‐to‐gene (V2G) score. We then selected and annotated the highest overall V2G scores genes for further analysis.

### 2.6 Metabolomics Analysis in the SP Mice Model

Male C57BL/six mice (SPF, 8 weeks) were used in this experiment following the standard guidelines approved by our institution's Animal Ethics Committee. The mice were housed under conditions at a stable temperature of 24°C ± 2°C. After a 1‐week acclimation period, 12 mice were randomly assigned to two groups (*n* = 6 per group). To induce SP in the model group, D‐galactose was administered at a dosage of 500 mg/kg/day for 6 weeks, whereas saline (equivalent volume) was injected into the control group at the same site. After the treatment period, the mice were sacrificed following blood collection, and the gastrocnemius muscles were harvested. The tissue sections were processed using standard protocols and stained with hematoxylin and eosin (H&E) (*n* = 6). The histological alterations were examined using an optical microscope set at 40× magnification. The original UPLC‐QE‐MS datasets were obtained using a Thermo Q Exactive HFX high‐resolution mass spectrometry system. The raw data were then processed and analyzed with the Progenesis QI metabolomics software (Waters Corporation, Milford, USA) (*n* = 6). Principal component analysis (PCA) was used for comprehensive data evaluation to identify aberrant samples and assess quality control repeatability. The PCA score plot visually represents the dissimilarity between different observed samples, enabling the discrimination of sample similarity or dissimilarity. Additionally, univariate analysis effectively demonstrated the statistical significance of the metabolite alterations between the two samples and facilitated the identification of potential marker metabolites. Potential biomarker metabolites were screened based on a *P*‐value < 0.05 and a log2 fold change (lg2FC) > 1, followed by the generation of a volcano plot. Subsequently, differential metabolites were calculated and filtered, and pathway annotation was performed using the KEGG database. Finally, metabolites were categorized and enriched based on their involvement in specific pathways or functional roles.

## 3 Result

### 3.1 Primary Analysis of Plasma Metabolites and Traits Related to SP

**FIGURE 2:**
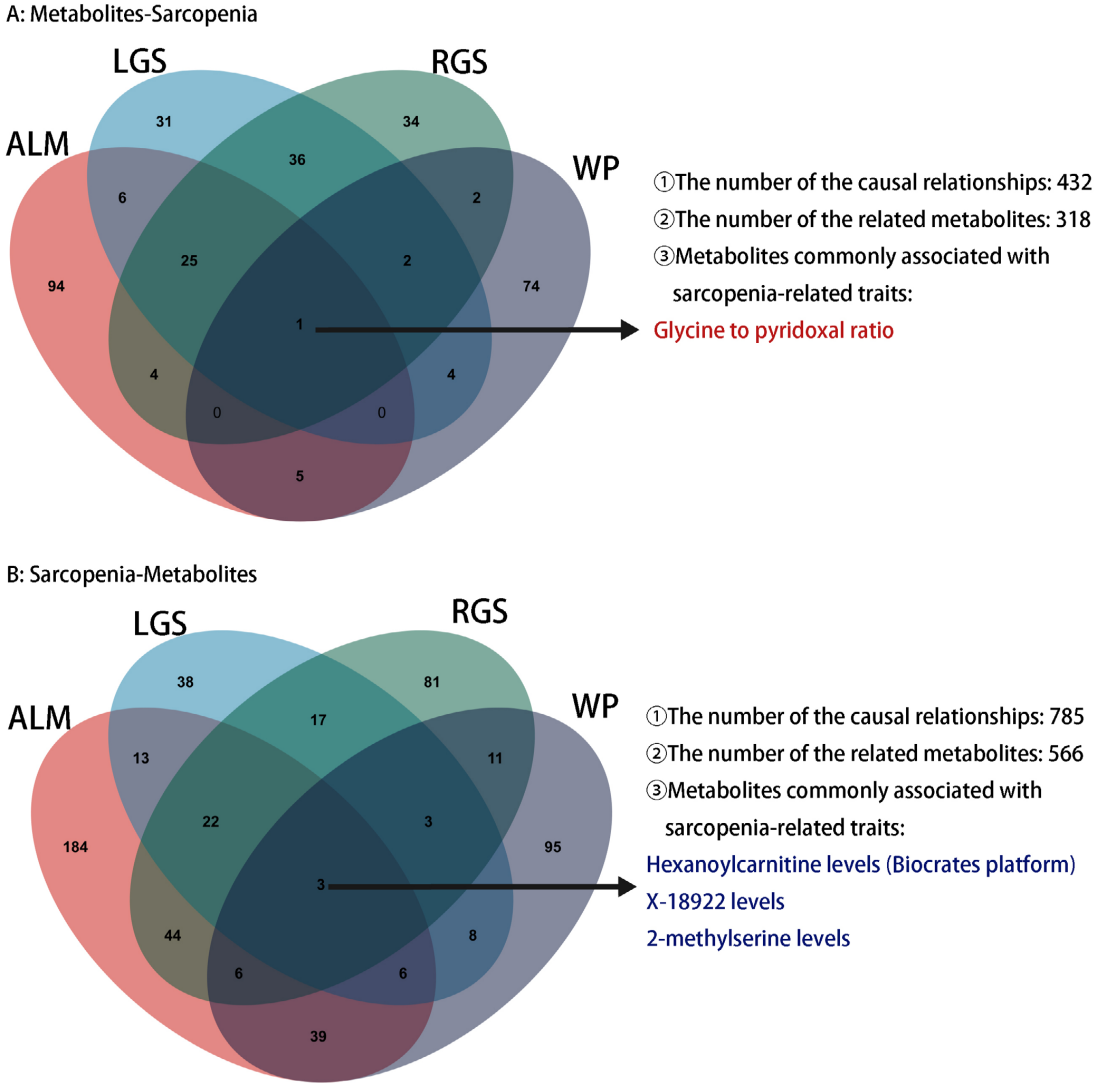
(A) Plasma metabolites screened by the IVW method with *p* < 0.05 as the threshold shown in the Venn diagram when the SP‐related traits are the outcome. (B) Plasma metabolites screened by the IVW method with *p* < 0.05 as the threshold shown in the Venn diagram when the SP‐related traits are the exposure.

The primary MR analysis was performed to identify potential causal relationships between plasma metabolites and SP‐related traits, applying a significance threshold of *P*_IVW_ < 0.05. The analysis revealed 432 causal associations involving 318 different plasma metabolite levels and ratios when considering SP as the outcome. Of the 318 levels/ratios of metabolites, we identified 135, 105, 104, and 88 plasma metabolites that were potentially associated with ALM, LGS, RGS, and WP (Table S1). In a Venn diagram (Bardou et al. 2014), we identified a potential causal relationship between the glycine‐to‐pyridoxal ratio and all of the traits related to SP in this research (Figure 2A). In total, 566 metabolite levels/ratios and 785 causal associations were identified when plasma metabolites were used as outcomes, including 317 for ALM, 110 for LGS, 187 for GRS, and 171 for WP (Table S2). In addition, hexanoylcarnitine, X‐18922, and 2‐methylserine levels are indicated in the Venn diagram as the intersection of four SP‐related traits (Figure 2B). The difference in metabolites associated with LGS and RGS may be due to differences in the intensity of daily activities between hands caused by different dominant hands.

**FIGURE 3:**
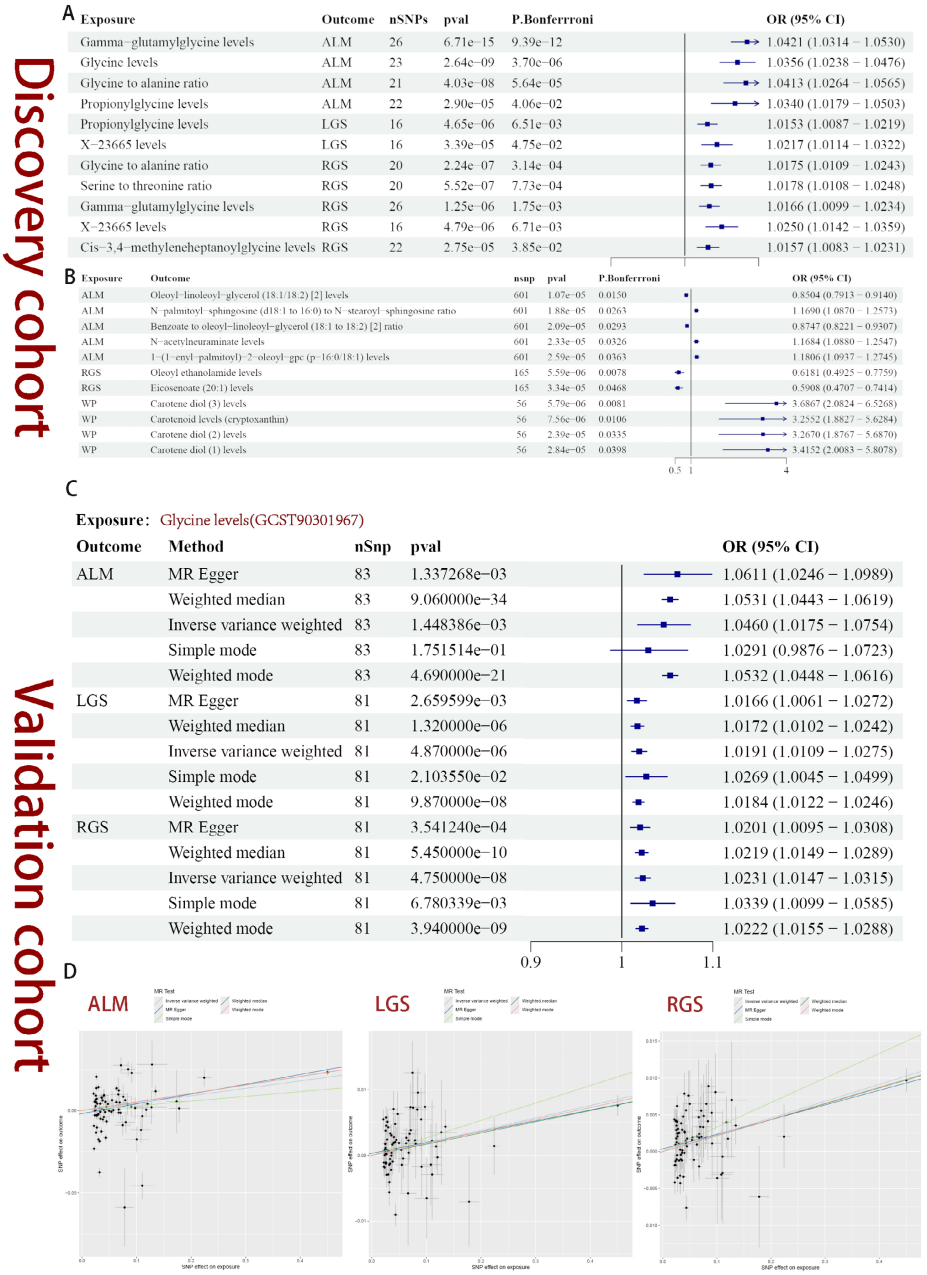
(A) Robust causal association tested by Bonferroni's correction when the SP‐related traits are the outcome for discovery cohort. (B) Robust causal association tested by Bonferroni's correction when the SP‐related traits are the exposure for discovery cohort. (C) Validation cohort results of glycine and SP‐related traits. (D) Scatter plots for the validation between blood glycine levels and SP‐related traits.

The Bonferroni correction enhanced the precision of the examination and mitigated the incidence of false positives. When SP was utilized as the outcome, we identified 11 robust causal associations between seven metabolite levels/ratios and SP‐related traits with a significance threshold of *P*_Bonferroni_ < 0.05(Gamma‐glutamylglycine levels‐ALM: OR = 1.0421, *P*_Bonferroni_ = 9.39E‐12; glycine levels‐ALM: OR = 1.0356, *P*_Bonferroni_ = 3.70E‐06; glycine to alanine ratio‐ALM: OR = 1.0413, *P*_Bonferroni_ = 5.64E‐05; propionylglycine levels‐ALM: OR = 1.0340, *P*_Bonferroni_ = 0.0406; propionylglycine levels‐LGS: OR = 1.0152, *P*_Bonferroni_ = 0.0065; X‐23665 levels‐LGS: OR = 1.0217, *P*_Bonferroni_ = 0.0475; glycine to alanine ratio‐RGS: OR = 1.0175, *P*_Bonferroni_ = 3.13E‐04; serine to threonine ratio‐RGS: OR = 1.0178, *P*_Bonferroni_ = 7.73E‐04; gamma‐glutamylglycine levels‐RGS: OR = 1.0166, *P*_Bonferroni_ = 0.0017; X‐23665 levels‐RGS: OR = 1.0250, *P*_Bonferroni_ = 0.0067; Cis‐3,4‐methyleneheptanoylglycine levels‐RGS: OR = 1.0157, *P*_Bonferroni_ = 0.0386) (Figure 3A). Interestingly, our findings suggested that amino acid metabolism, particularly glycine metabolism, may confer a protective effect against SP.

As for the reverse MR analysis, there were 11 strong causal relationships with 11 plasma metabolite levels/ratios that remained after multiple contrast correction (ALM‐Oleoyl‐linoleoyl‐glycerol (18:1/18:2) [2] levels: OR = 0.8504, *P*_Bonferroni_ = 0.0150; ALM‐N‐palmitoyl‐sphingosine (d18:1 to 16:0) to N‐stearoyl‐sphingosine (d18:1 to 18:0) ratio: OR = 1.1690, *P*_Bonferroni_ = 0.0263; ALM‐Benzoate to oleoyl‐linoleoyl‐glycerol (18:1 to 18:2) [2] ratio: OR = 0.8747, *P*_Bonferroni_ = 0.0293; ALM‐N‐acetylneuraminate levels: OR = 1.1684, *P*_Bonferroni_ = 0.0326; ALM‐1‐(1‐enyl‐palmitoyl)‐2‐oleoyl‐gpc (p‐16:0/18:1) levels: OR = 1.1806, *P*_Bonferroni_ = 0.0363; RGS‐Oleoyl ethanolamide levels: OR = 0.6181, *P*_Bonferroni_ = 0.0078; RGS‐Eicosenoate (20:1) levels: OR = 0.5908, *P*_Bonferroni_ = 0.0468; WP‐Carotene diol [3] levels: OR = 3.6867, *P*_Bonferroni_ = 0.0081; WP‐Carotenoid levels (cryptoxanthin): OR = 3.2552, *P*_Bonferroni_ = 0.0106; WP‐Carotene diol [2] levels: OR = 3.2670, *P*_Bonferroni_ = 0.0335; WP‐Carotene diol [1] levels: OR = 3.4152, *P*_Bonferroni_ = 0.0398) (Figure 3B).

Based on the aforementioned findings, a significant causal association has been identified between various types and ratios of glycine modifications and the traits related to SP. To further validate the causal link between blood glycine levels and SP, we conducted a validation using GWAS data from other sources. The results confirmed a strong causal association between glycine and ALM (OR_MR‐Egger_ = 1.0611, *P*_MR‐Egger_ = 0.0013; OR_Weighted‐median_ = 1.0531, *P*_Weighted‐median_ = 9.06E‐34; OR_IVW_ = 1.0460, *P*_IVW_ = 0.0014; OR_Simple mode_ = 1.0291, *P*_Simple mode_ = 0.1752; OR_Weighted mode_ = 1.0532, *P*_Weighted mode_ = 4.69E‐21), LGS (OR_MR‐Egger_ = 1.0166, *P*_MR‐Egger_ = 0.0027; OR_Weighted‐median_ = 1.0531, *P*_Weighted‐median_ = 1.32E‐06; OR_IVW_ = 1.0191, *P*_IVW_ = 4.87E‐06; OR_Simple mode_ = 1.0172, *P*_Simple mode_ = 0.0210; OR_Weighted mode_ = 1.0184, *P*_Weighted mode_ = 9.87E‐08), and RGS (OR_MR‐Egger_ = 1.0201, *P*_MR‐Egger_ = 0.0004; OR_Weighted‐median_ = 1.0219, *P*_Weighted‐median_ = 5.45E‐10; OR_IVW_ = 1.0231, *P*_IVW_ = 4.75E‐08; OR_Simple mode_ = 1.0339, *P*_Simple mode_ = 0.0068; OR_Weighted mode_ = 1.0222, *P*_Weighted mode_ = 3.94E‐09) when the SP‐related traits are the outcomes. The findings indicated that glycine, as a protective factor, was significantly associated with those three traits of SP. The consistency observed across the five different MR analysis methods confirms the robustness of the findings. These results were visually presented through forest plots (Figure 3C) and scatter plots (Figure 3D).

### 3.2 Sensitivity Analysis

The study employed five methods: IVW, MR‐Egger, weighted median, simple model, and weighted model. The findings consistently demonstrated that these highly correlated causal associations exhibited the same directionality in terms of odds ratio (OR) values as shown in the scatter plot (Figure S1 and Table S3), thereby providing robust evidence for the results. The MR‐Egger intercept test revealed no evidence of horizontal pleiotropy in the associations between plasma metabolites and SP‐related traits, except for Cis‐3,4‐methyleneheptanoylglycine levels‐RGS (MR‐Egger intercept = −0.002723, *p* < 0.01) (Tables [Link], [Link], [Link]). Cochran's *Q* statistical analysis indicated the presence of heterogeneity, particularly in relation to ALM; thus, we employed random effects IVW in our methodology. In the MR study design, allowance was made for heterogeneity to exist, and all five methods yielded OR values that were consistent in direction, thereby indicating the robustness of the findings. Notably, the exclusion of any single SNP associated with plasma metabolites or SP‐related traits did not yield any significant alterations in the overall findings thorough leave‐one‐out analysis (Figure S2). The bidirectional MR design suggests that there is no significant reverse effect between strongly correlated causal associations in this study.

### 3.3 KEGG/GO Enriched Biological Signaling Pathways

**FIGURE 4:**
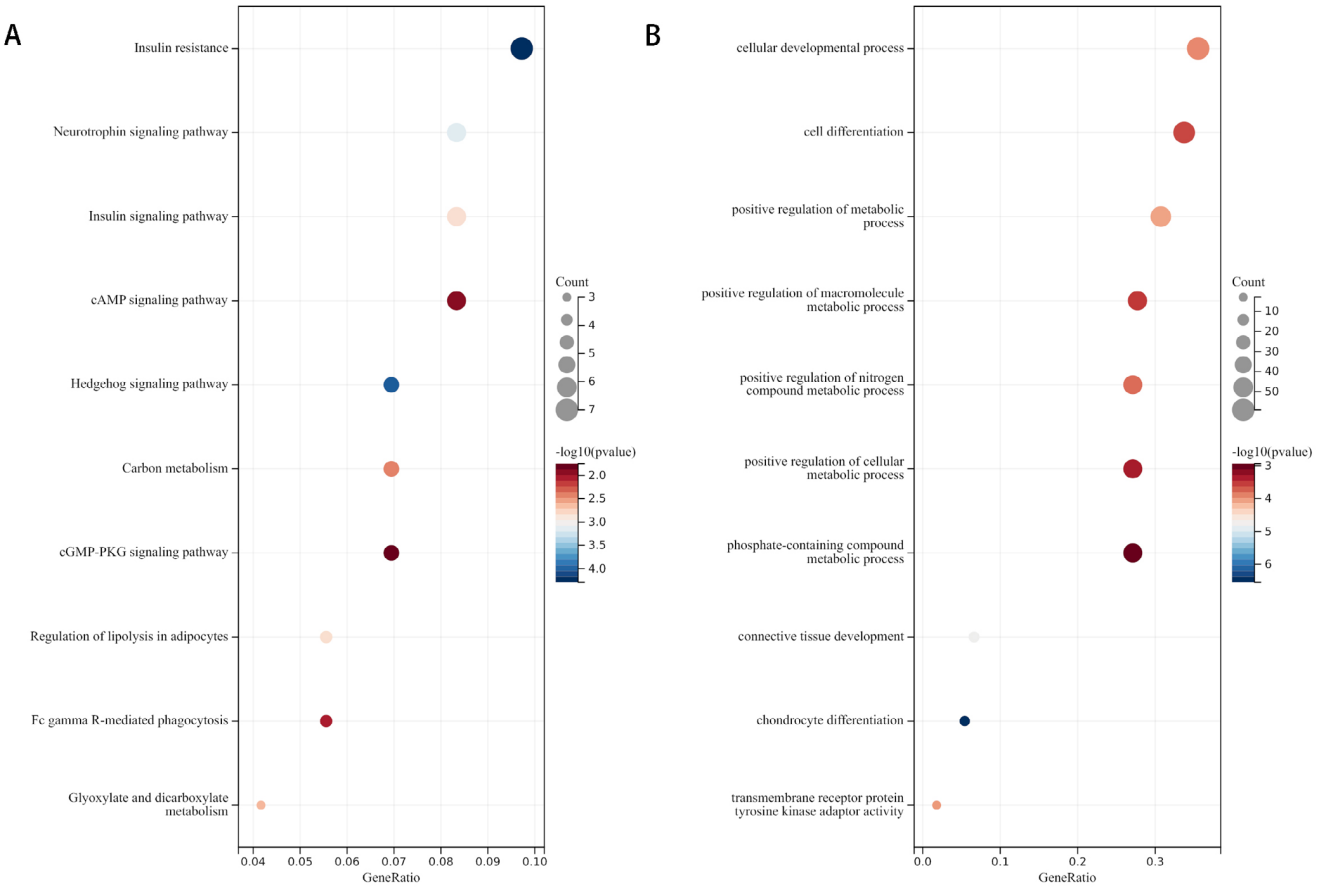
(A) The top 10 pathways for KEGG enrichment analysis. (B) The top 10 pathways for GO enrichment analysis.

We identified the key loci associated with blood metabolites and SP‐related traits and mapped these loci to a gene database for further analysis. After eliminating duplicates, we identified 193 genes corresponding to 200 independent SNPs with the highest overall V2G score as potential candidate genes, which may play a crucial role in the association between serum metabolites and SP (Table S7). The potential biological signaling pathways of these genes were further explored using KEGG and GO enrichment analyses, and the top 10 pathways were listed based on *P*‐values and gene ratios. KEGG enrichment analysis indicated that insulin resistance, insulin signaling pathway, neurotrophin signaling pathway, cGMP‐PKG signaling pathway, cAMP signaling pathway, and Fc gamma R‐mediated phagocytosis are pivotal signaling pathways (Figure 4A). GO enrichment analysis revealed the cellular development, differentiation, and positive regulation of metabolic processes. GO enrichment analysis suggested that strongly correlated genes may cause the association between metabolites and SP by affecting the process of cellular development, differentiation, and positive regulation of metabolic processes (Figure 4B).

### 3.4 Metabolomics Analysis in the SP Mice Model

**FIGURE 5:**
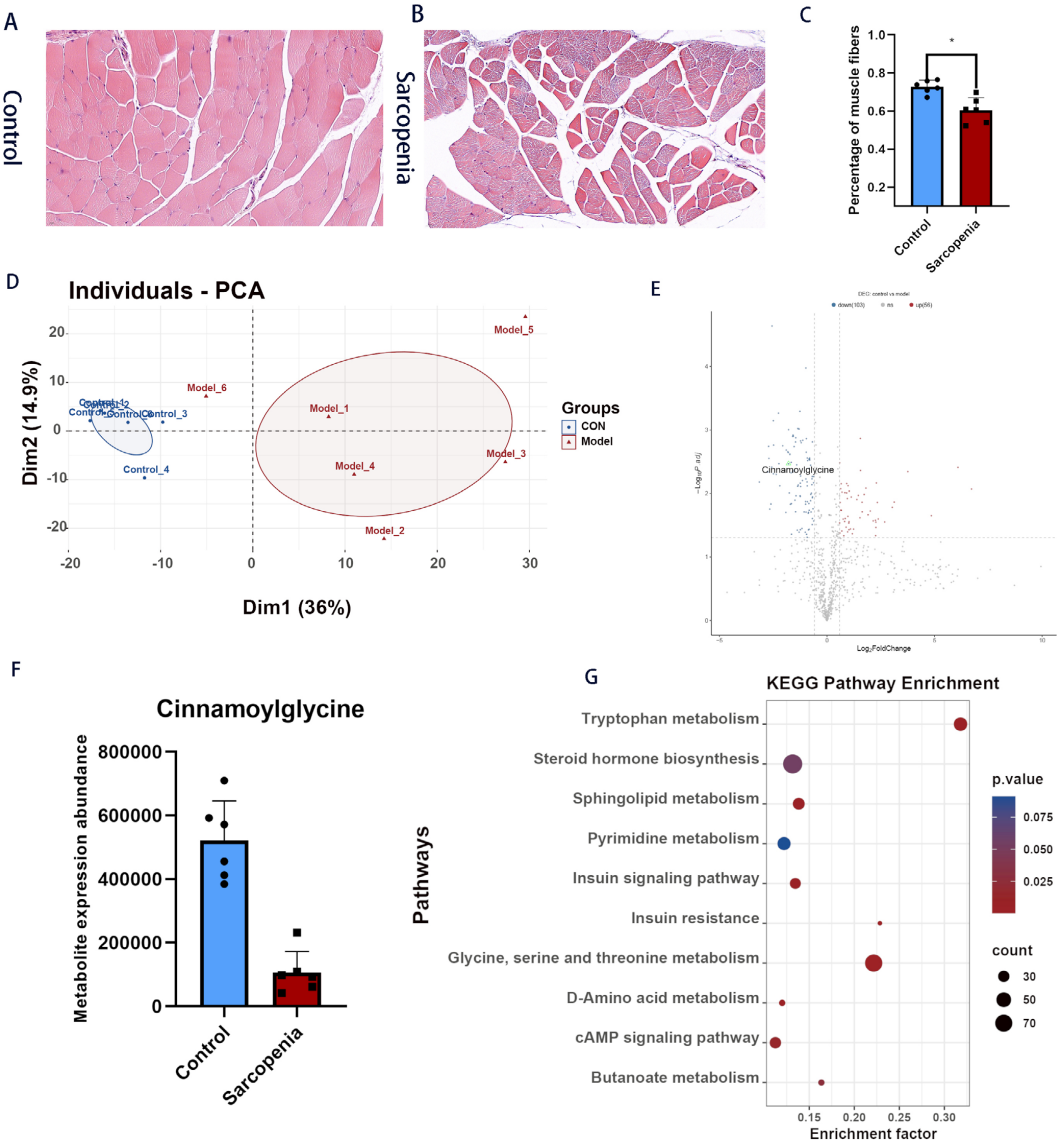
(A) H&E staining of the control group. (B) H&E staining of the model group. (C) Skeletal muscle fiber ratio (*p < 0.05). (D) PCA plot between the SP group and the model group. (E) The volcano plots of the differential metabolites between the control group and the model group. (F) Differential expression of cinnamoylglycine between the control group and the model group. (G) The top 10 pathways for KEGG enrichment analysis.

HE staining results revealed that, compared to the control group, the gastrocnemius muscle fibers in the model group were locally loose, with widened interstitial tissue. These changes indicate that the SP model was successfully established (Figure 5A–C). The score plot generated by the PCA demonstrated a clear separation in serum metabolites between the SP group and the control group (Figure 5D). The volcano plots generated from the FC analysis identified differentially expressed serum metabolites, highlighting those that were either upregulated or downregulated between the SP and control groups. Among these, cinnamoyl glycine was specifically noted, as it can partially and indirectly reflect glycine levels (Figure 5E,F). According to the KEGG pathway enrichment analysis, D‐amino acid metabolism, tryptophan metabolism, steroid hormone biosynthesis, sphingolipid metabolism, pyrimidine metabolism, insulin signaling pathway, insulin resistance, glycine, serine, threonine metabolism, cAMP signaling pathway, and butanoate metabolism were considered to be the most important pathways between the SP and control group (Figure 5G).

## 4 Discussion

Recently, SP has been recognized as a metabolic‐related musculoskeletal disorder (Tournadre et al. 2019), not only because of its potential coexistence with various metabolic disorders, but also because of the continuous identification of associated metabolites (Chen et al. 2023). Although previous studies have explored the relationship between metabolites and SP, our research encompasses the most up‐to‐date and comprehensive GWAS data of metabolites available (Chen et al. 2024; Qian et al. 2024; Sha et al. 2023). We identified seven metabolites that were robust and significantly correlated using plasma metabolites as exposure in the bidirectional two‐sample MR analysis. Additionally, we identified 11 metabolites that were robust and significant using reverse analysis. Moreover, based on the positive findings, we conducted a Mendelian randomization analysis to validate the association between serum glycine levels and SP. Subsequently, we further substantiated this discovery through metabolomics analysis in SP mice.

Interestingly, when plasma metabolites were used as exposures, we observed a significant correlation between glycine metabolism and SP. Ham et al. (2014) verified that glycine supplementation could alleviate the loss of mass and function in model of cancer cachexia mouse. Genton et al. (2021) reported in a randomized double‐blind crossover trial that, compared to branched chain amino acids, glycine improved the fat‐free body mass index in patients undergoing chronic hemodialysis. There are studies that have explored further mechanisms of this phenomenon, Norikura et al. (2023) demonstrated that glycine metabolizes into an alpha‐keto acid (glyoxylic acid), which effectively promotes myogenesis in C2C12 cells. Additionally, in the animal model of SP utilized in this study, KEGG pathway enrichment analysis indicated that glycine, serine, and threonine metabolism were the pertinent biological signaling pathways. In conclusion, the findings of this study align with the aforementioned observational, clinical, randomized controlled, in vitro, and in vivo studies, suggesting that targeting glycine metabolism may represent a promising therapeutic approach for SP.

Another intriguing finding of this study was that when plasma metabolites were used as an outcome, and walking speed was significantly associated with multiple carotenoid diols and carotenoids (OR = 3.26–3.69, *P*_Bonferroni_ < 0.05). Vitamins are indispensable nutrients for the human body and have been extensively investigated in relation to SP (Cereda et al. 2022; Kato et al. 2024; Nasimi et al. 2021). Carotenoids are pigments essential for protecting plants from photooxidation and are a vital part of the human body's antioxidant defense system (Stahl and Sies 2003). The study findings revealed that elevated carotenoid and alpha‐tocopherol levels were independently associated with increased strength indexes (Semba et al. 2003). Grip strength and walking speed are essential indicators of SP; increased carotenoid intake has been associated with improved grip strength and faster walking speed, serving as protective factors against SP (Sahni et al. 2021). Kochlik et al. (2023) found that levels of β‐cryptoxanthin and β‐carotene were significantly lower in frail patients compared to non‐frail individuals, highlighting muscle catabolism as a characteristic of frailty. In this study, we revealed a significant relationship between increased walking speed and higher carotenoid levels, further supporting previous findings.

According to KEGG analysis of genes associated with MR and mouse metabolomics, insulin resistance, glycine, serine, and threonine metabolism, and cAMP signaling pathway are potentially implicated in the biological signaling pathways linking plasma metabolites to SP. Older individuals with insulin resistance experience accelerated muscle mass loss compared to non‐insulin‐resistant individuals due to age‐related reduction in available insulin‐responsive tissue, thereby promoting the insulin resistance (Park et al. 2009; Reaven 1988). Compensatory hyperinsulinemia caused by insulin resistance leads to inadequate inhibition of glycogen generation, accelerated protein degradation, reduced protein synthesis, and increased myostatin content, ultimately resulting in skeletal muscle loss (Bonaldo and Sandri 2013; Son et al. 2017). Our research findings suggest that the association between SP and metabolites may be through the insulin resistance pathway. Regarding the cAMP signaling pathway, Svensson et al. (2020) demonstrated that p300 and cAMP response element‐binding protein are essential for regulating and maintaining contractile function and transcriptional balance in skeletal muscle. Furthermore, the exchange protein directly activated by cAMP has been shown to play a vital role in enhancing exercise capacity by regulating PGC‐1α and fatty acid metabolism in skeletal muscle (So et al. 2020). Overall, these findings underscore the critical role of insulin resistance and the cAMP signaling pathway in the metabolic regulation of SP, highlighting potential therapeutic targets for mitigating muscle mass loss in aging populations.

The current study had several notable strengths. Firstly, it contains the most comprehensive serum metabolite data and validates serum glycine levels in another dataset according to the positive finding. Second, we performed multiple rigorous sensitivity analyses and Bonferroni correction tests to confirm the robustness and reliability of the MR study results. Subsequently, gene enrichment analysis was performed using significantly correlated IVs to elucidate the potential biological signaling pathways linking plasma metabolites with SP. Lastly, a mouse model metabolomics study was undertaken to validate the differential expression of plasma metabolites and explore plausible biological signaling pathways between sarcopenic and normal mice.

However, this study had some limitations. First, due to the limited number of SNPs that achieved genome‐wide significance, we opted to relax the *P*‐value threshold, a practice commonly adopted in previous studies. However, the *F*‐statistic of each SNP is > 10, indicating no evidence of weak IV bias. Second, although we prevented confounding interference through MR analysis, the bias caused by confounding factors could not be completely excluded. Furthermore, the GWAS data were limited to individuals of European ancestry, and the results are limited to explaining the European population and require further study in other races. Finally, the objective of the MR analysis was to elucidate the causality. However, in pathway enrichment analysis and animal model metabolomics studies, subsequent investigations have been limited to correlation‐based approaches without delving into causal associations.

## 5 Conclusion

In summary, this study highlights a strong causal relationship between serum metabolites and SP through MR analysis and animal metabolomics research. Our findings suggest that glycine metabolism pathways may serve as potential therapeutic targets for SP, warranting further investigation as candidate targets in future studies.

## Author Contributions

**Weihui Qi:** data curation (equal), software (equal), validation (equal), writing – original draft (equal). **Xinning Mao:** validation (equal), visualization (equal), writing – original draft (equal). **Zhenglin Mei:** investigation (equal), methodology (equal), software (equal), writing – review and editing (equal). **Li Zhu:** data curation (equal), validation (equal). **Yinyan Shao:** supervision (equal), visualization (equal). **Guofen Ge:** data curation (equal), investigation (equal). **Gaoyong Jia:** methodology (equal), software (equal). **Hao Pan:** project administration (equal), supervision (equal), writing – review and editing (equal). **Dong Wang:** conceptualization (equal), funding acquisition (equal), supervision (equal), writing – review and editing (equal).

## Conflicts of Interest

The authors declare no conflicts of interest.

## Supporting information


**Figure S1.** Scatter plots depicting the causal relationships between plasma metabolites and sarcopenia‐related traits.

**Figure S2.** The result of leave‐one‐out analysis.

**Table S1.** The causal association between plasma metabolites and sarcopenia‐related traits was initially screened with a threshold of *P*_IVW_ < 0.05.

**Table S2.** The reverse analysis of plasma metabolites and sarcopenia‐related traits was initially screened with a threshold of *P*_IVW_ < 0.05.

**Table S3.** Results of five MR analysis methods.

**Table S4.** MR sensitivity analyses of plasma metabolites and sarcopenia‐related traits as metabolites are the exposure.

**Table S5.** MR sensitivity analyses of plasma metabolites and sarcopenia‐related traits as metabolites are the outcome.

**Table S6.** Sensitivity analysis of the glycine level and sarcopenia‐related traits in validation.

**Table S7.** The genes with the highest overall V2G score related to the SNPs.

## Data Availability

The complete GWAS data can be accessed on the GWAS Catalog server at https://www.ebi.ac.uk/gwas/ (GCST90199621‐90201020, GCST90301967). GWAS summary statistics for SP‐related traits, obtained from the UK Biobank, were downloaded from the IEU Open GWAS project (https://gwas.mrcieu.ac.uk/) (ebi‐a‐GCST90000025, ukb‐b‐10,215, ukb‐b‐7478, ukb‐b‐4711).
