# Supplementary material for: Causal Relationship Between Circulating Metabolites and Sarcopenia‐Related Traits: A Mendelian Randomization and Experimental Study
Source: Food Sci Nutr. 2025 Jan 9;13(1):e4624. doi: 10.1002/fsn3.4624 (PMC11717068; doi:10.1002/fsn3.4624)
Supplement: Supplementary file 1 — Figure S1. Scatter plots depicting the causal relationships between plasma metabolites and sarcopenia‐related traits. [file FSN3-13-e4624-s002.pdf]

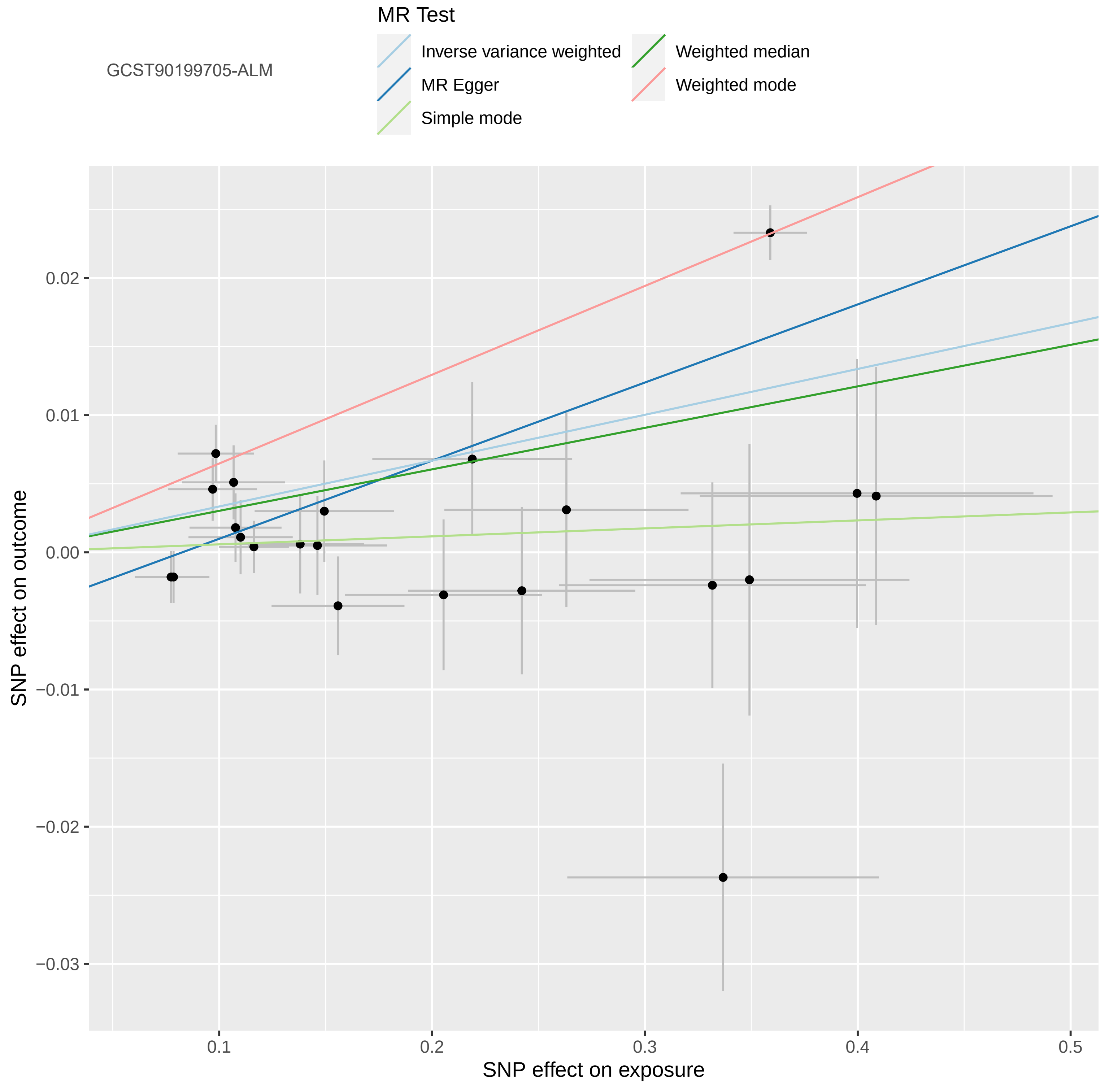

GCST90199705-ALM
MR Test
Inverse variance weighted
MR Egger
Simple mode
Weighted median
Weighted mode
SNP effect on outcome
SNP effect on exposure
0.02
0.01
0.00
−0.01
−0.02
−0.03
0.1
0.2
0.3
0.4
0.5

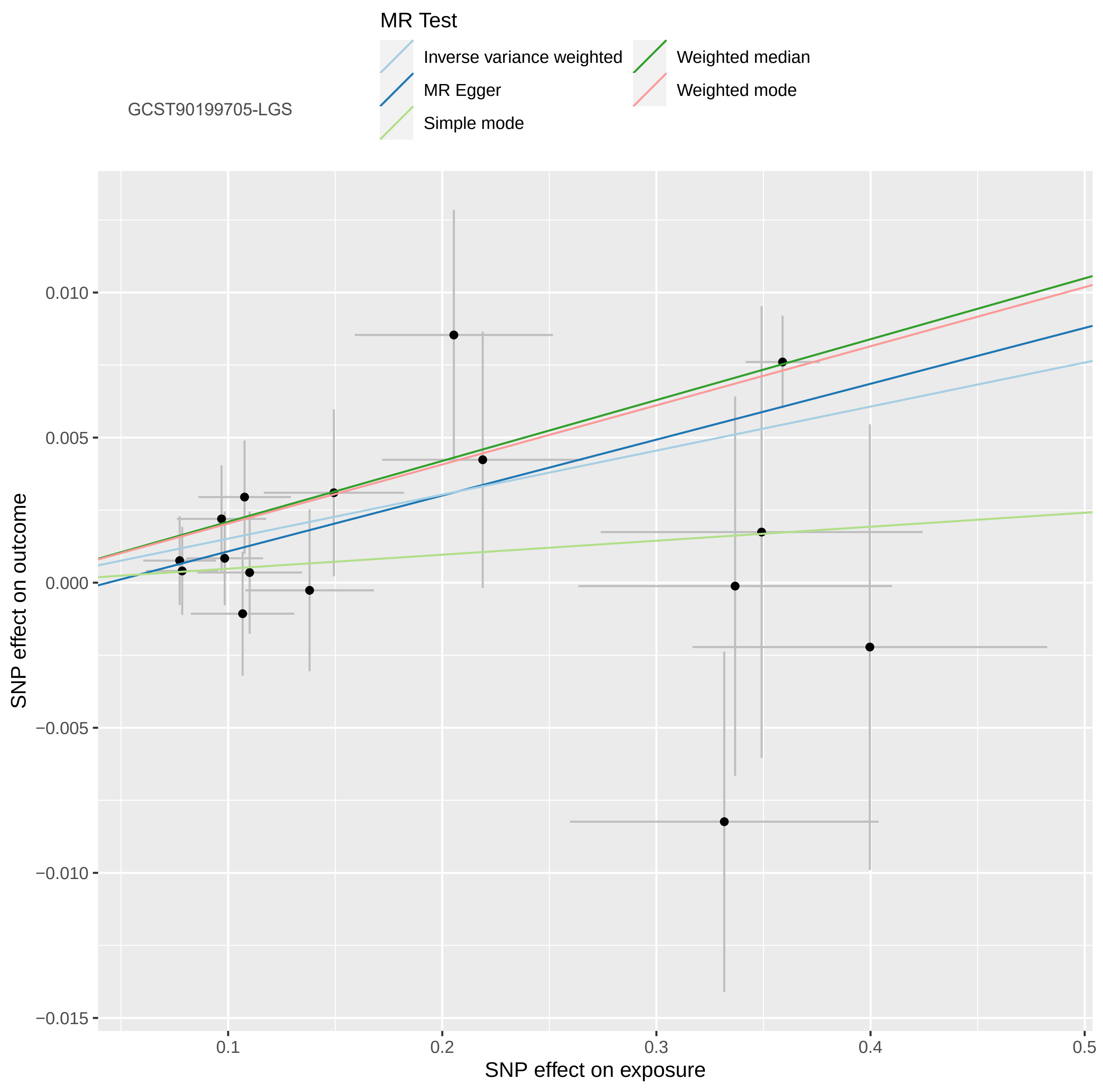

MR Test
Inverse variance weighted
MR Egger
Simple mode
Weighted median
Weighted mode
GCST90199705-LGS
0.010
0.005
0.000
−0.005
−0.010
−0.015
SNP effect on outcome
0.1
0.2
0.3
0.4
0.5
SNP effect on exposure

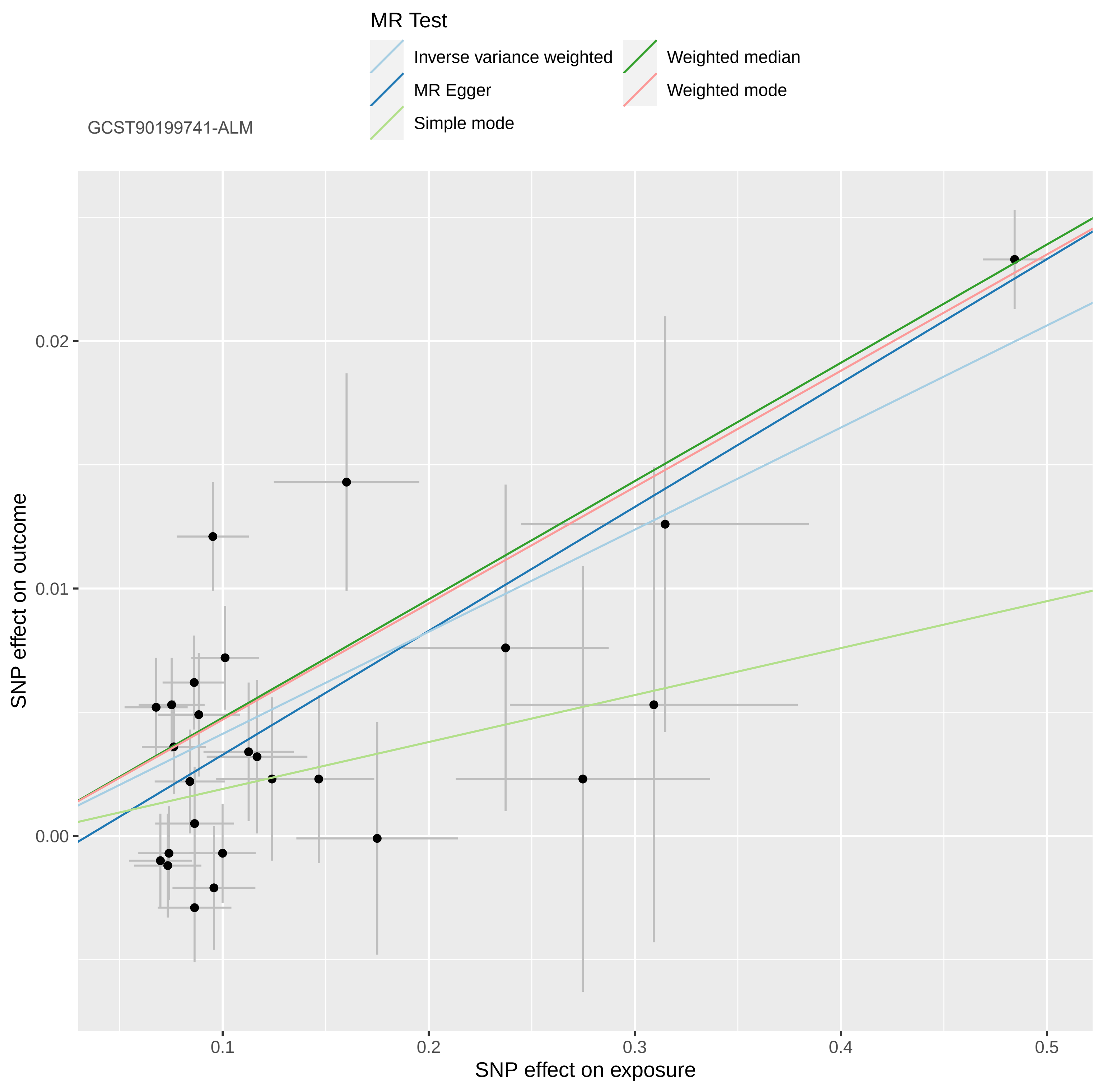

MR Test
Inverse variance weighted
MR Egger
Simple mode
Weighted median
Weighted mode
GCST90199741-ALM
SNP effect on outcome
0.02
0.01
0.00
0.1
0.2
0.3
0.4
0.5
SNP effect on exposure

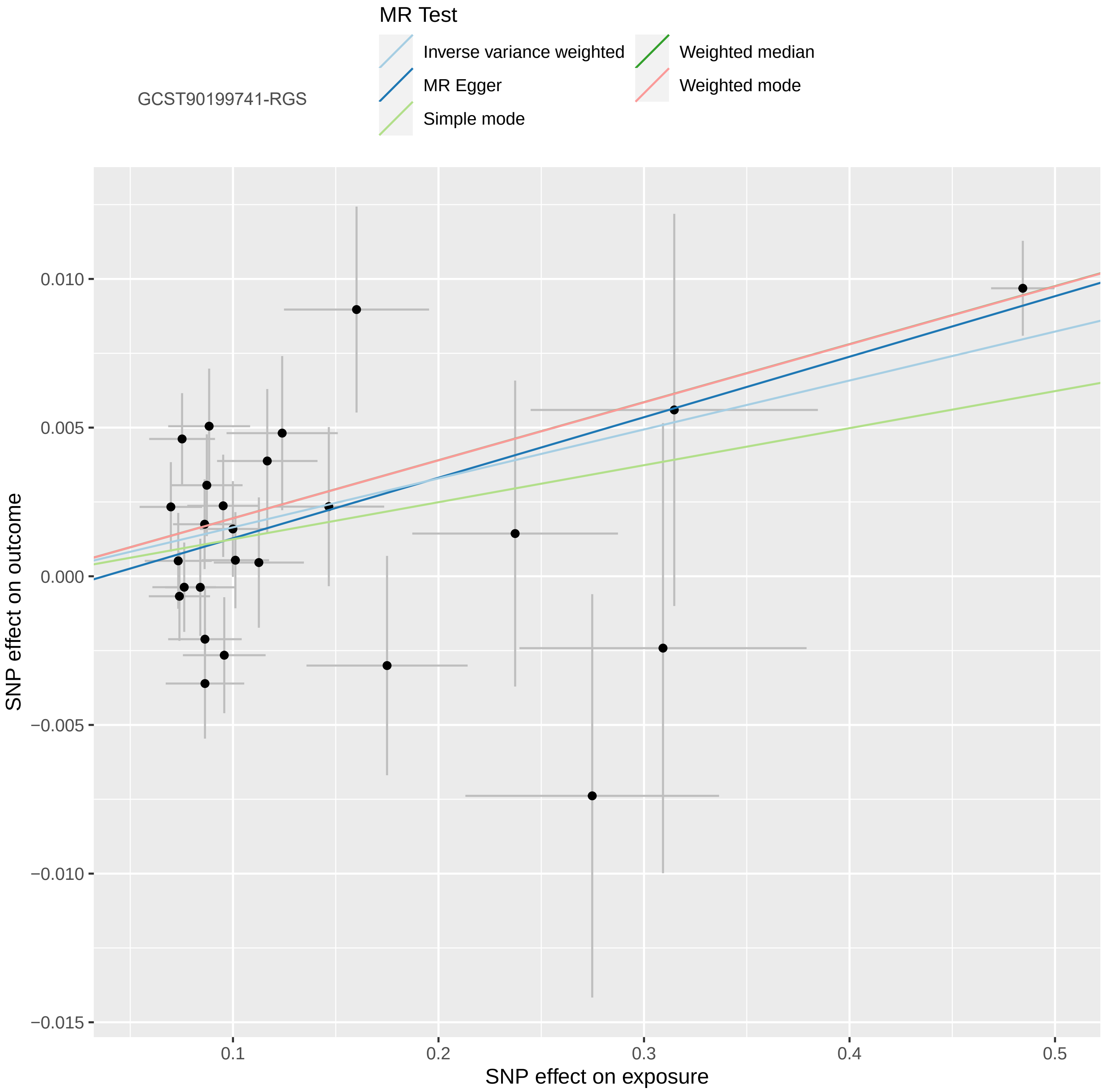
MR Test
Inverse variance weighted
MR Egger
Simple mode
Weighted median
Weighted mode
GCST90199741-RGS
SNP effect on outcome
SNP effect on exposure
0.010
0.005
0.000
−0.005
−0.010
−0.015
0.1
0.2
0.3
0.4
0.5

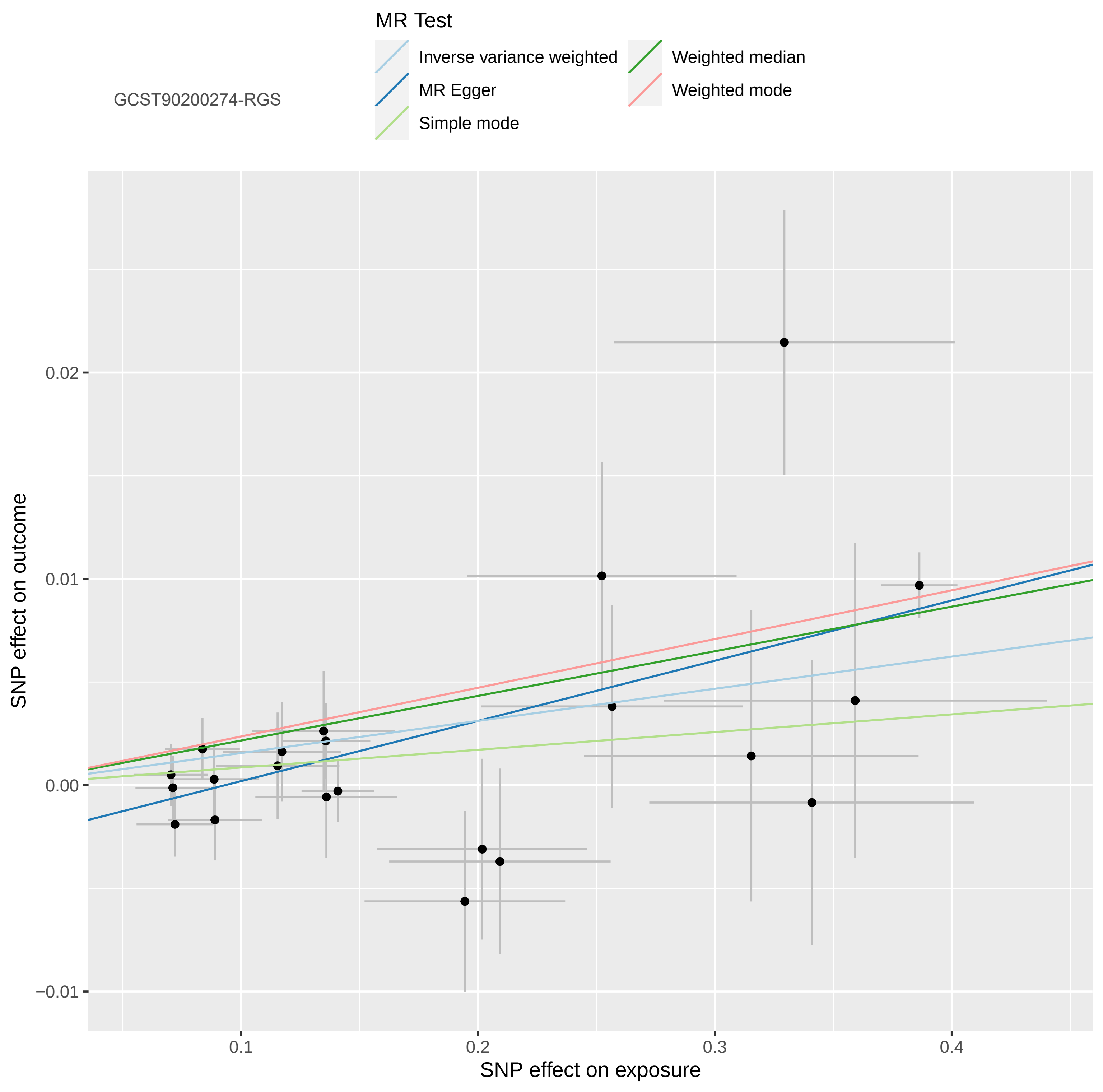

MR Test
Inverse variance weighted
MR Egger
Simple mode
Weighted median
Weighted mode
GCST90200274-RGS
SNP effect on outcome
0.02
0.01
0.00
−0.01
SNP effect on exposure
0.1
0.2
0.3
0.4

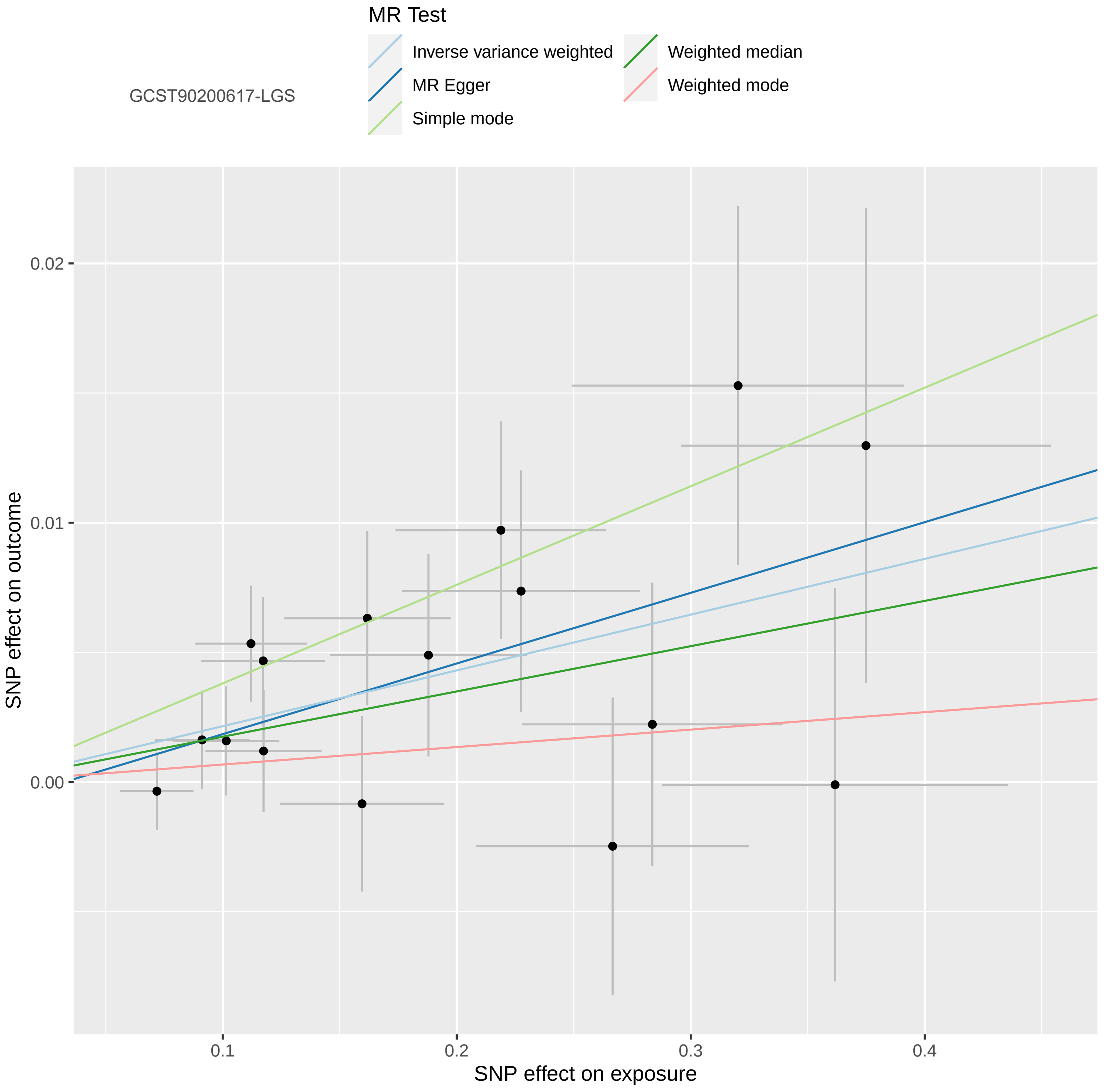

MR Test
Inverse variance weighted
MR Egger
Simple mode
Weighted median
Weighted mode
GCST90200617-LGS
SNP effect on outcome
0.02
0.01
0.00
0.1
0.2
0.3
0.4
SNP effect on exposure

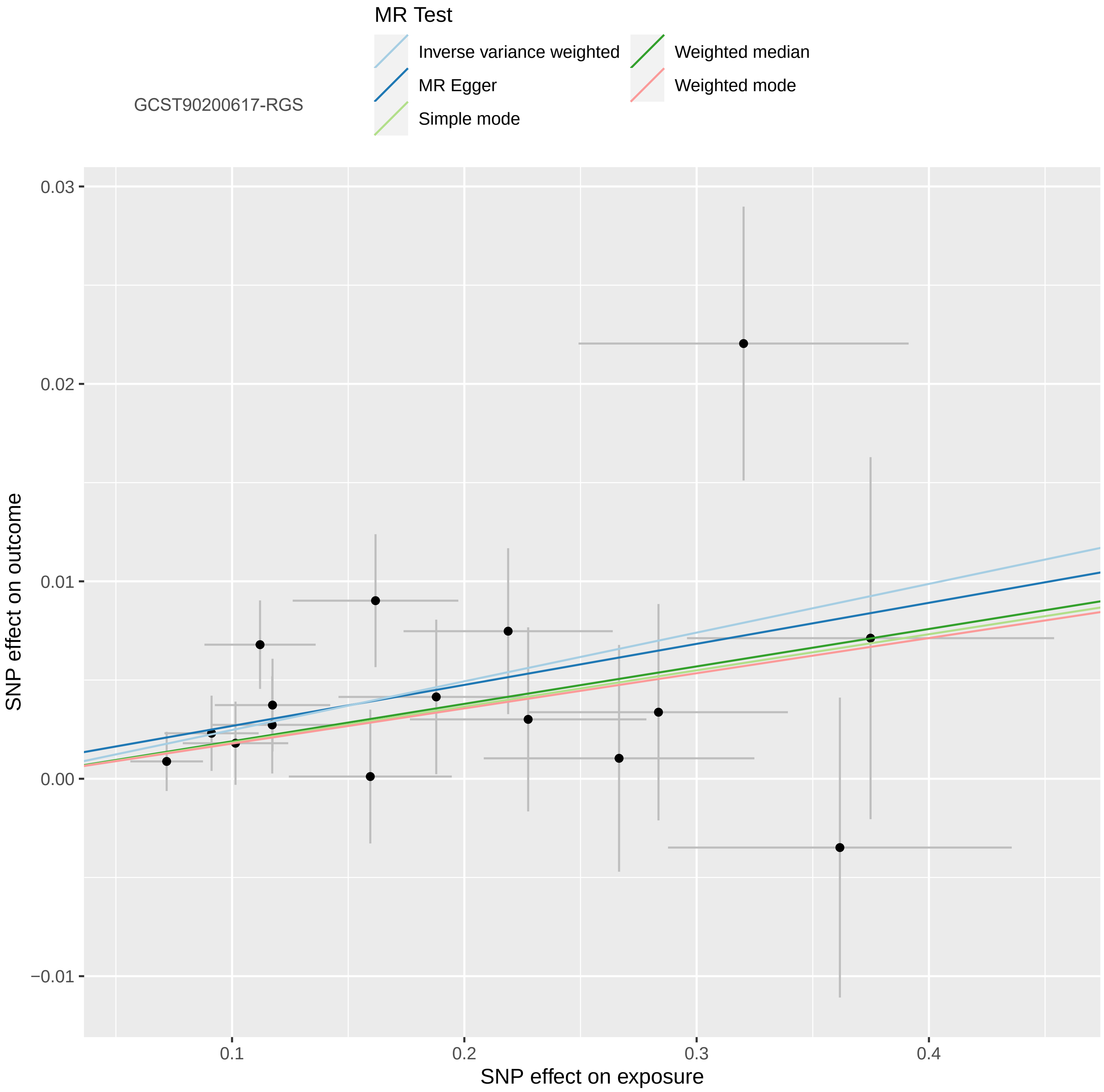

MR Test
Inverse variance weighted
MR Egger
Simple mode
Weighted median
Weighted mode
GCST90200617-RGS
0.03
0.02
0.01
0.00
−0.01
0.1
0.2
0.3
0.4
SNP effect on outcome
SNP effect on exposure

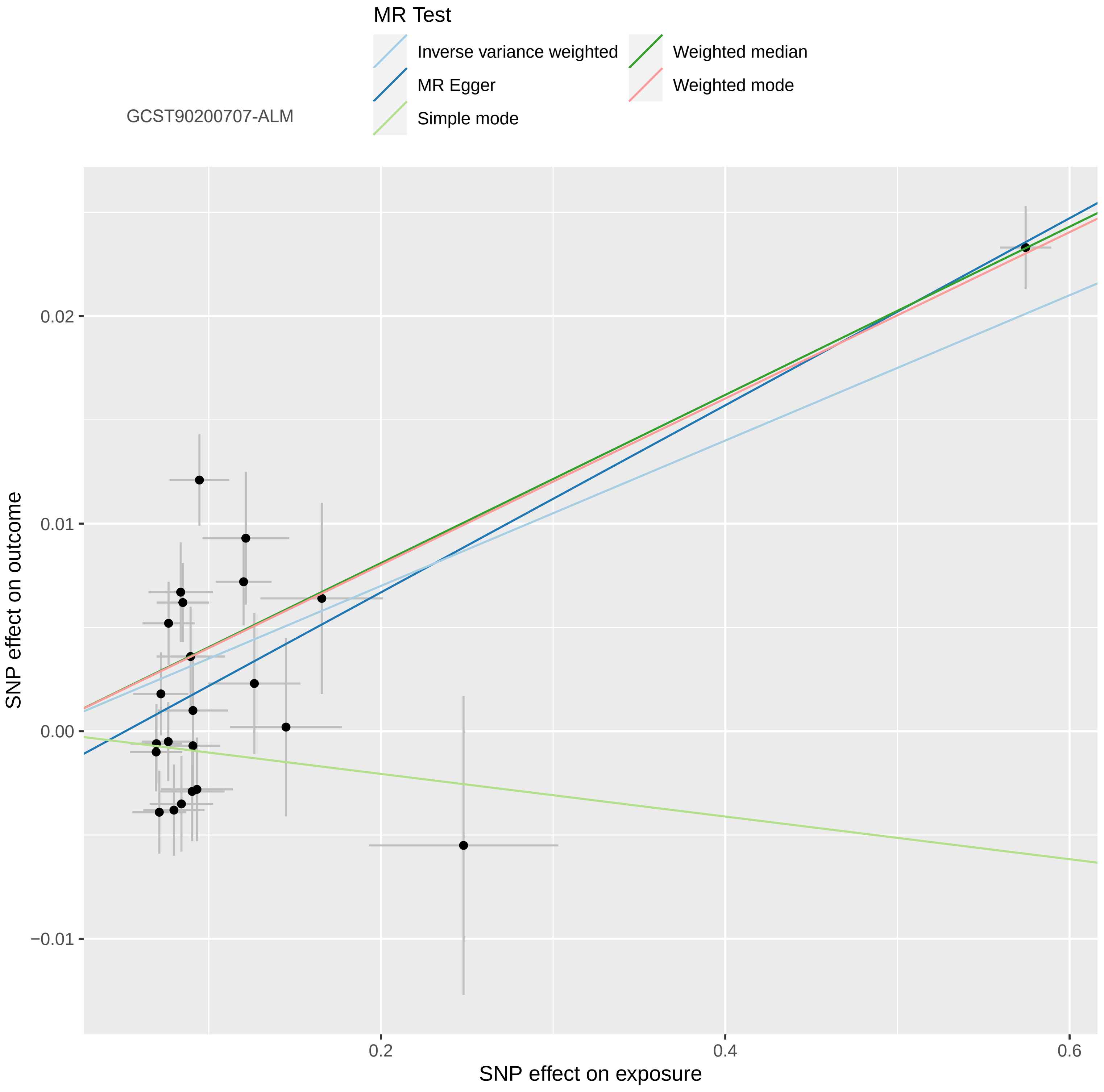

MR Test
Inverse variance weighted
MR Egger
Simple mode
Weighted median
Weighted mode
GCST90200707-ALM
SNP effect on outcome
0.02
0.01
0.00
−0.01
0.2
0.4
0.6
SNP effect on exposure

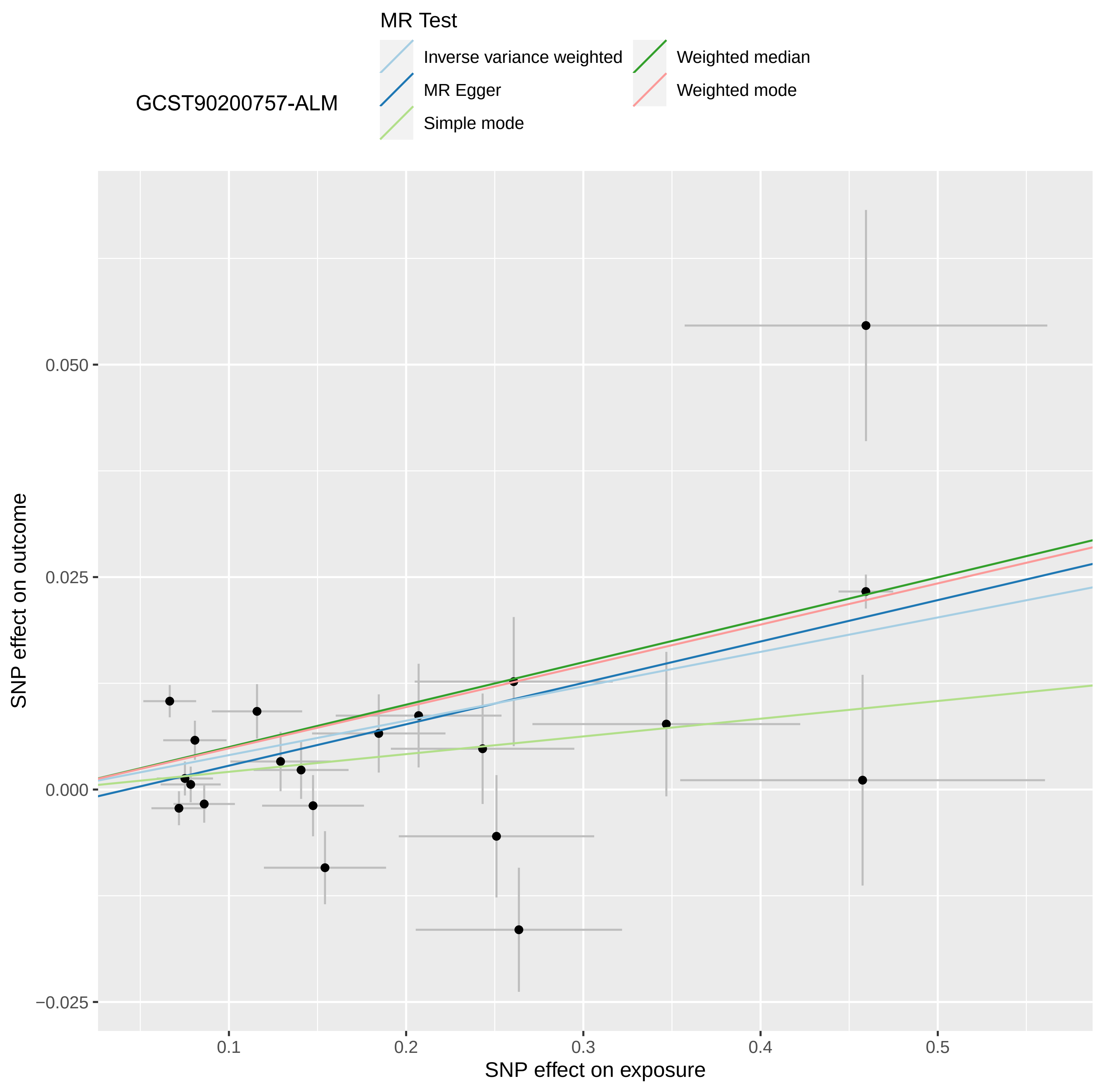

GCST90200757-ALM
MR Test
Inverse variance weighted
MR Egger
Simple mode
Weighted median
Weighted mode
SNP effect on outcome
SNP effect on exposure
0.050
0.025
0.000
−0.025
0.1
0.2
0.3
0.4
0.5

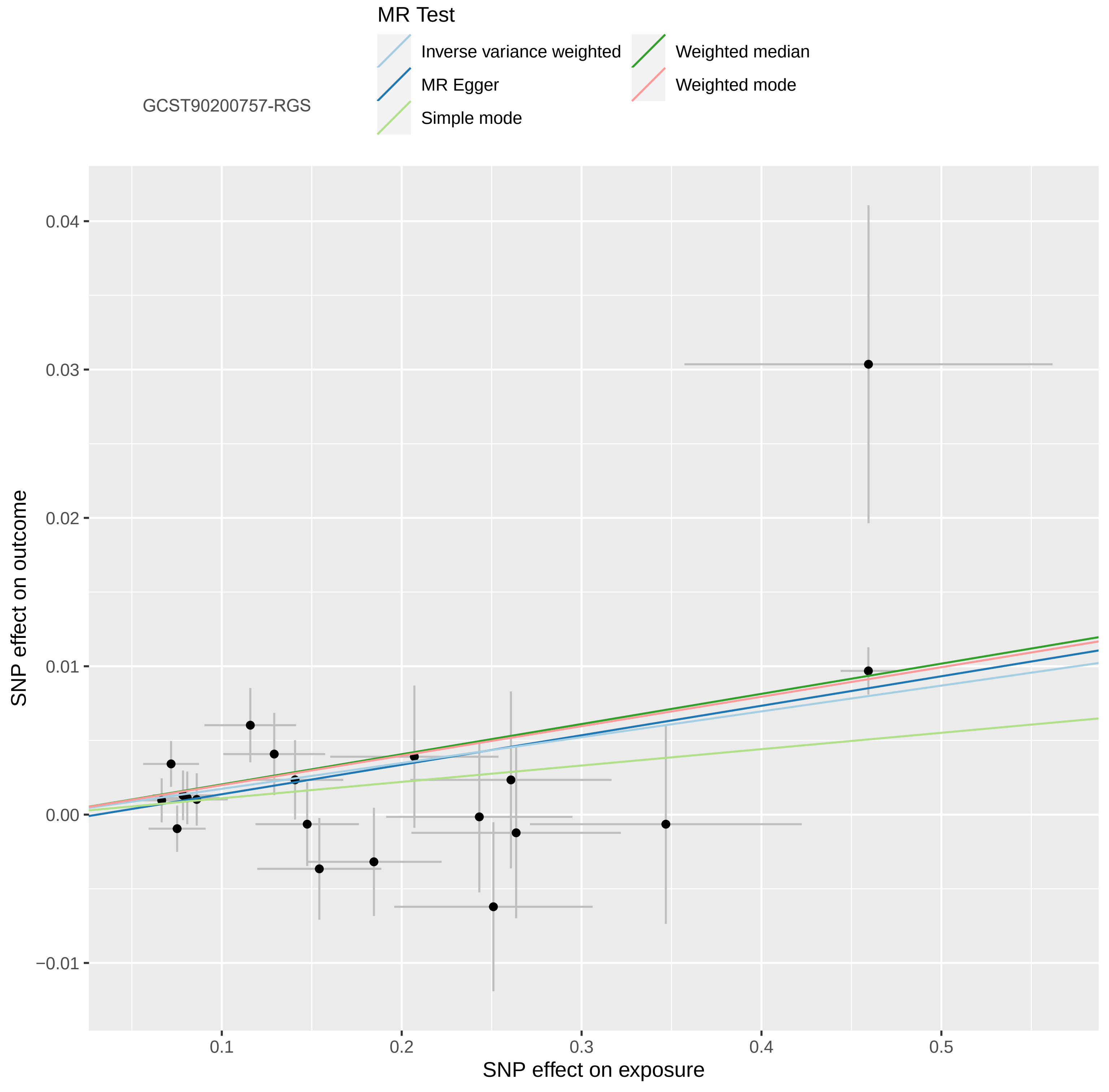

MR Test
Inverse variance weighted
MR Egger
Simple mode
Weighted median
Weighted mode
GCST90200757-RGS
SNP effect on outcome
SNP effect on exposure
0.04
0.03
0.02
0.01
0.00
−0.01
0.1
0.2
0.3
0.4
0.5

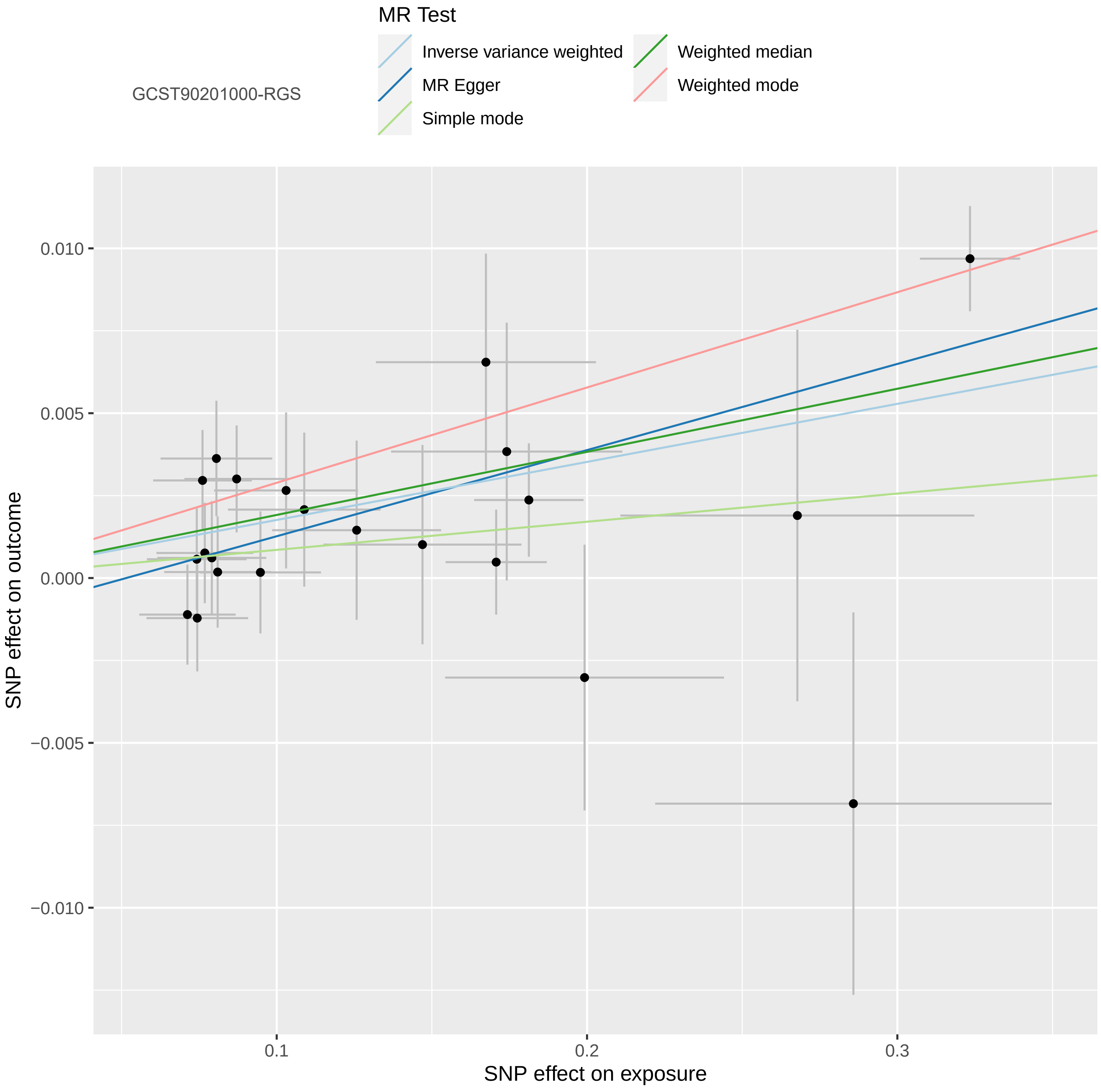
MR Test
Inverse variance weighted
MR Egger
Simple mode
Weighted median
Weighted mode
GCST90201000-RGS
0.010
0.005
0.000
−0.005
−0.010
SNP effect on outcome
0.1
0.2
0.3
SNP effect on exposure

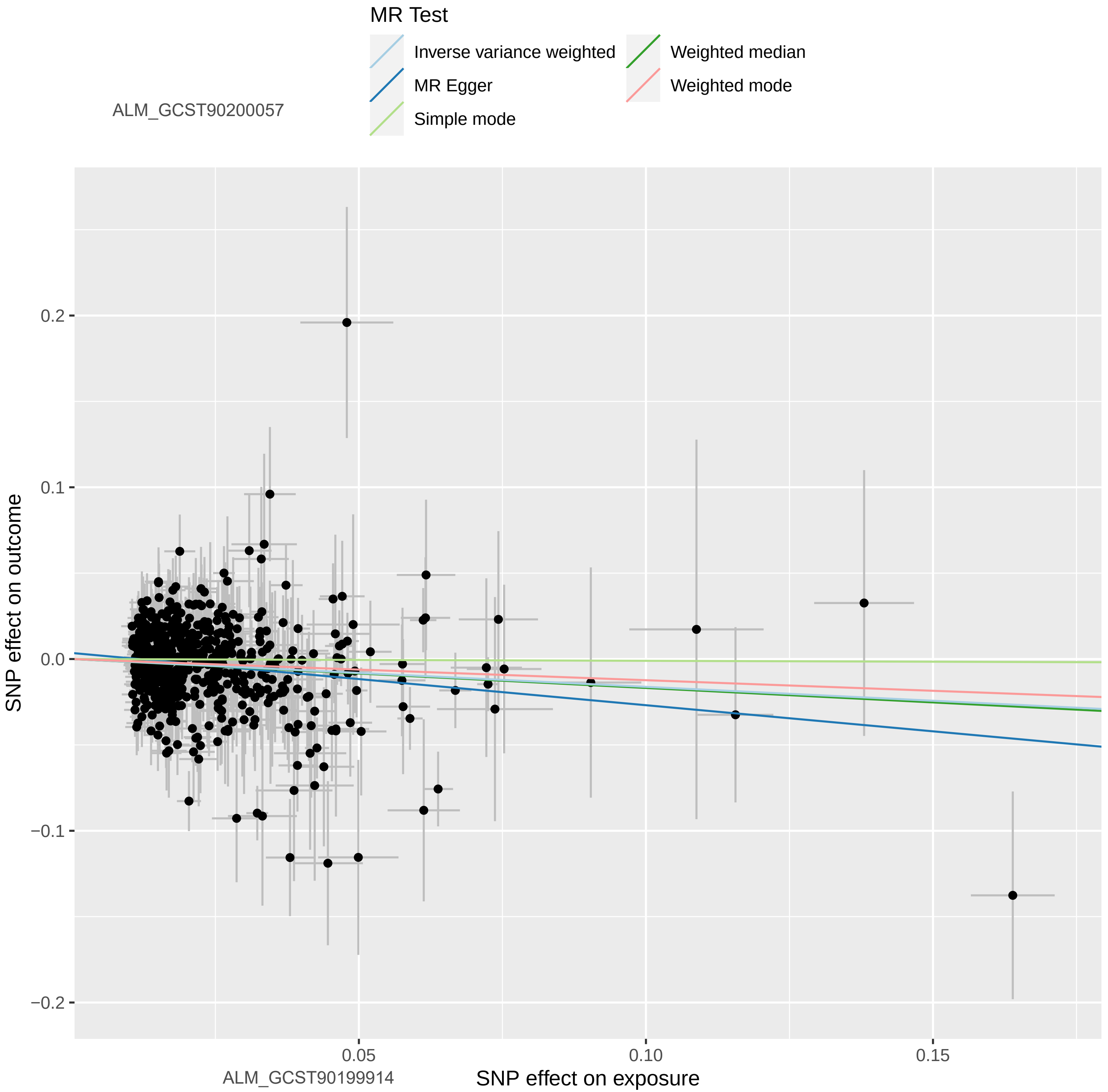
MR Test
Inverse variance weighted
MR Egger
Simple mode
Weighted median
Weighted mode
ALM_GCST90200057
0.2
0.1
0.0
−0.1
−0.2
SNP effect on outcome
0.05
0.10
0.15
ALM_GCST90199914
SNP effect on exposure

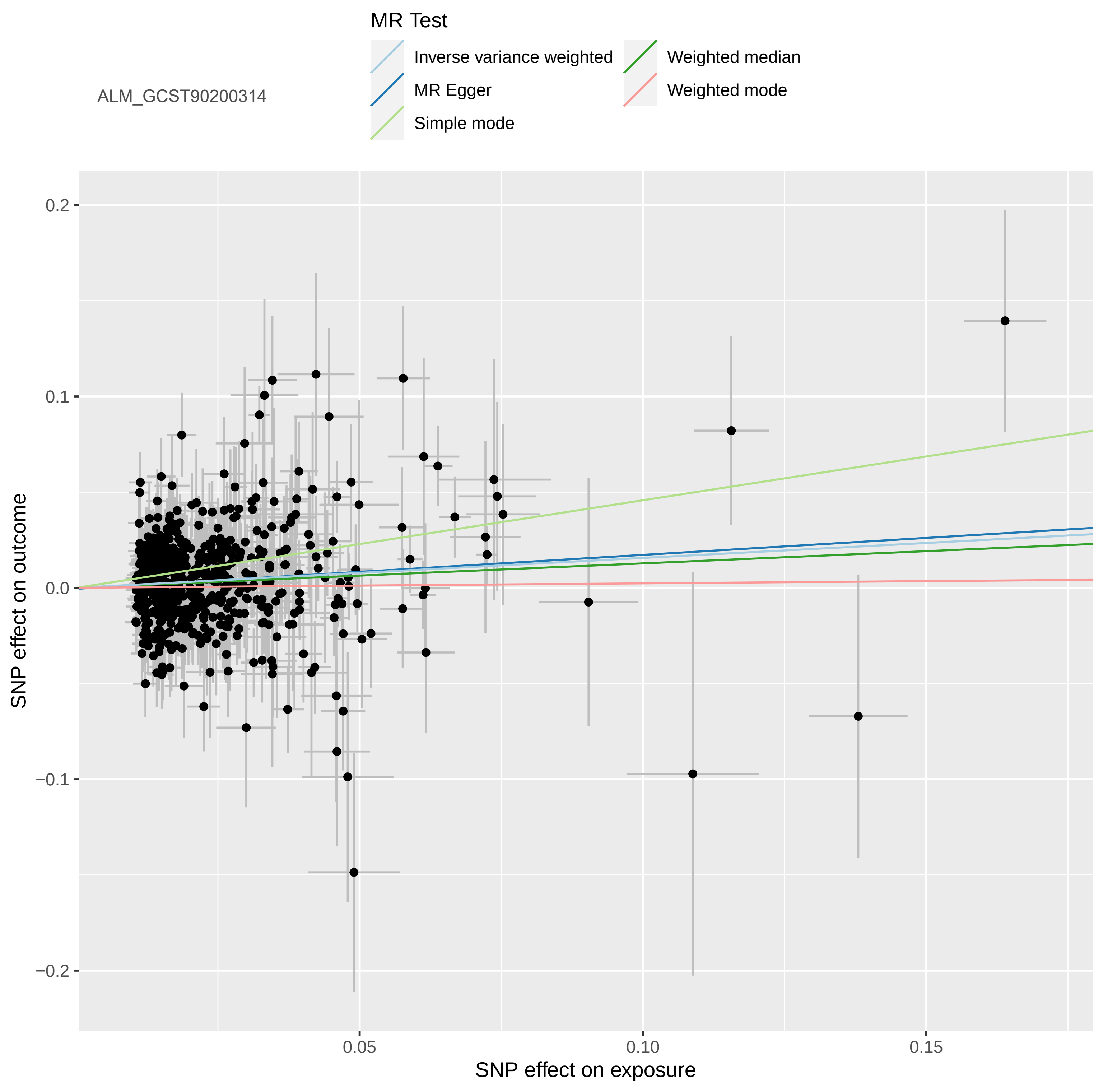

ALM_GCST90200314
MR Test
Inverse variance weighted
MR Egger
Simple mode
Weighted median
Weighted mode
0.2
0.1
0.0
−0.1
−0.2
SNP effect on outcome
0.05
0.10
0.15
SNP effect on exposure

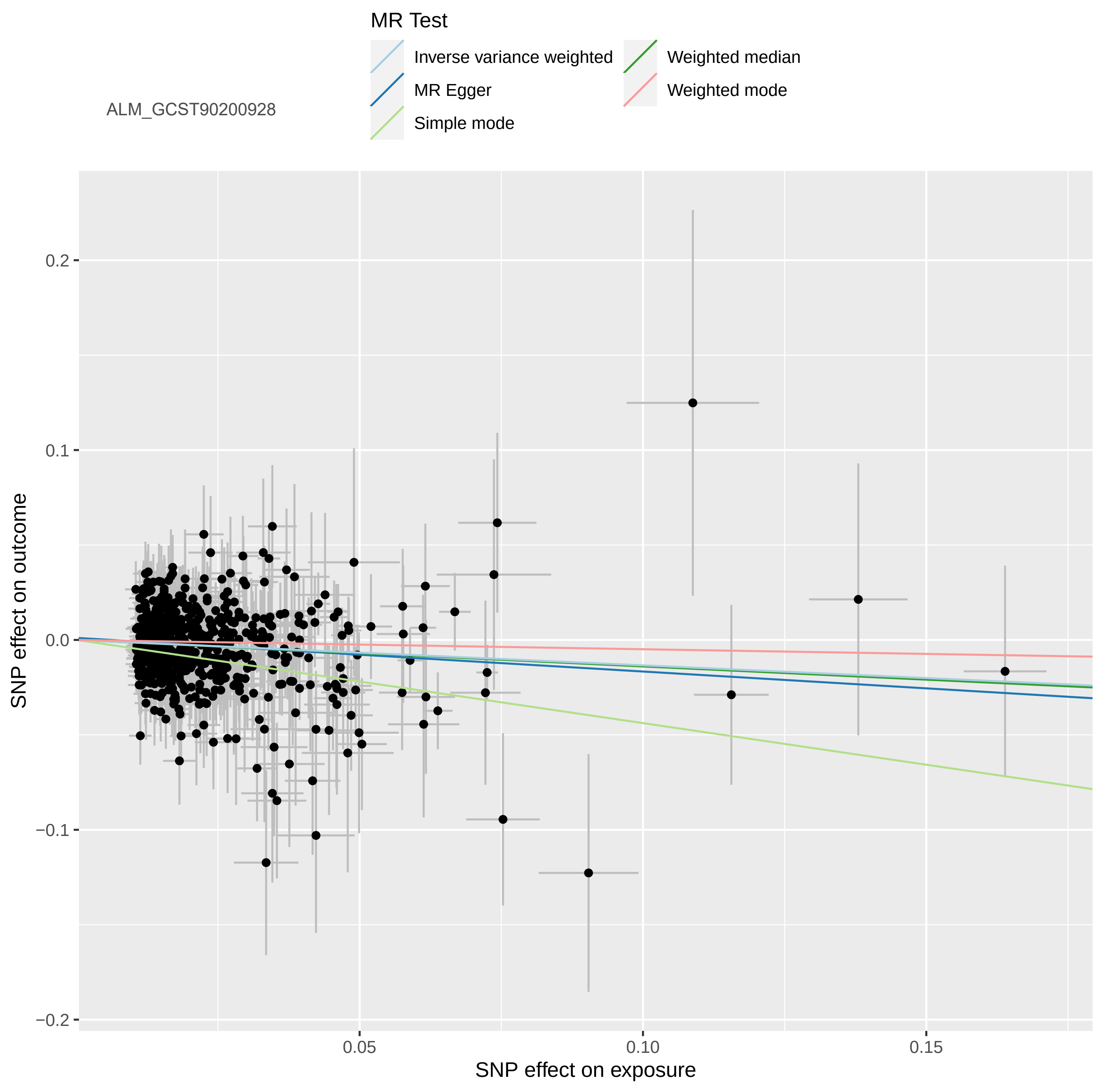

MR Test
Inverse variance weighted
MR Egger
Simple mode
Weighted median
Weighted mode
ALM_GCST90200928
0.2
0.1
0.0
−0.1
−0.2
SNP effect on outcome
0.05
0.10
0.15
SNP effect on exposure

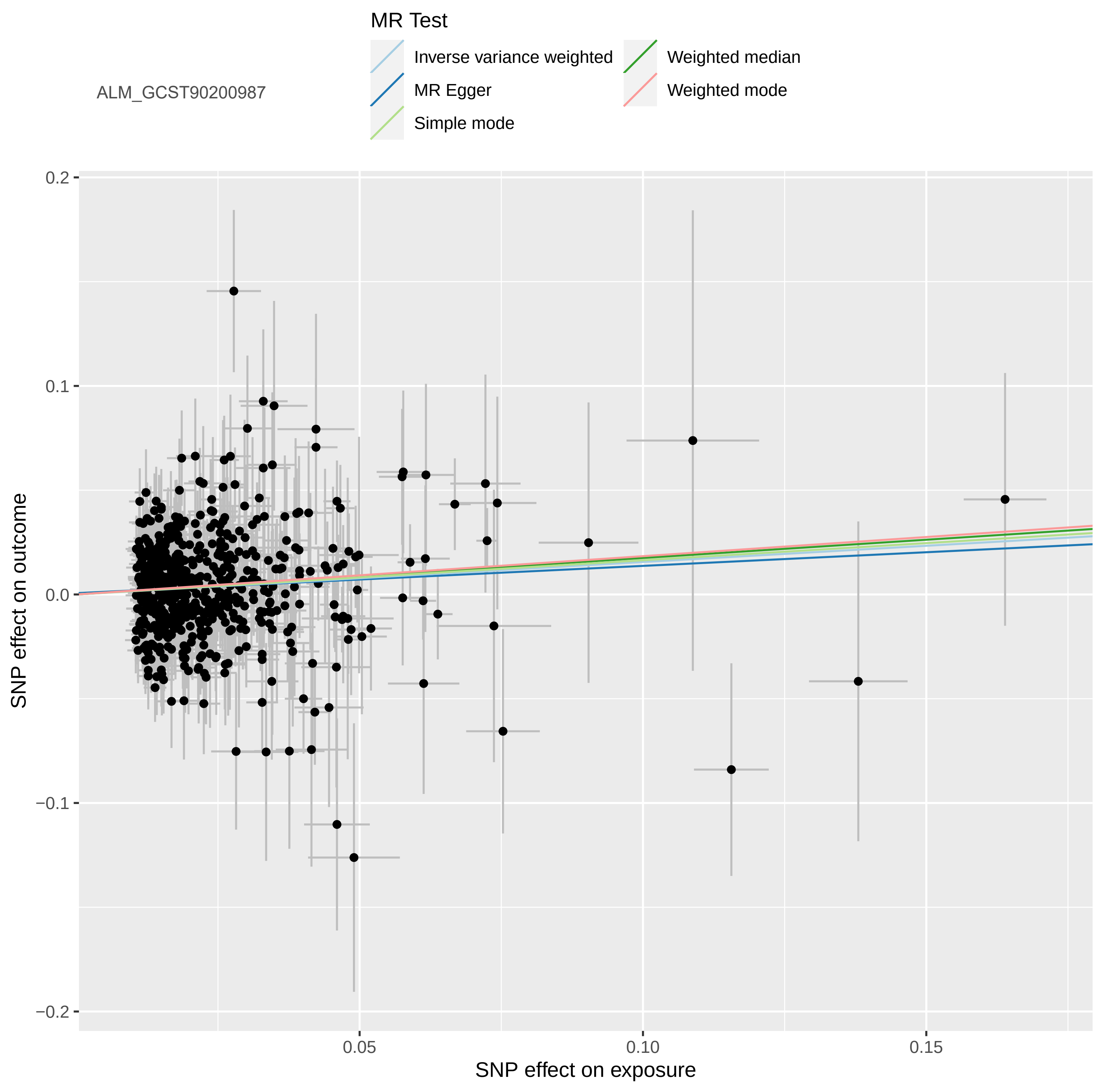

ALM_GCST90200987
MR Test
Inverse variance weighted
MR Egger
Simple mode
Weighted median
Weighted mode
0.2
0.1
0.0
−0.1
−0.2
SNP effect on outcome
0.05
0.10
0.15
SNP effect on exposure

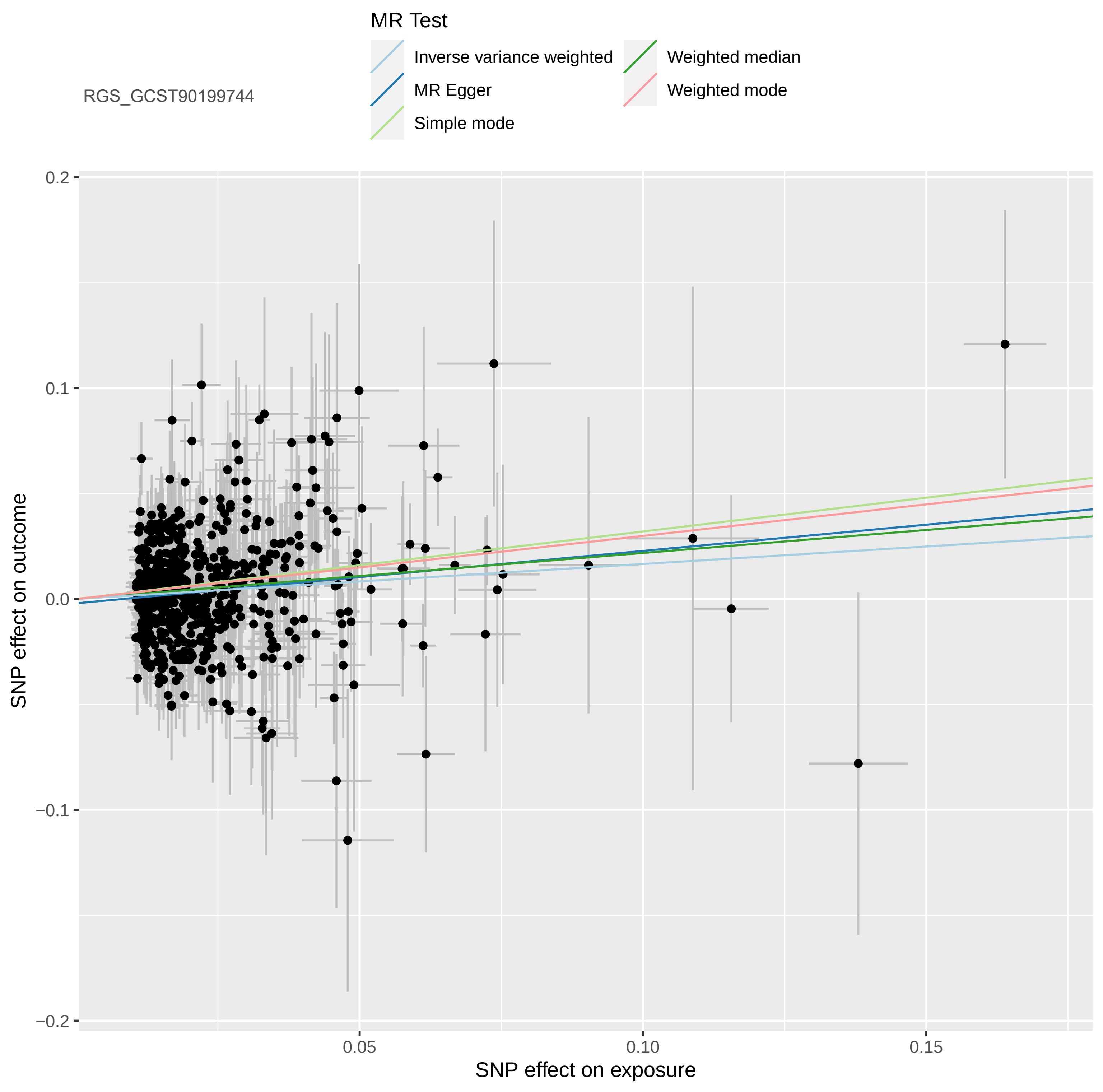

MR Test
Inverse variance weighted
MR Egger
Simple mode
Weighted median
Weighted mode
RGS_GCST90199744
0.2
0.1
0.0
−0.1
−0.2
SNP effect on outcome
0.05
0.10
0.15
SNP effect on exposure

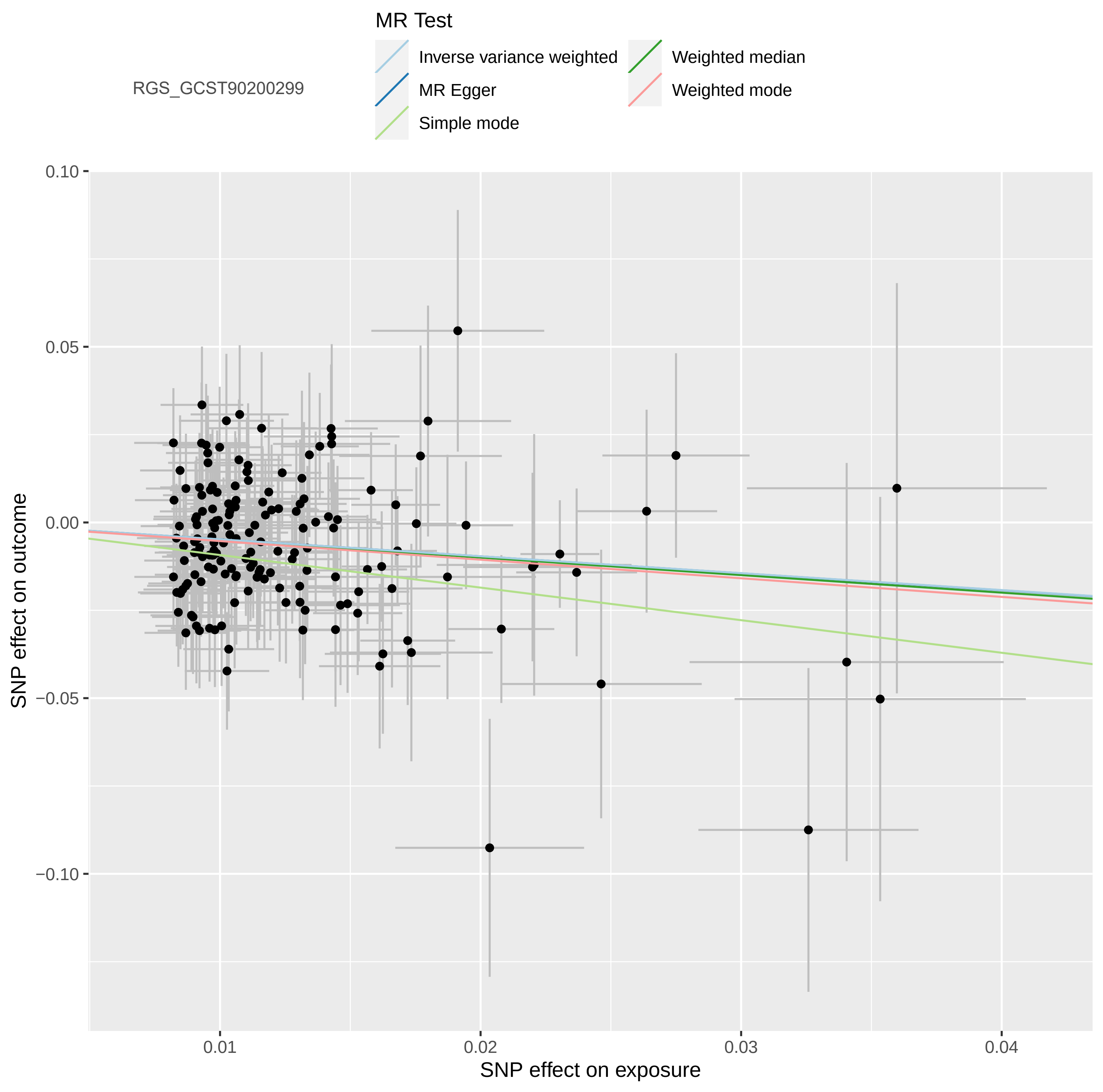
MR Test
Inverse variance weighted
MR Egger
Simple mode
Weighted median
Weighted mode
RGS_GCST90200299
0.10
0.05
0.00
−0.05
−0.10
0.01
0.02
0.03
0.04
SNP effect on outcome
SNP effect on exposure

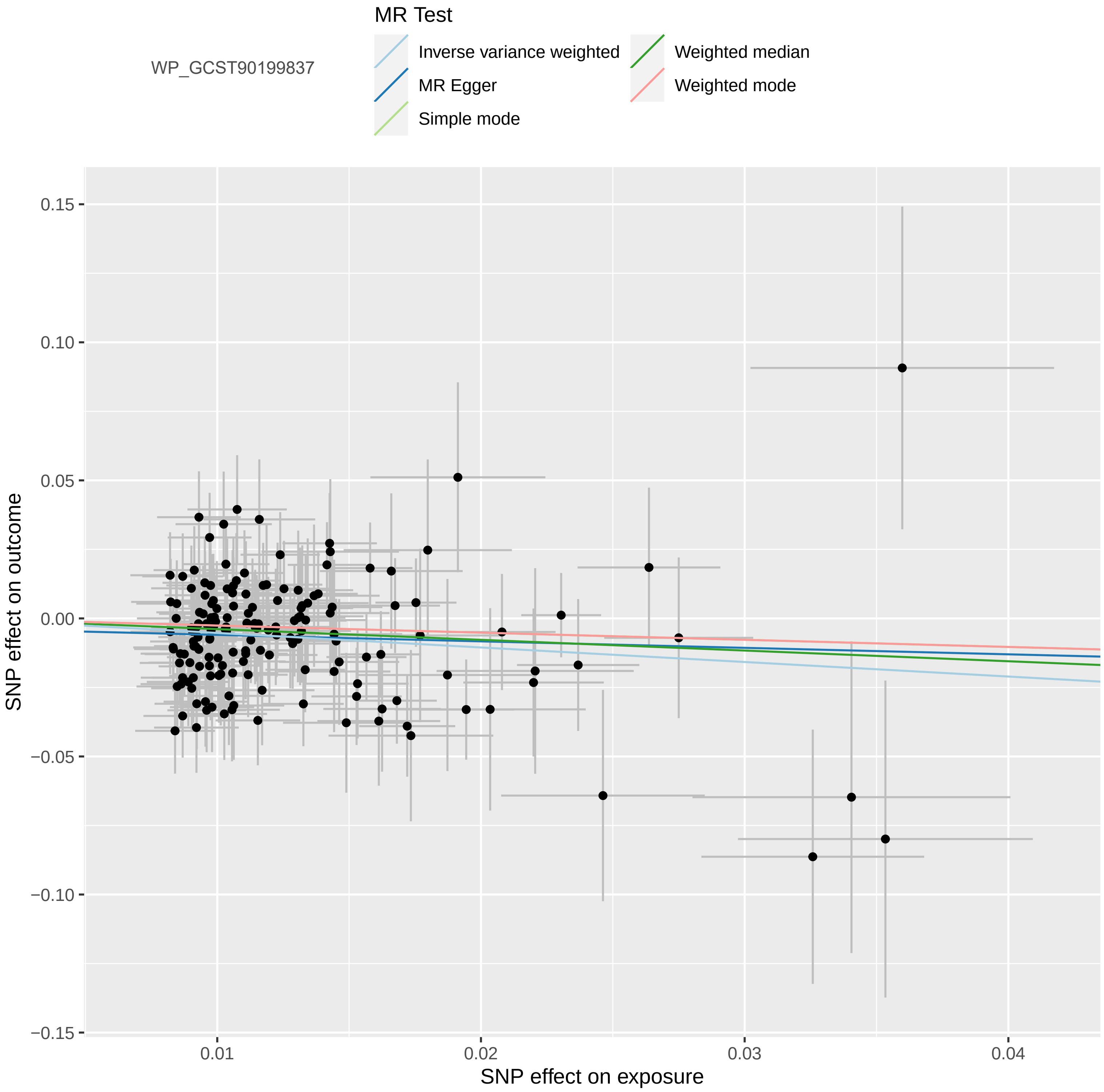
WP_GCST90199837
MR Test
Inverse variance weighted
MR Egger
Simple mode
Weighted median
Weighted mode
SNP effect on outcome
SNP effect on exposure
0.15
0.10
0.05
0.00
−0.05
−0.10
−0.15
0.01
0.02
0.03
0.04

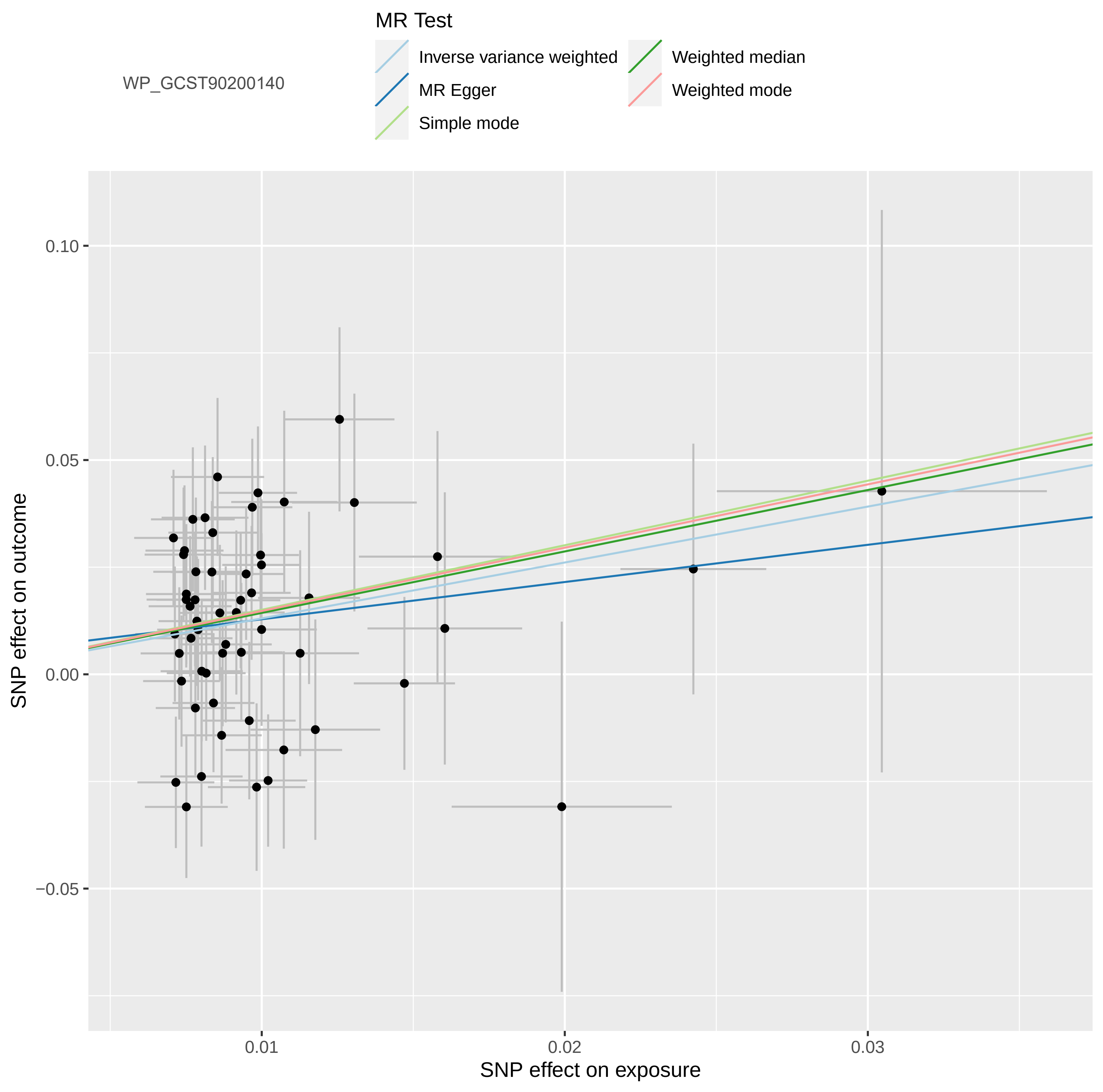

MR Test
Inverse variance weighted
MR Egger
Simple mode
Weighted median
Weighted mode
WP_GCST90200140
SNP effect on outcome
SNP effect on exposure
0.10
0.05
0.00
−0.05
0.01
0.02
0.03

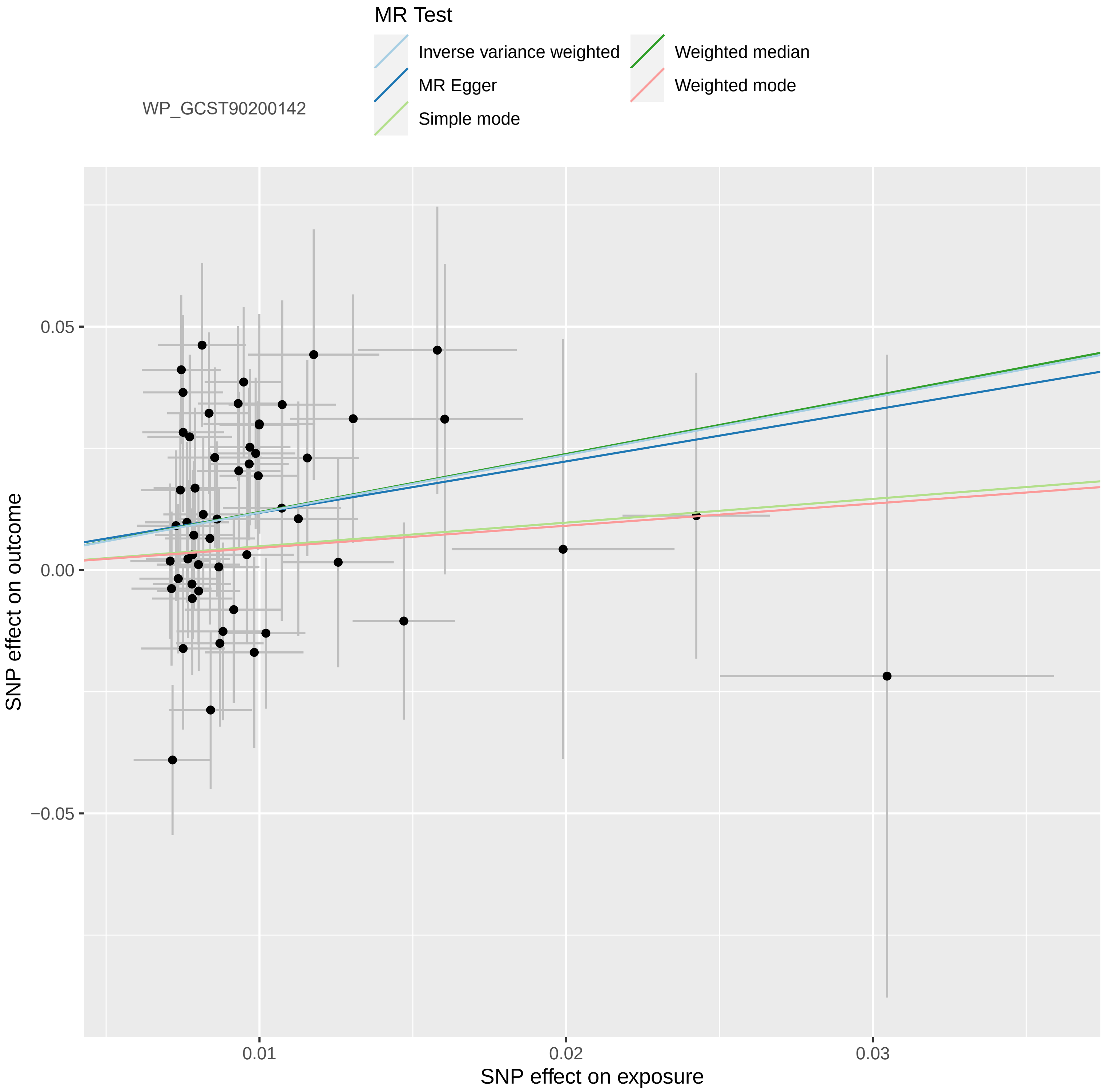
MR Test
Inverse variance weighted
MR Egger
Simple mode
Weighted median
Weighted mode
WP_GCST90200142
0.05
0.00
−0.05
0.01
0.02
0.03
SNP effect on outcome
SNP effect on exposure

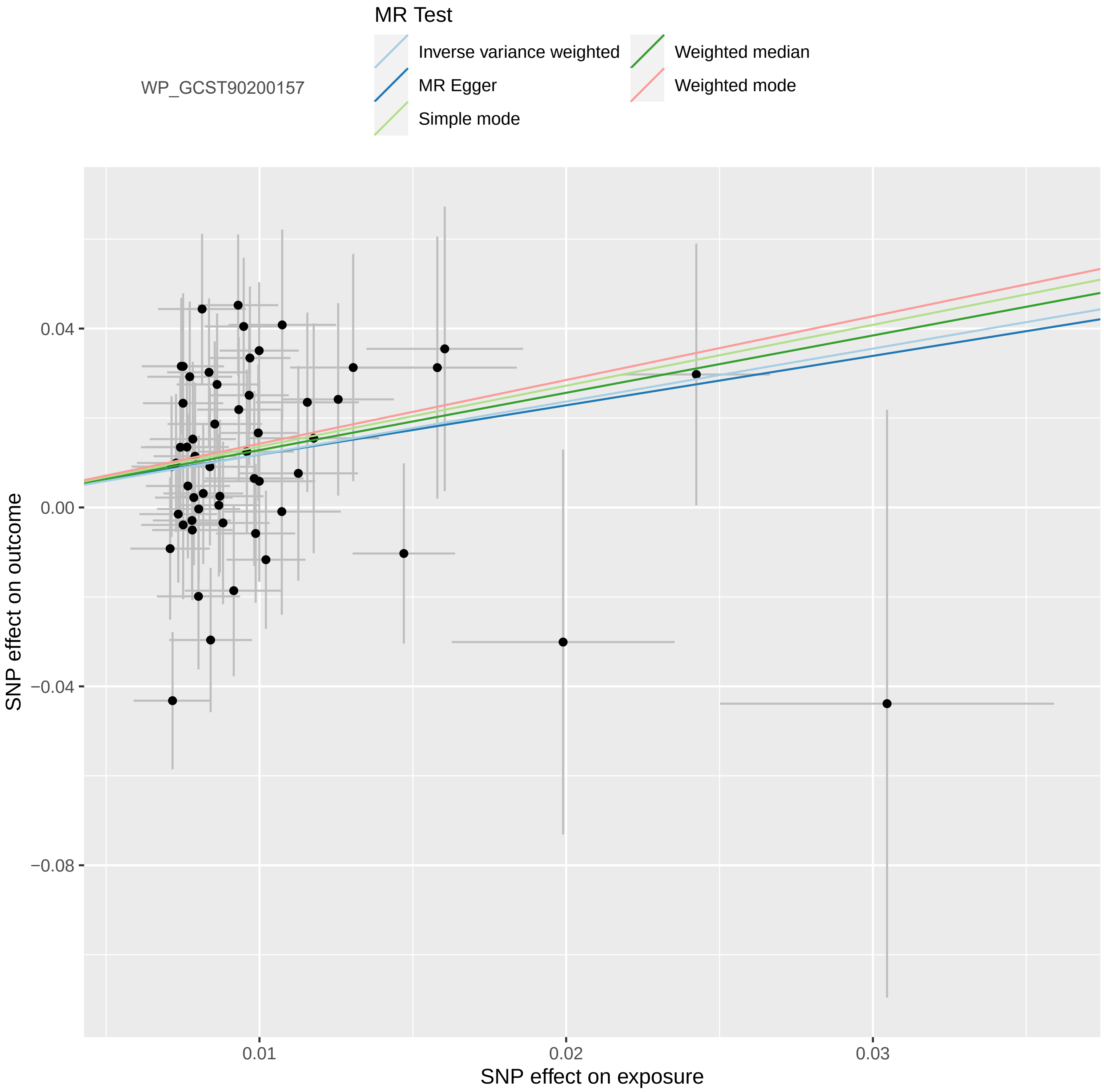

MR Test
Inverse variance weighted
MR Egger
Simple mode
Weighted median
Weighted mode
WP_GCST90200157
0.04
0.00
−0.04
−0.08
0.01
0.02
0.03
SNP effect on outcome
SNP effect on exposure

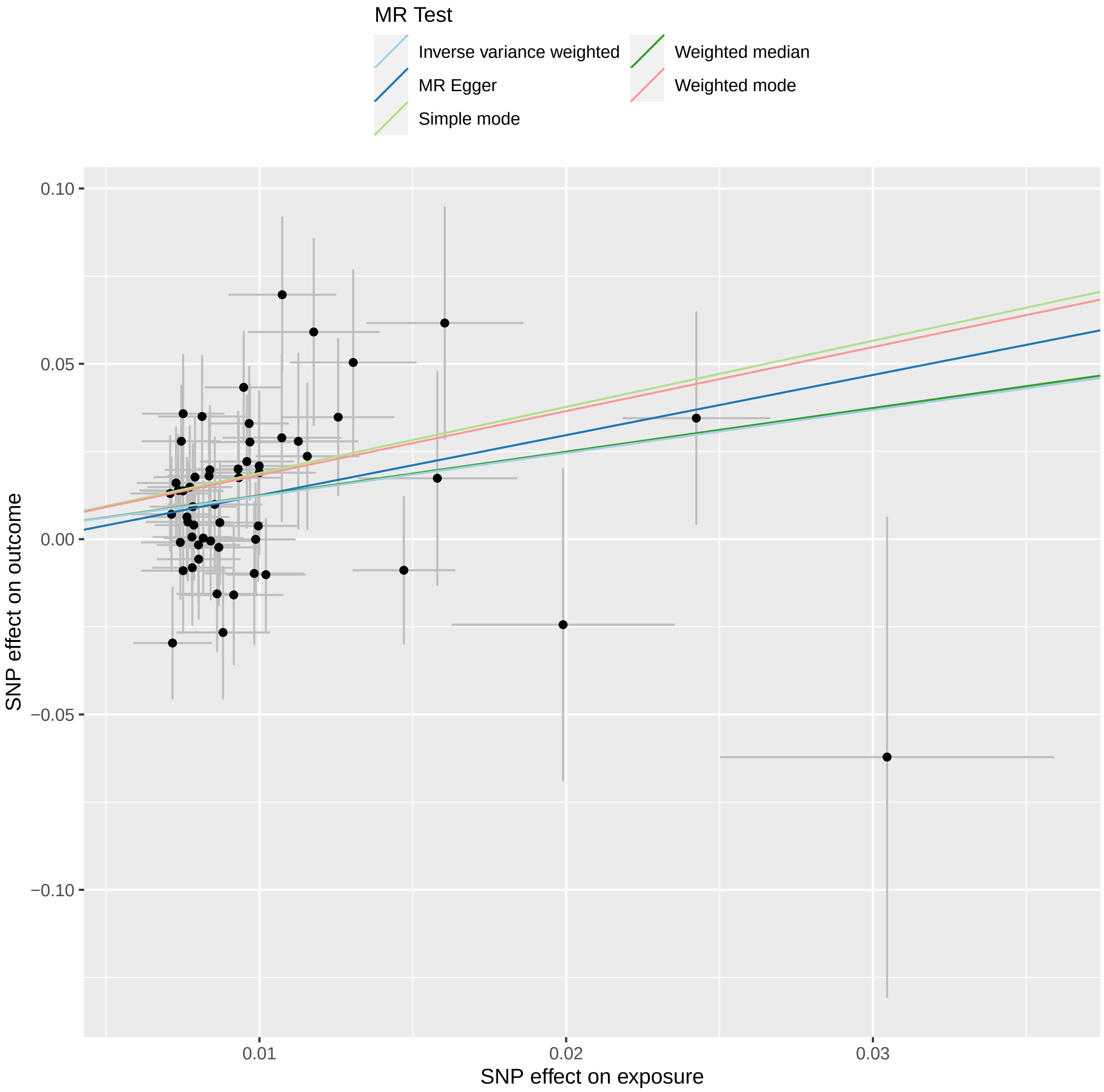
MR Test
Inverse variance weighted
MR Egger
Simple mode
Weighted median
Weighted mode
0.10
0.05
0.00
−0.05
−0.10
SNP effect on outcome
0.01
0.02
0.03
SNP effect on exposure
