# Supplementary material for: Causal Relationship Between Circulating Metabolites and Sarcopenia‐Related Traits: A Mendelian Randomization and Experimental Study
Source: Food Sci Nutr. 2025 Jan 9;13(1):e4624. doi: 10.1002/fsn3.4624 (PMC11717068; doi:10.1002/fsn3.4624)
Supplement: Supplementary file 2 — Figure S2. The result of leave‐one‐out analysis. [file FSN3-13-e4624-s001.pdf]

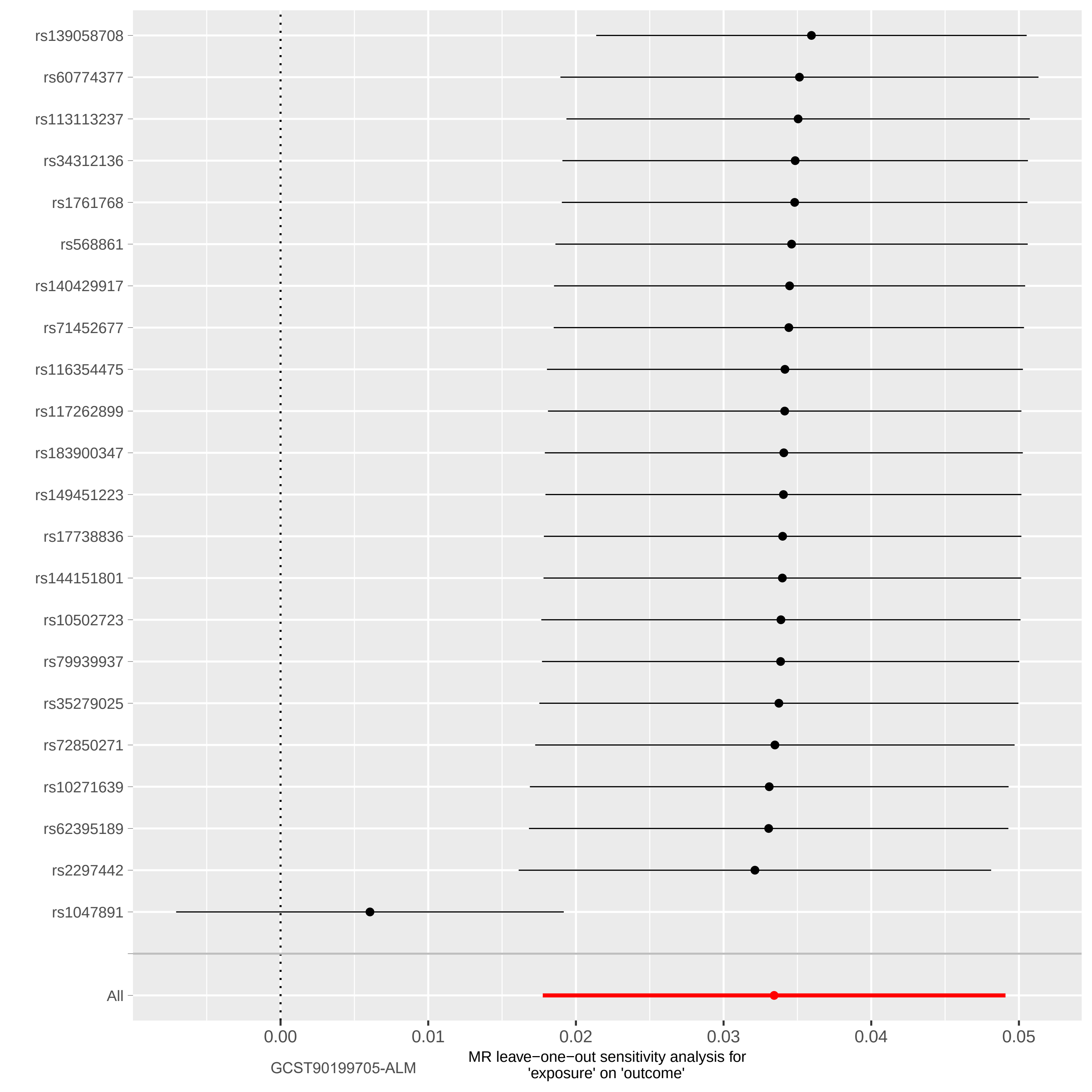

rs139058708
rs60774377
rs113113237
rs34312136
rs1761768
rs568861
rs140429917
rs71452677
rs116354475
rs117262899
rs183900347
rs149451223
rs17738836
rs144151801
rs10502723
rs79939937
rs35279025
rs72850271
rs10271639
rs62395189
rs2297442
rs1047891
All
0.00
0.01
0.02
0.03
0.04
0.05
GCST90199705-ALM
MR leave−one−out sensitivity analysis for
'exposure' on 'outcome'

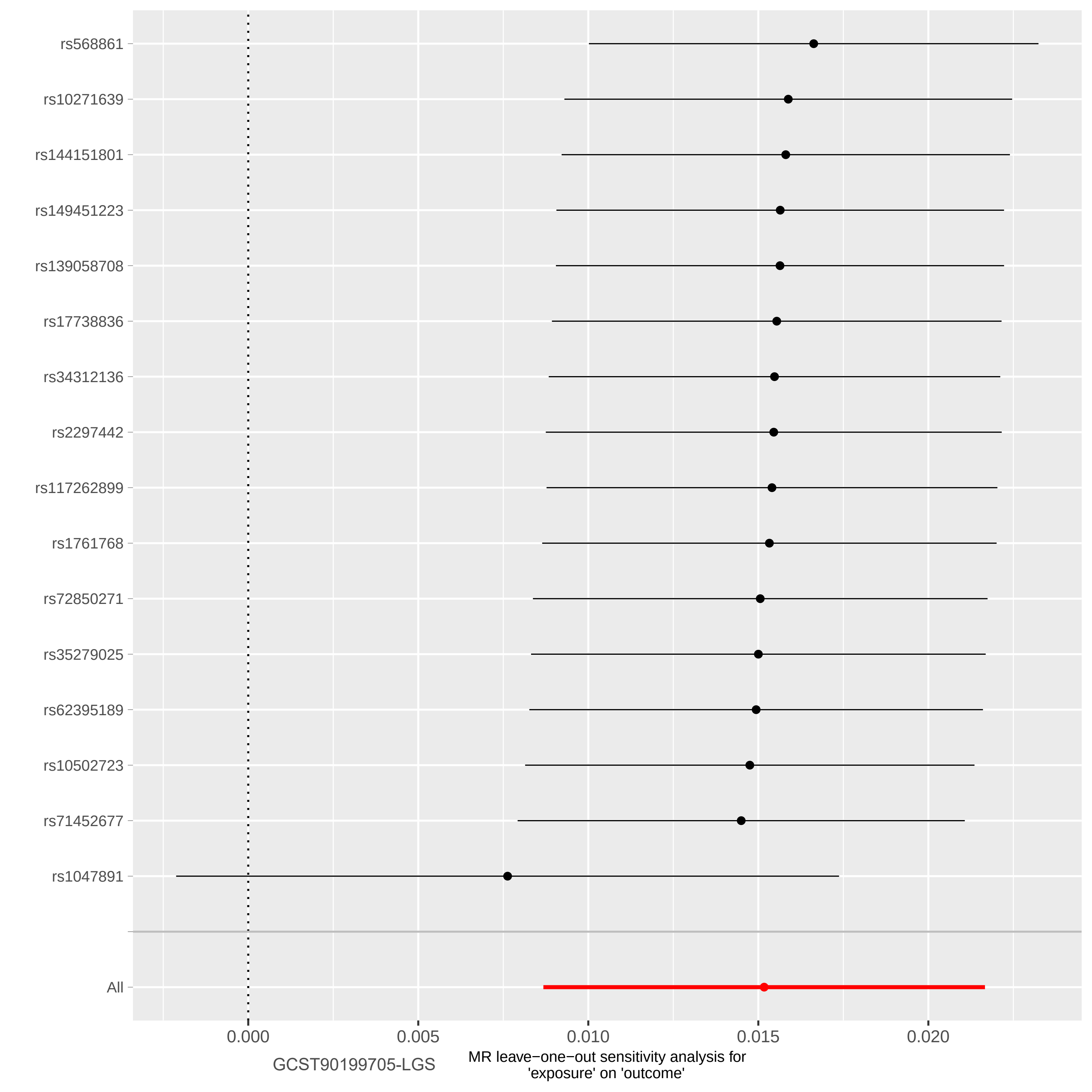
rs568861
rs10271639
rs144151801
rs149451223
rs139058708
rs17738836
rs34312136
rs2297442
rs117262899
rs1761768
rs72850271
rs35279025
rs62395189
rs10502723
rs71452677
rs1047891
All
0.000
0.005
0.010
0.015
0.020
GCST90199705-LGS
MR leave−one−out sensitivity analysis for
'exposure' on 'outcome'

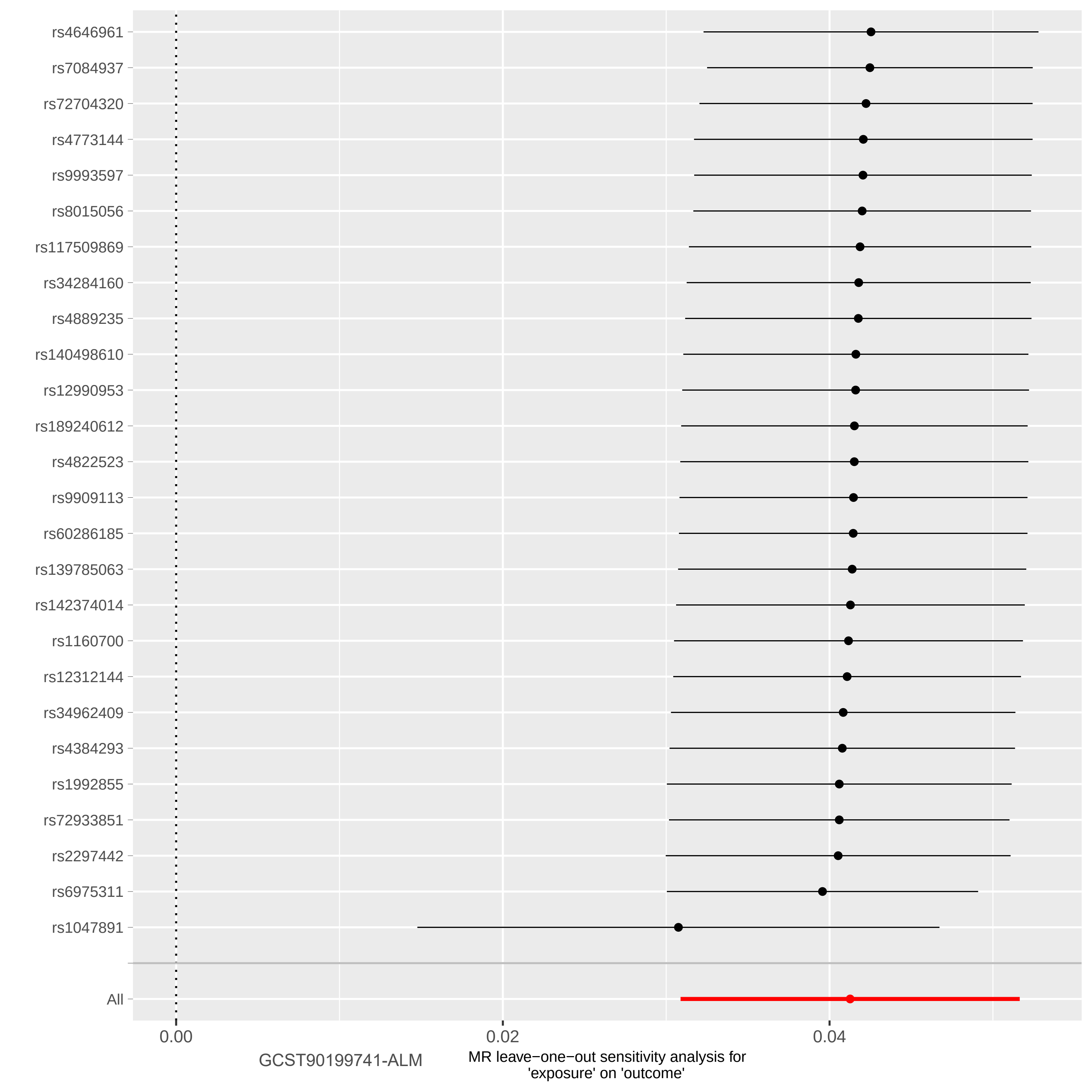
rs4646961
rs7084937
rs72704320
rs4773144
rs9993597
rs8015056
rs117509869
rs34284160
rs4889235
rs140498610
rs12990953
rs189240612
rs4822523
rs9909113
rs60286185
rs139785063
rs142374014
rs1160700
rs12312144
rs34962409
rs4384293
rs1992855
rs72933851
rs2297442
rs6975311
rs1047891
All
0.00
0.02
0.04
GCST90199741-ALM
MR leave−one−out sensitivity analysis for
'exposure' on 'outcome'

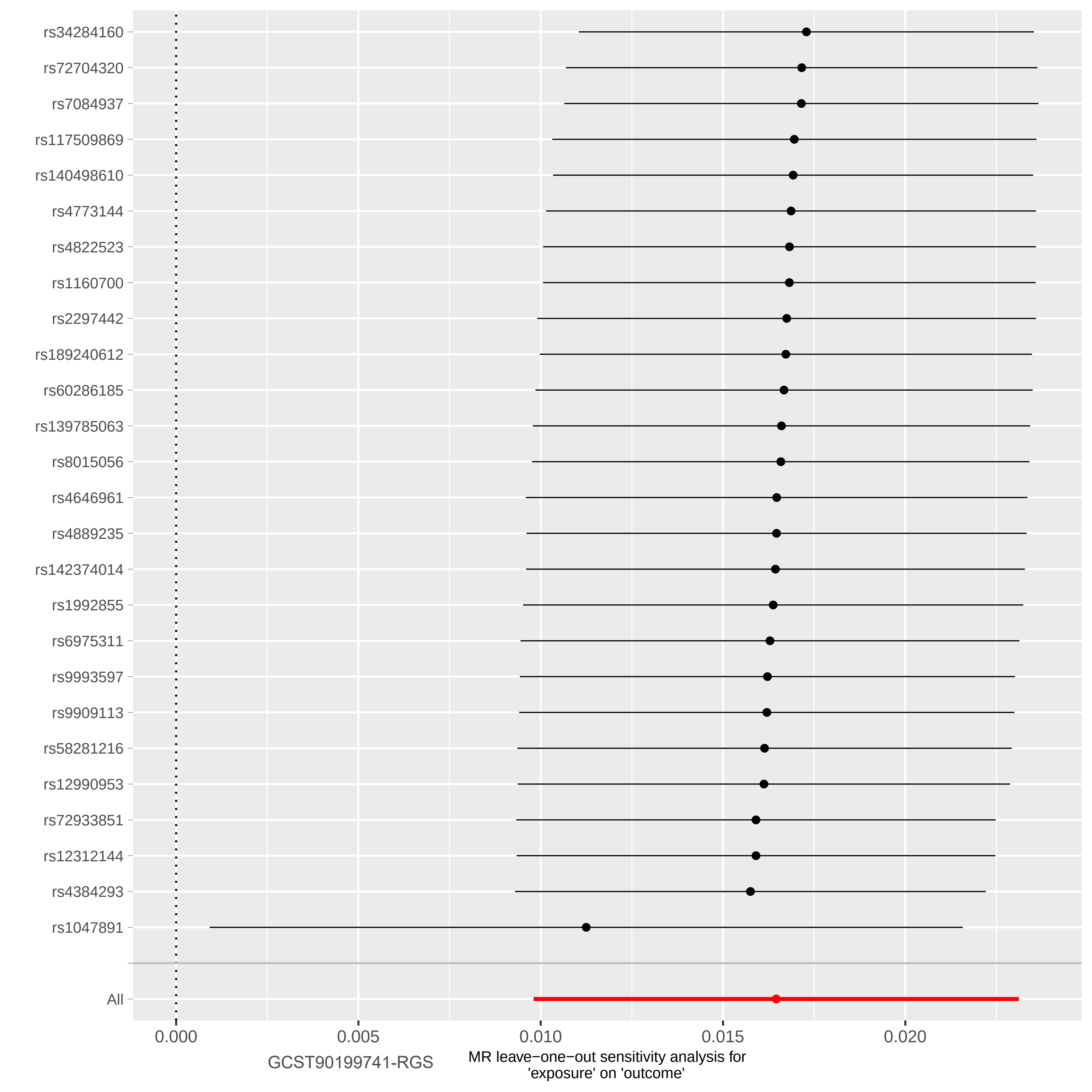

rs34284160
rs72704320
rs7084937
rs117509869
rs140498610
rs4773144
rs4822523
rs1160700
rs2297442
rs189240612
rs60286185
rs139785063
rs8015056
rs4646961
rs4889235
rs142374014
rs1992855
rs6975311
rs9993597
rs9909113
rs58281216
rs12990953
rs72933851
rs12312144
rs4384293
rs1047891
All
0.000
0.005
0.010
0.015
0.020
GCST90199741-RGS
MR leave−one−out sensitivity analysis for
'exposure' on 'outcome'

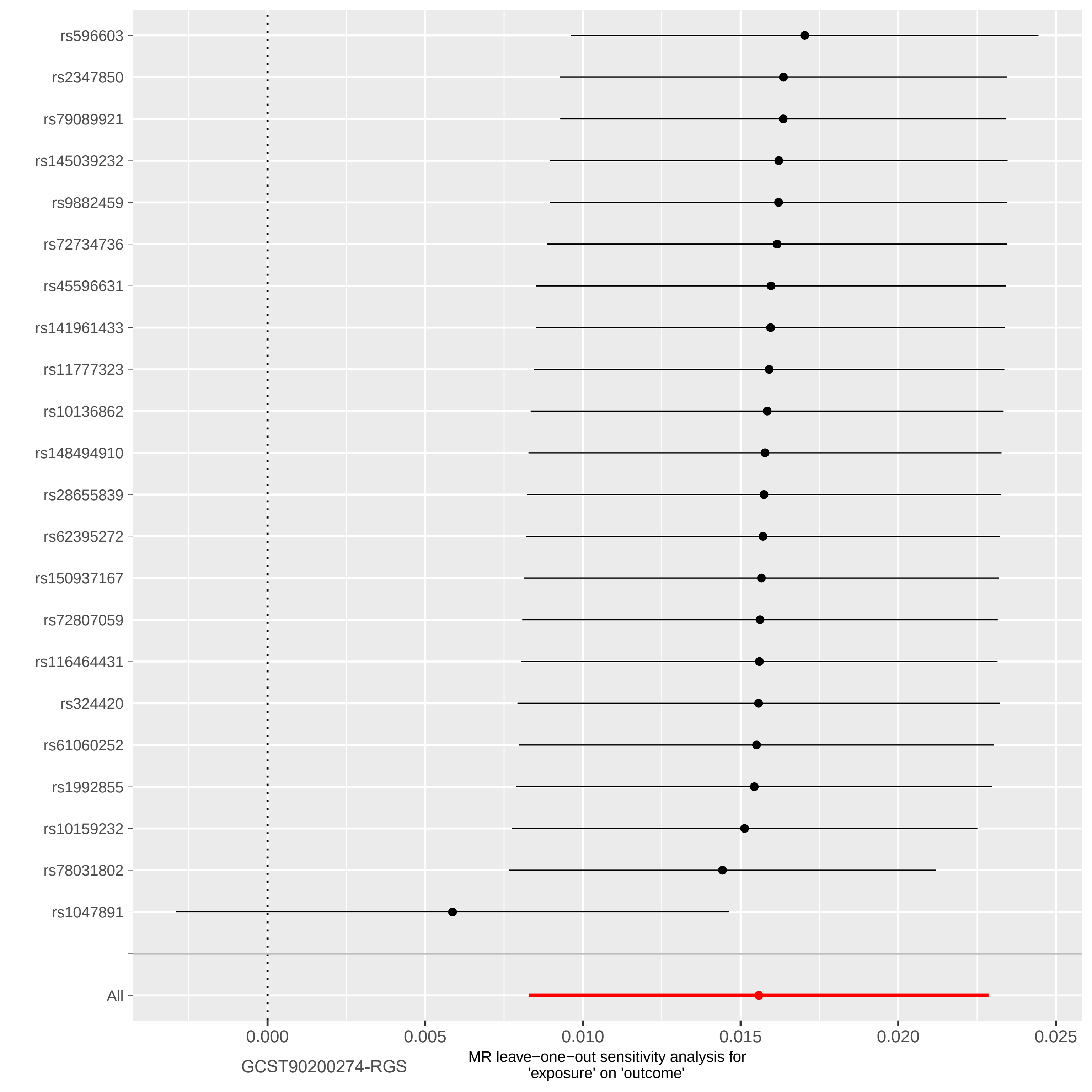
rs596603
rs2347850
rs79089921
rs145039232
rs9882459
rs72734736
rs45596631
rs141961433
rs11777323
rs10136862
rs148494910
rs28655839
rs62395272
rs150937167
rs72807059
rs116464431
rs324420
rs61060252
rs1992855
rs10159232
rs78031802
rs1047891
All
0.000
0.005
0.010
0.015
0.020
0.025
GCST90200274-RGS
MR leave−one−out sensitivity analysis for
'exposure' on 'outcome'

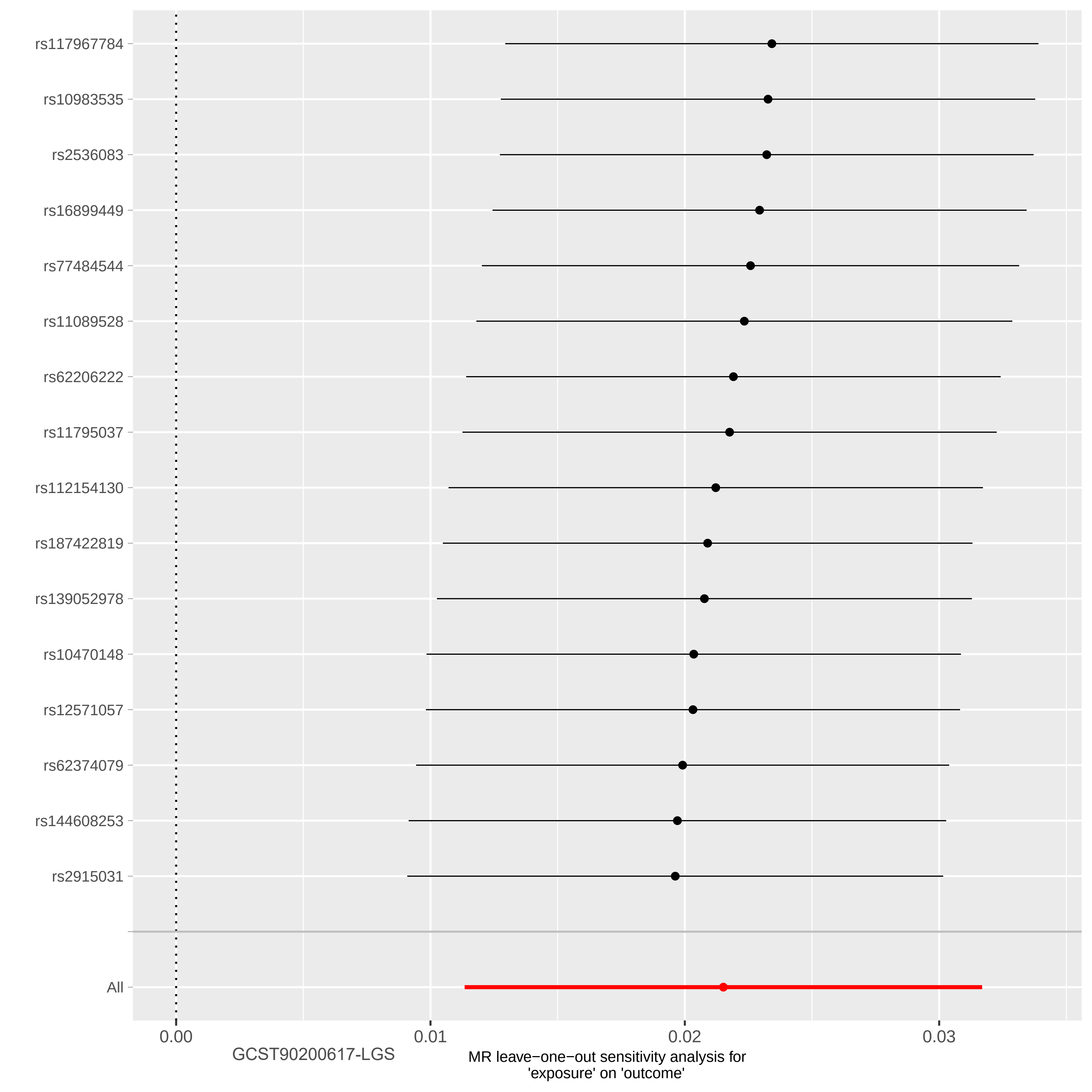

rs117967784
rs10983535
rs2536083
rs16899449
rs77484544
rs11089528
rs62206222
rs11795037
rs112154130
rs187422819
rs139052978
rs10470148
rs12571057
rs62374079
rs144608253
rs2915031
All
0.00
0.01
0.02
0.03
GCST90200617-LGS
MR leave−one−out sensitivity analysis for
'exposure' on 'outcome'

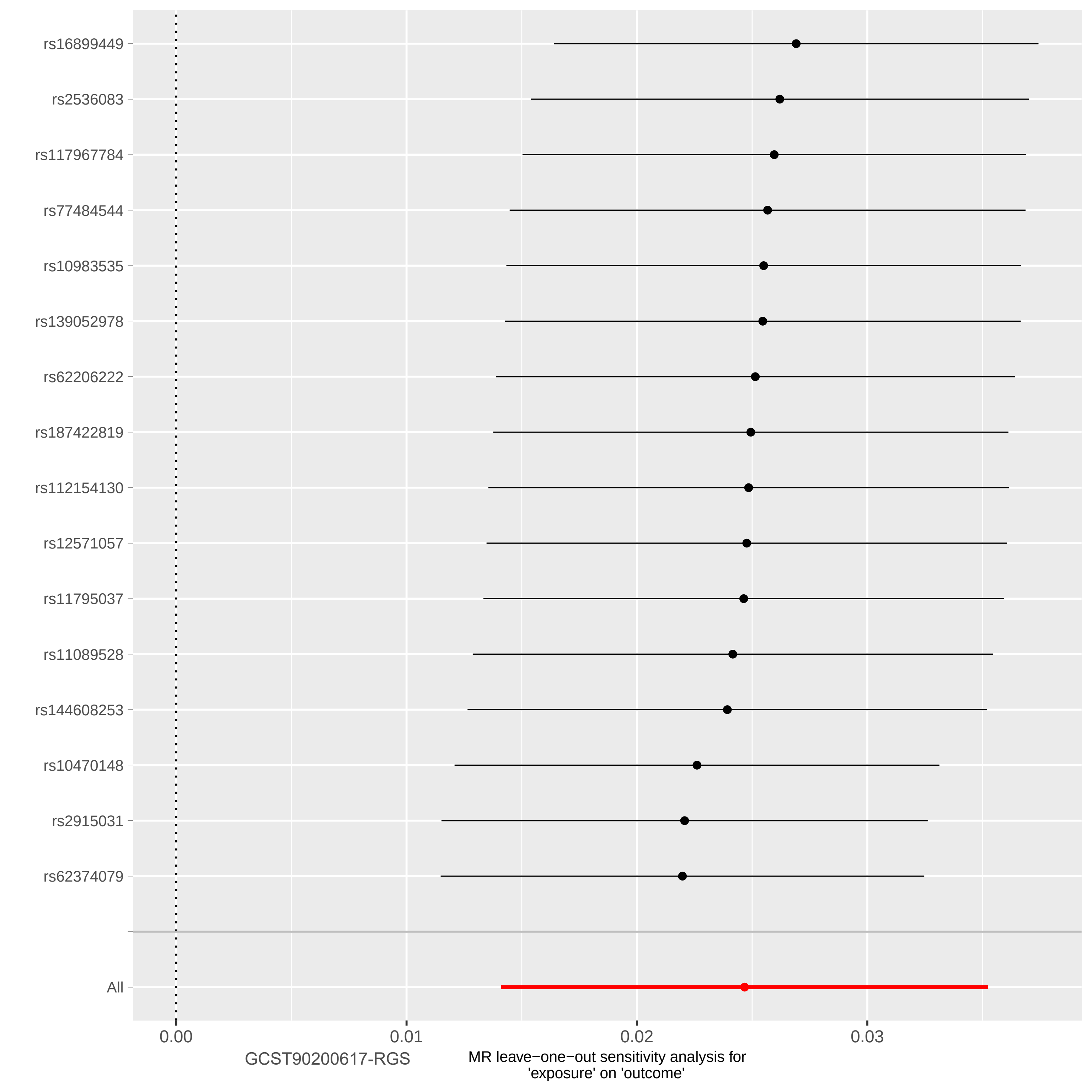
rs16899449
rs2536083
rs117967784
rs77484544
rs10983535
rs139052978
rs62206222
rs187422819
rs112154130
rs12571057
rs11795037
rs11089528
rs144608253
rs10470148
rs2915031
rs62374079
All
0.00
0.01
0.02
0.03
GCST90200617-RGS
MR leave−one−out sensitivity analysis for
'exposure' on 'outcome'

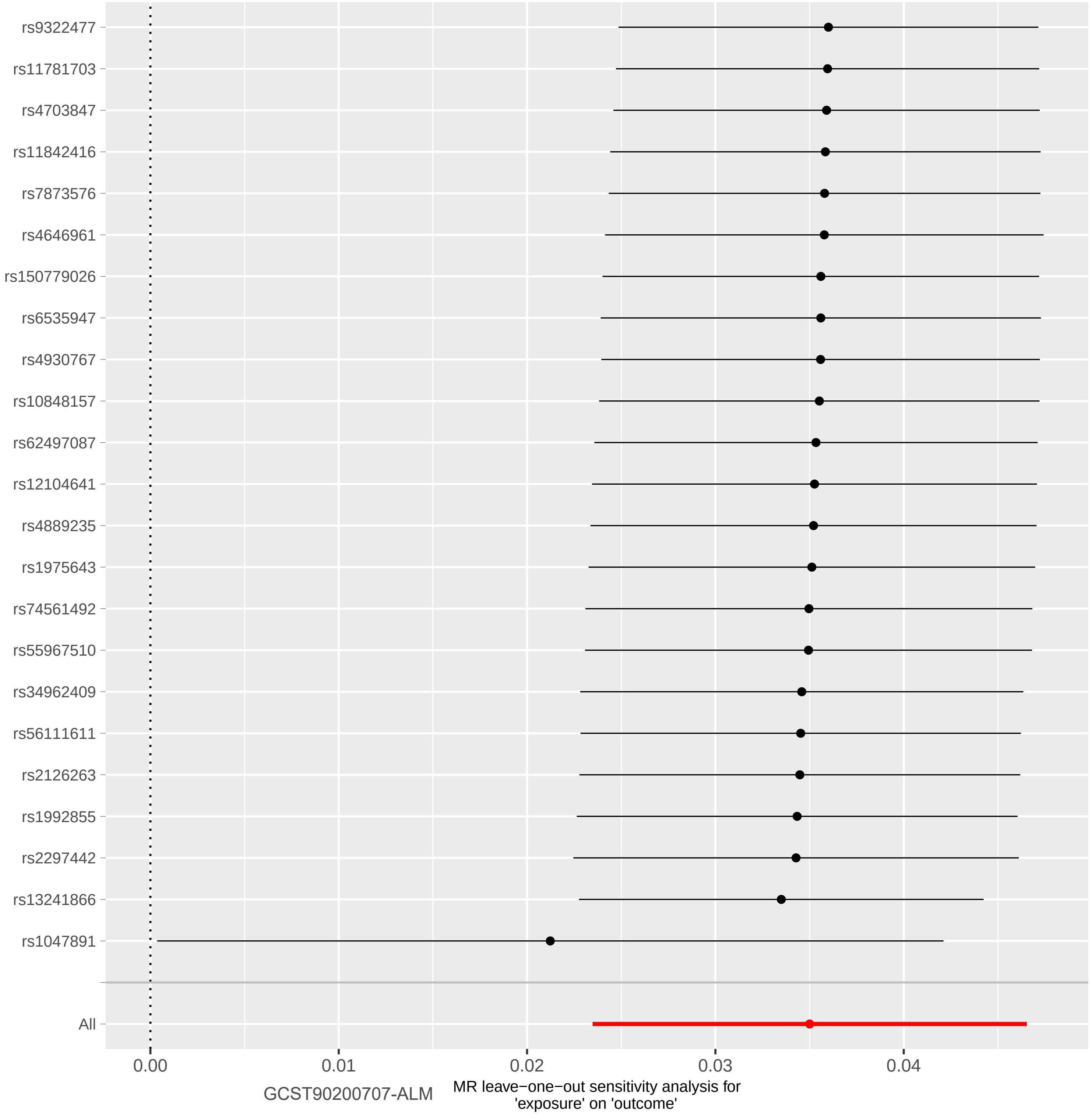
rs9322477
rs11781703
rs4703847
rs11842416
rs7873576
rs4646961
rs150779026
rs6535947
rs4930767
rs10848157
rs62497087
rs12104641
rs4889235
rs1975643
rs74561492
rs55967510
rs34962409
rs56111611
rs2126263
rs1992855
rs2297442
rs13241866
rs1047891
All
0.00
0.01
0.02
0.03
0.04
GCST90200707-ALM
MR leave−one−out sensitivity analysis for
'exposure' on 'outcome'

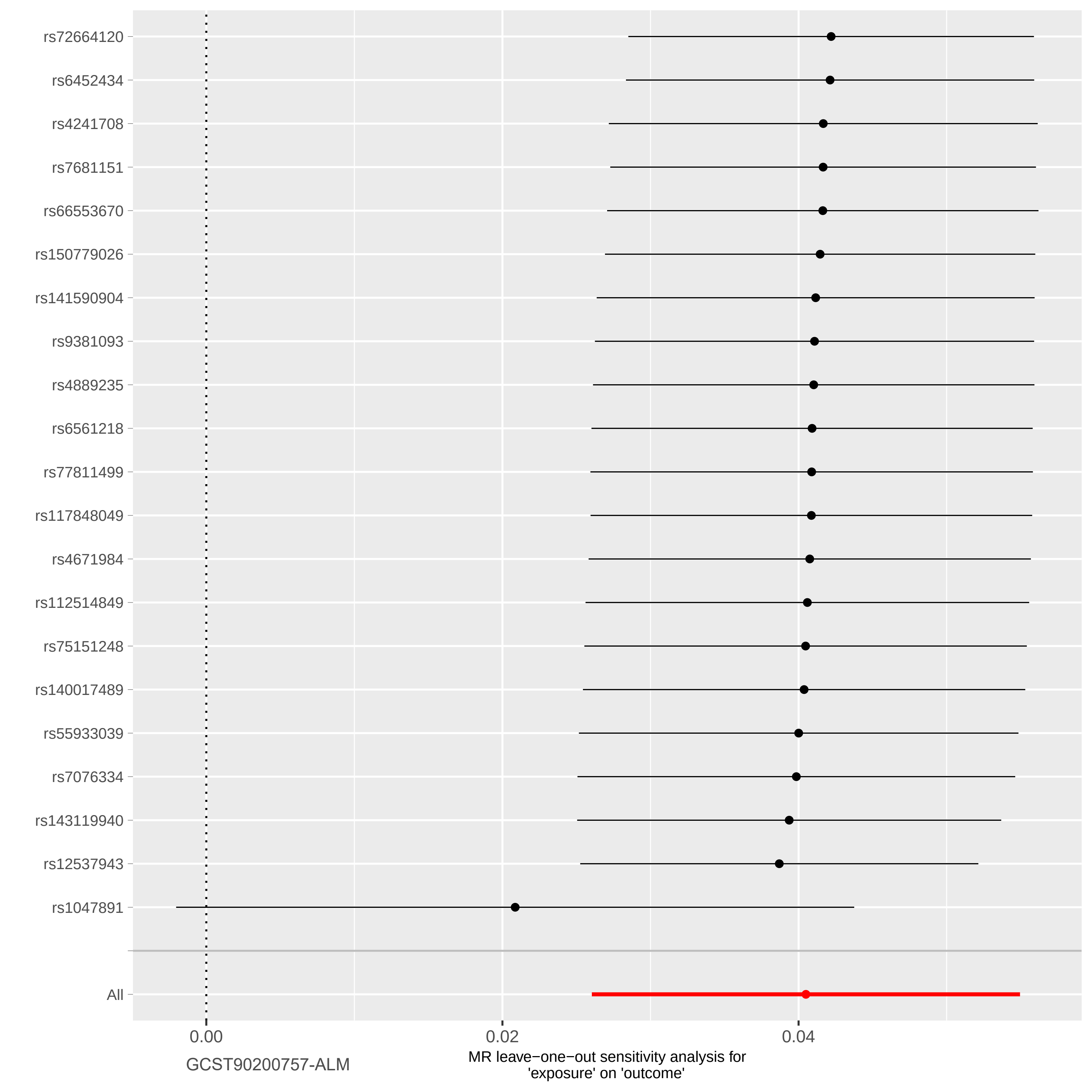

rs72664120
rs6452434
rs4241708
rs7681151
rs66553670
rs150779026
rs141590904
rs9381093
rs4889235
rs6561218
rs77811499
rs117848049
rs4671984
rs112514849
rs75151248
rs140017489
rs55933039
rs7076334
rs143119940
rs12537943
rs1047891
All
0.00
0.02
0.04
GCST90200757-ALM
MR leave−one−out sensitivity analysis for
'exposure' on 'outcome'

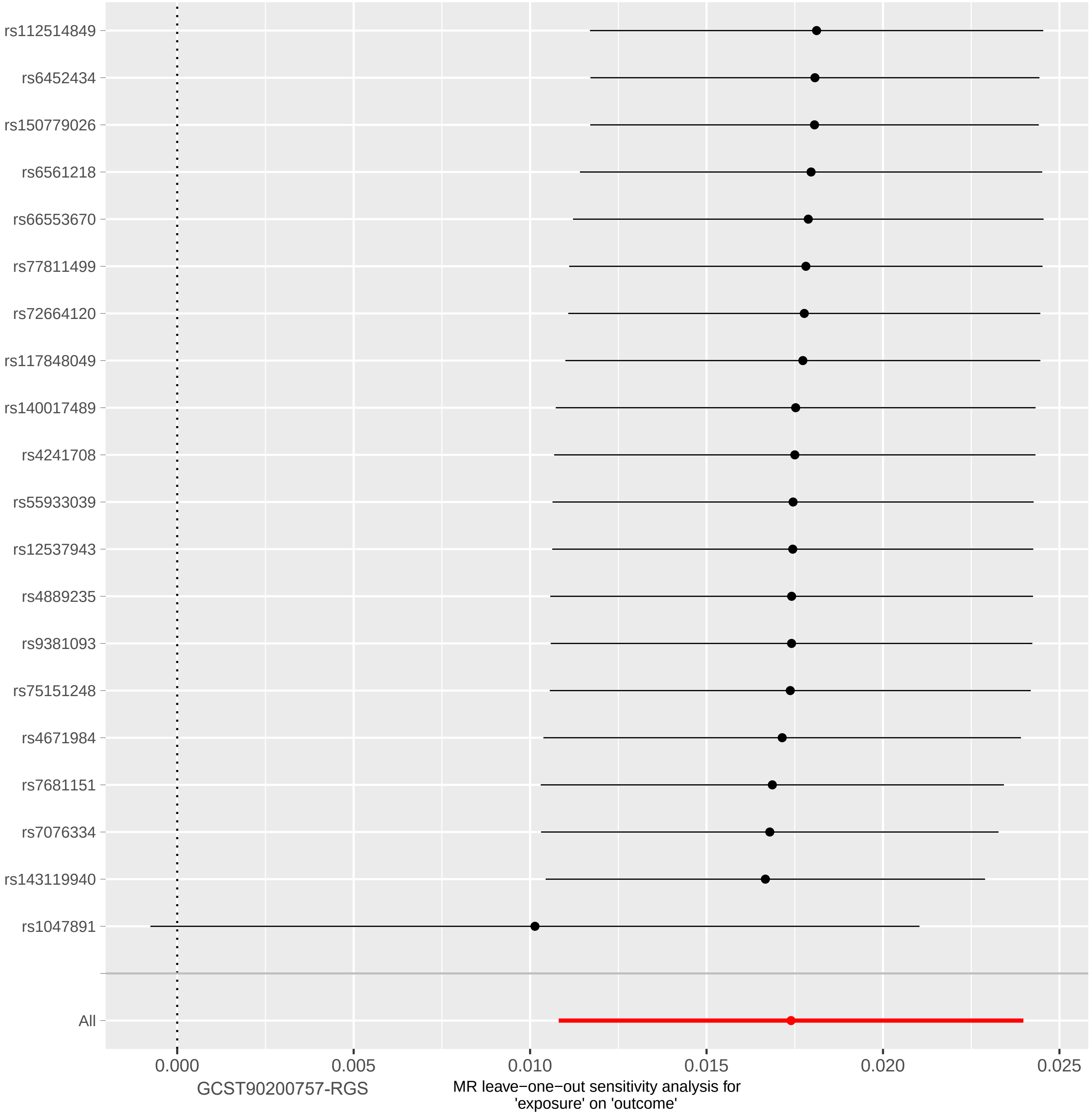
rs112514849
rs6452434
rs150779026
rs6561218
rs66553670
rs77811499
rs72664120
rs117848049
rs140017489
rs4241708
rs55933039
rs12537943
rs4889235
rs9381093
rs75151248
rs4671984
rs7681151
rs7076334
rs143119940
rs1047891
All
0.000
0.005
0.010
0.015
0.020
0.025
GCST90200757-RGS
MR leave−one−out sensitivity analysis for
'exposure' on 'outcome'

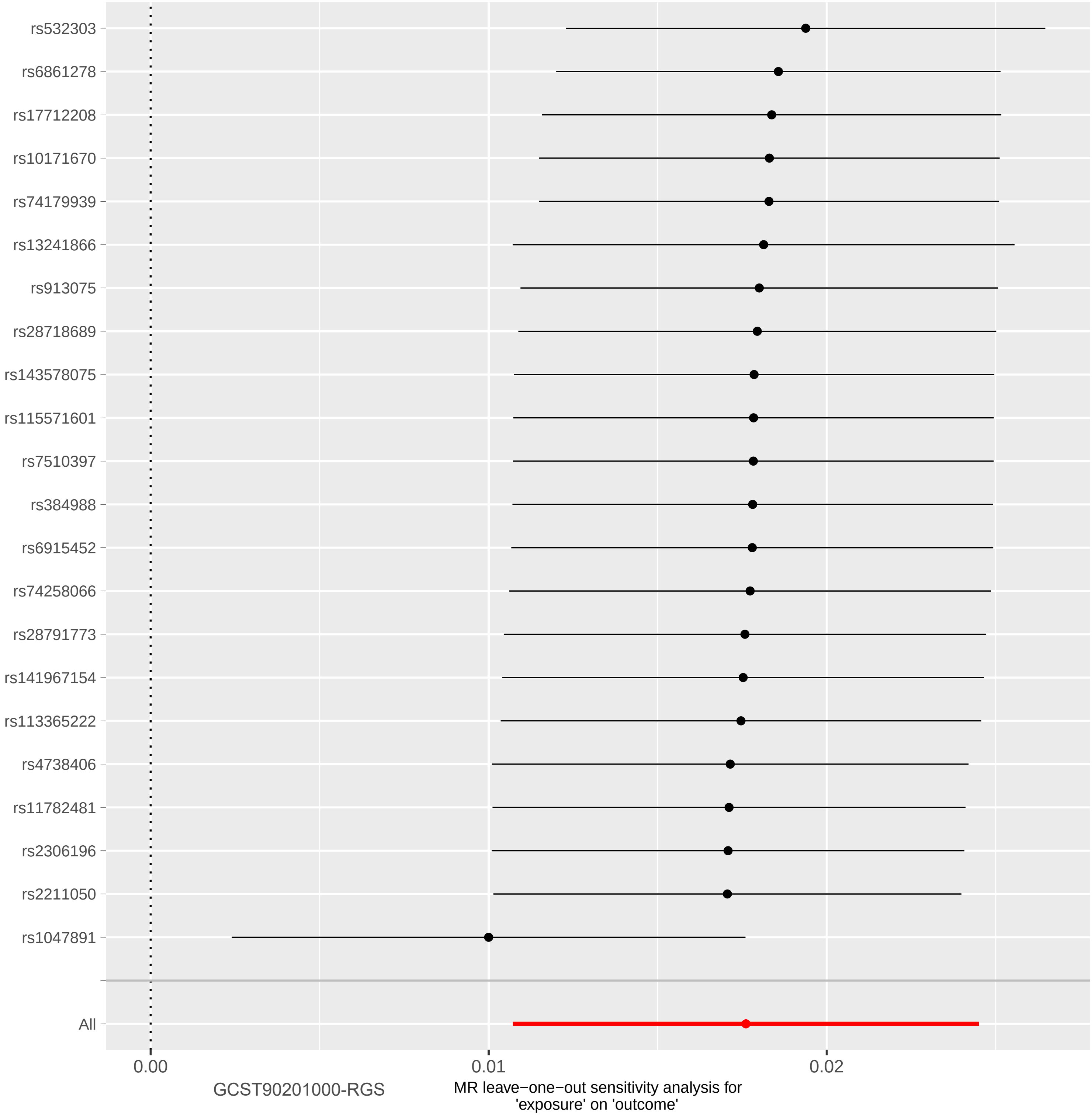

rs532303
rs6861278
rs17712208
rs10171670
rs74179939
rs13241866
rs913075
rs28718689
rs143578075
rs115571601
rs7510397
rs384988
rs6915452
rs74258066
rs28791773
rs141967154
rs113365222
rs4738406
rs11782481
rs2306196
rs2211050
rs1047891
All
0.00
0.01
0.02
GCST90201000-RGS
MR leave−one−out sensitivity analysis for
'exposure' on 'outcome'

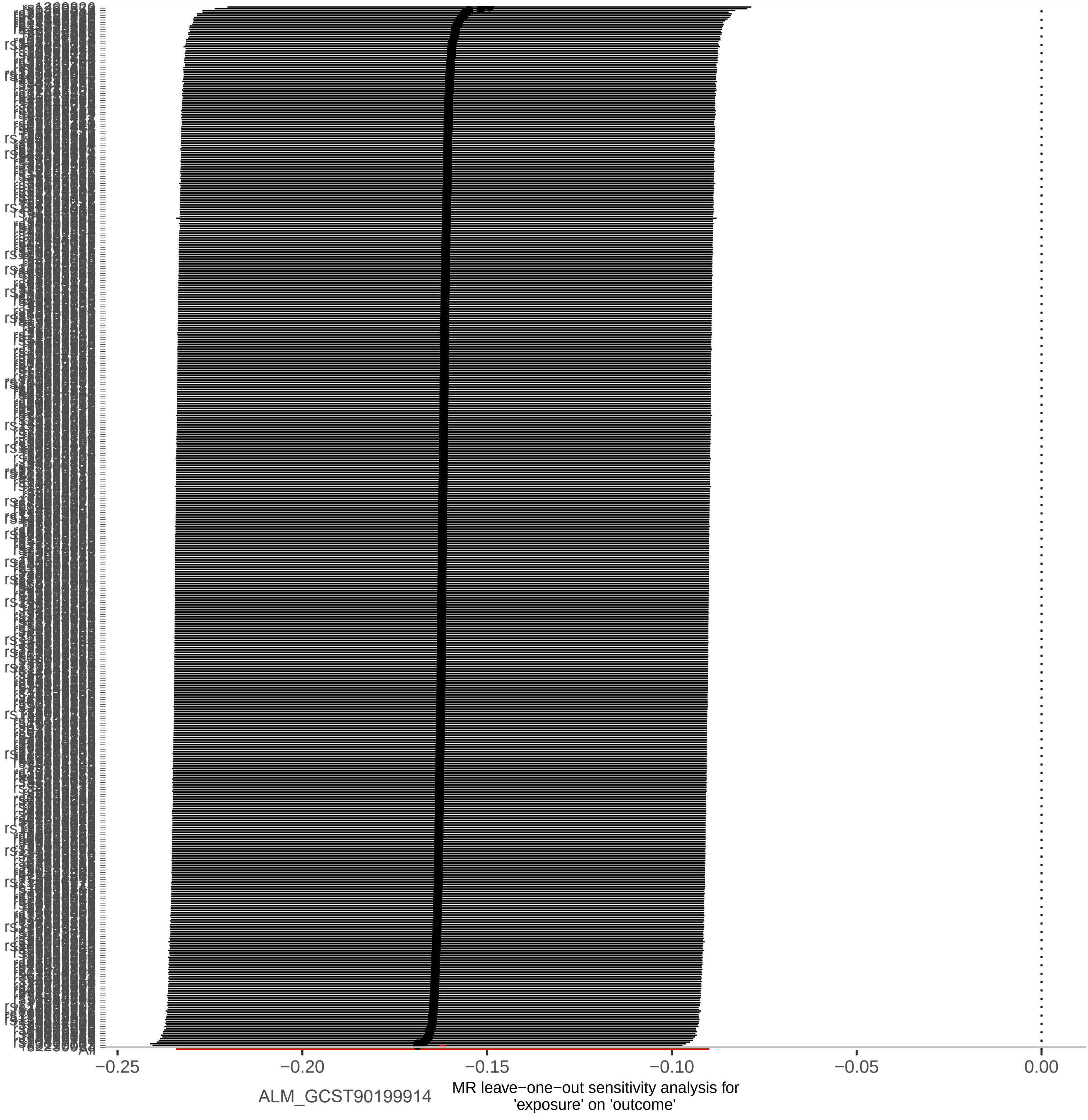

−0.25
−0.20
−0.15
−0.10
−0.05
0.00
ALM_GCST90199914
MR leave−one−out sensitivity analysis for 'exposure' on 'outcome'

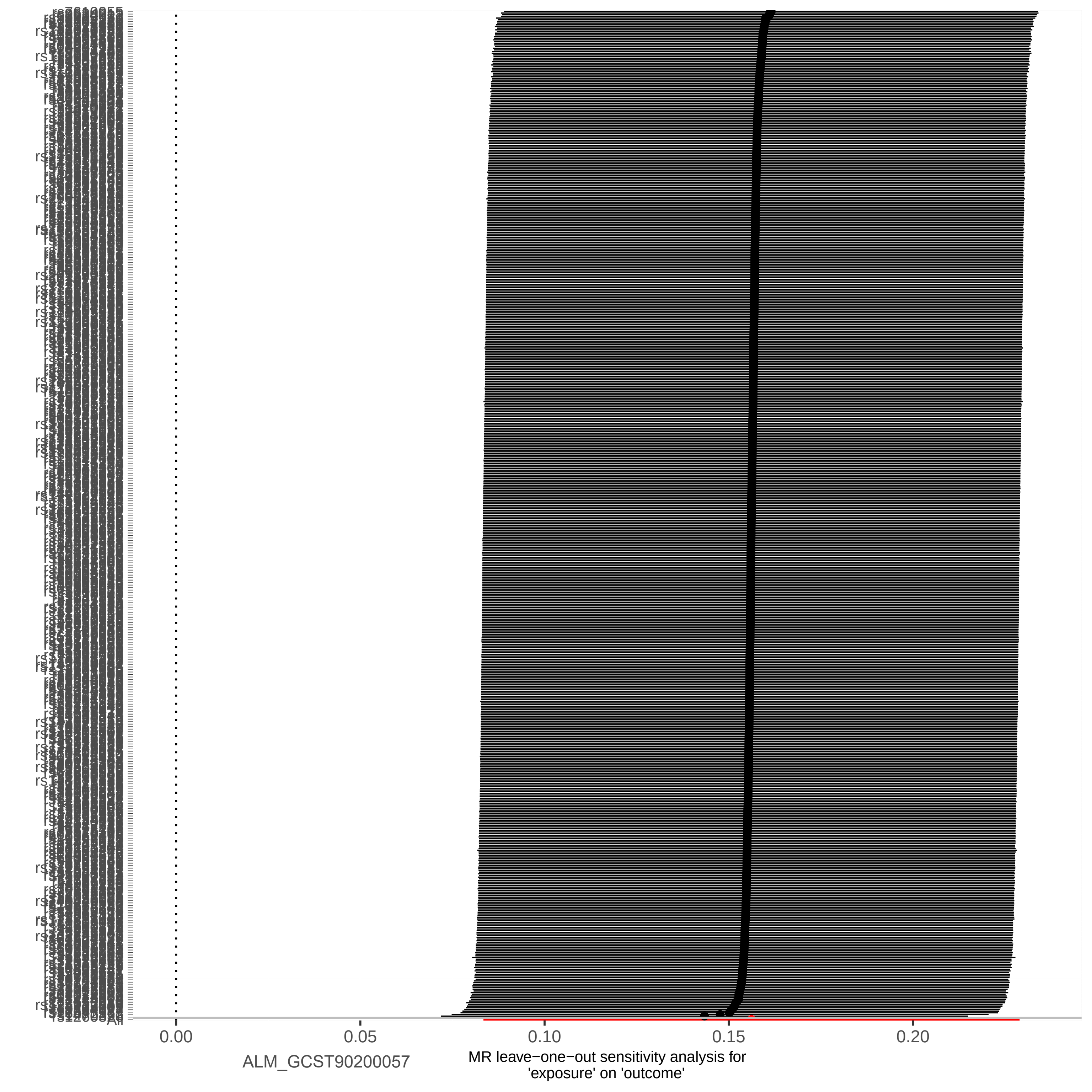

All
0.00
0.05
0.10
0.15
0.20
ALM_GCST90200057
MR leave−one−out sensitivity analysis for
'exposure' on 'outcome'

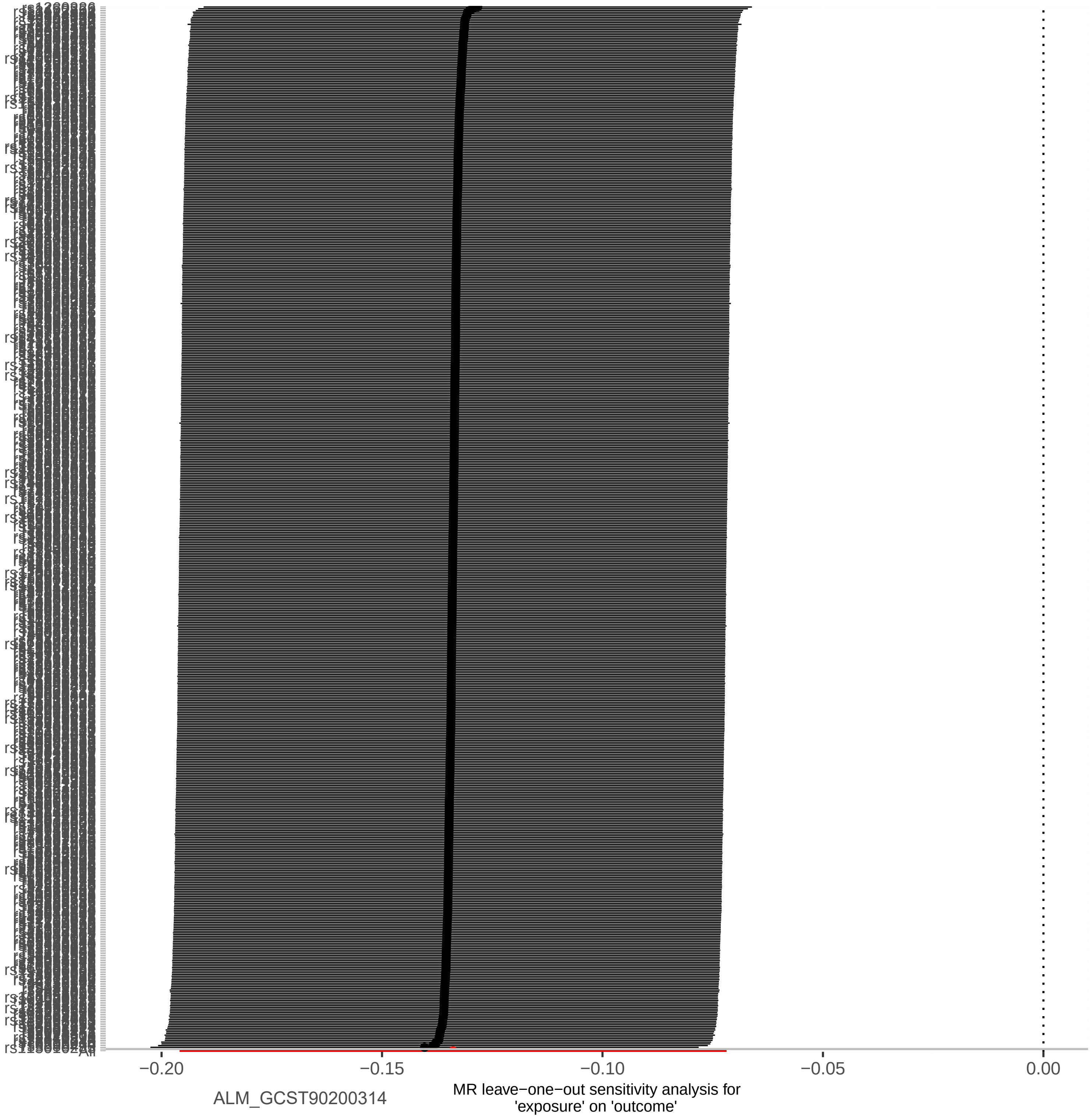

−0.20
−0.15
−0.10
−0.05
0.00
All
ALM_GCST90200314
MR leave−one−out sensitivity analysis for
'exposure' on 'outcome'

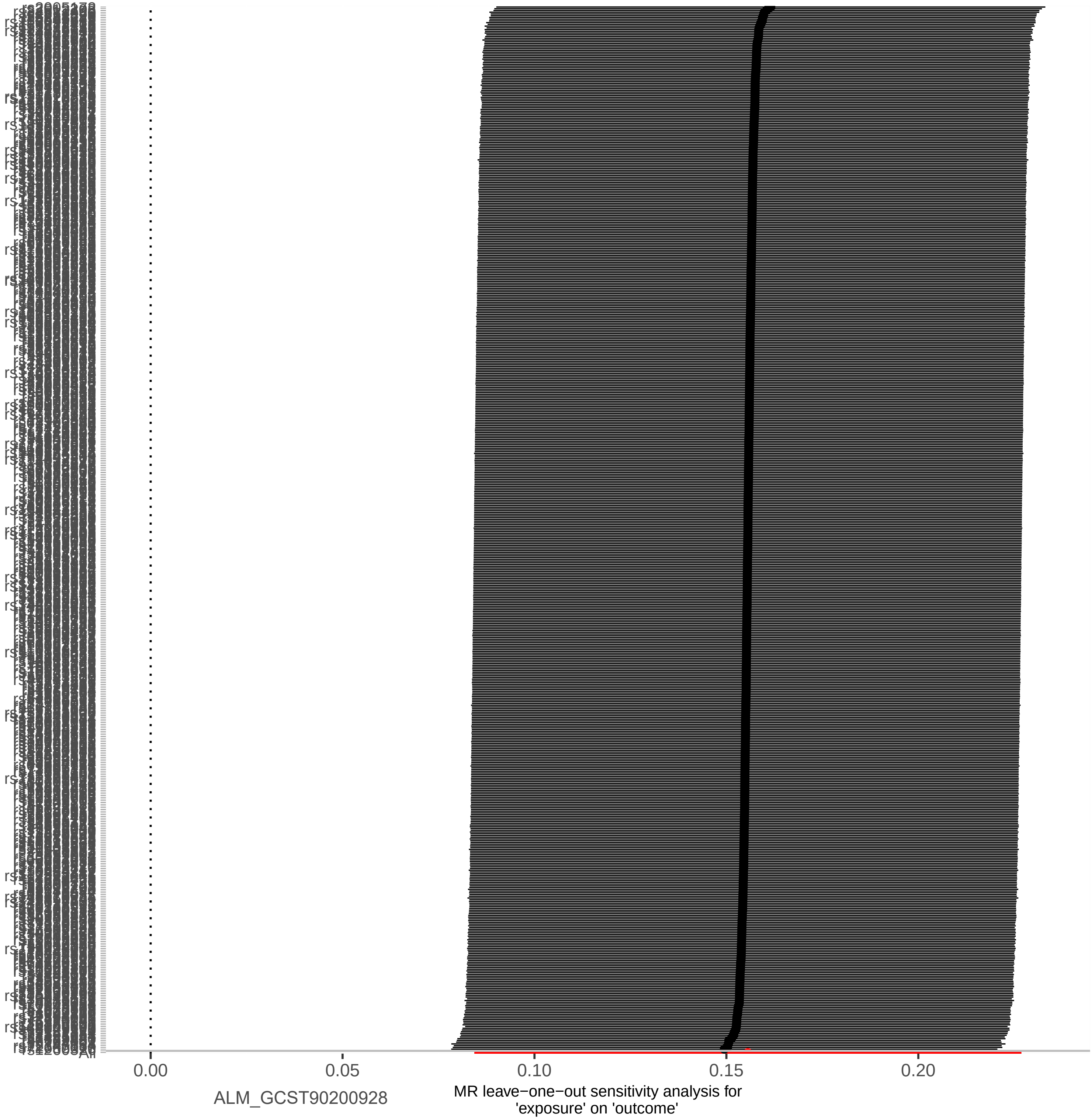
0.00
0.05
0.10
0.15
0.20
ALM_GCST90200928
MR leave−one−out sensitivity analysis for
'exposure' on 'outcome'

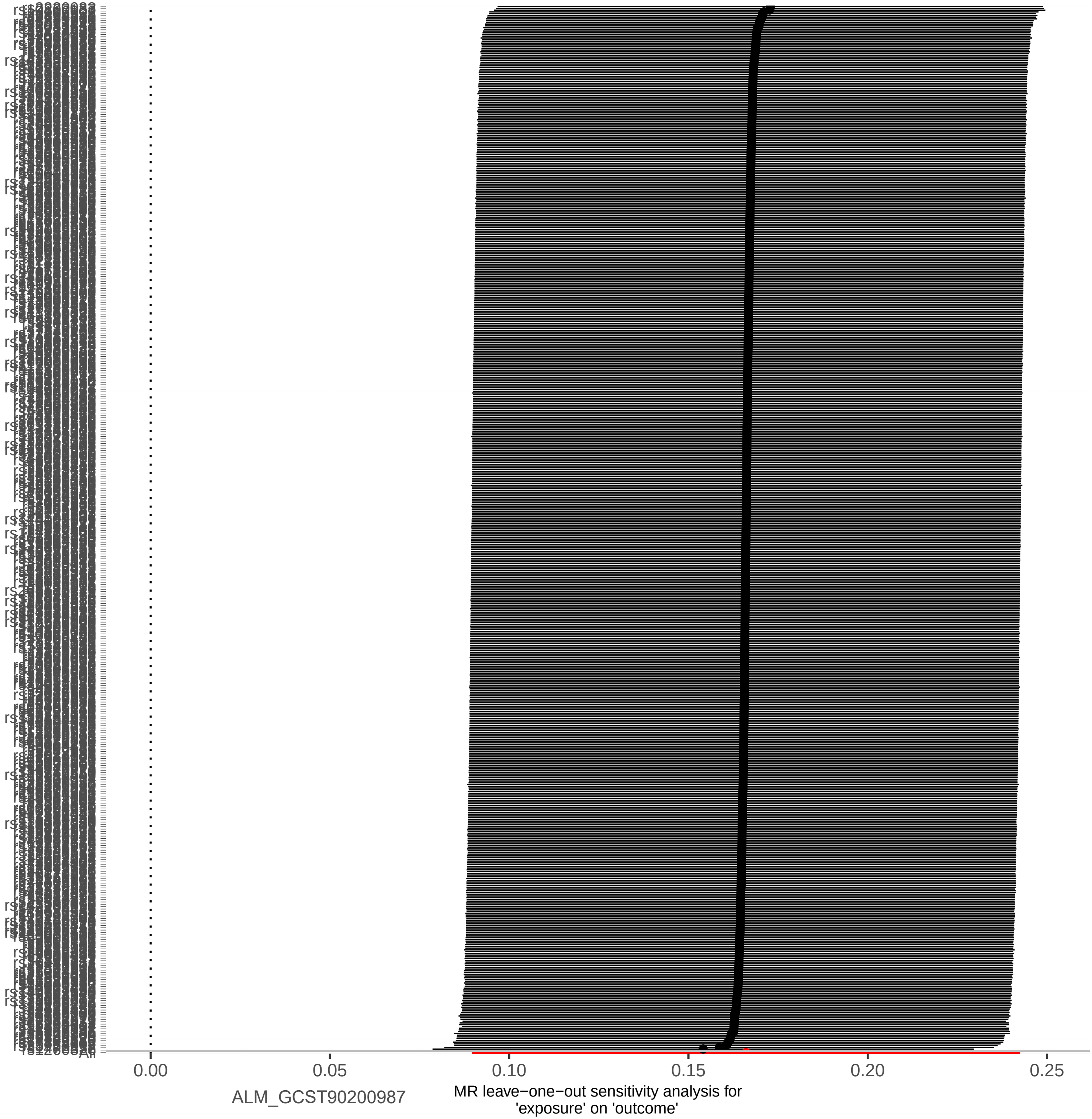
0.00
0.05
0.10
0.15
0.20
0.25
ALM_GCST90200987
MR leave−one−out sensitivity analysis for
'exposure' on 'outcome'

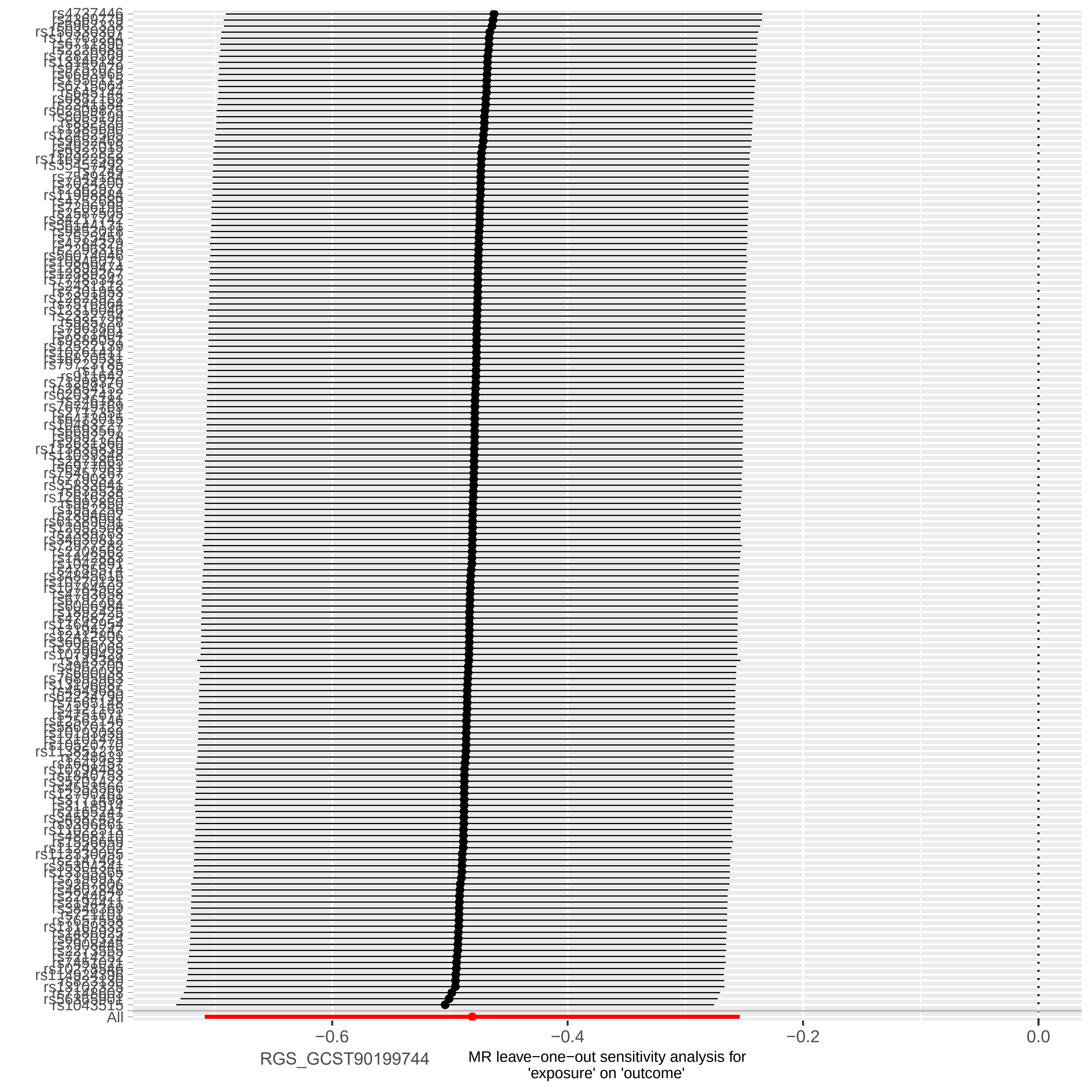

rs4737446
rs1043515
All
−0.6
−0.4
−0.2
0.0
RGS_GCST90199744
MR leave−one−out sensitivity analysis for
'exposure' on 'outcome'

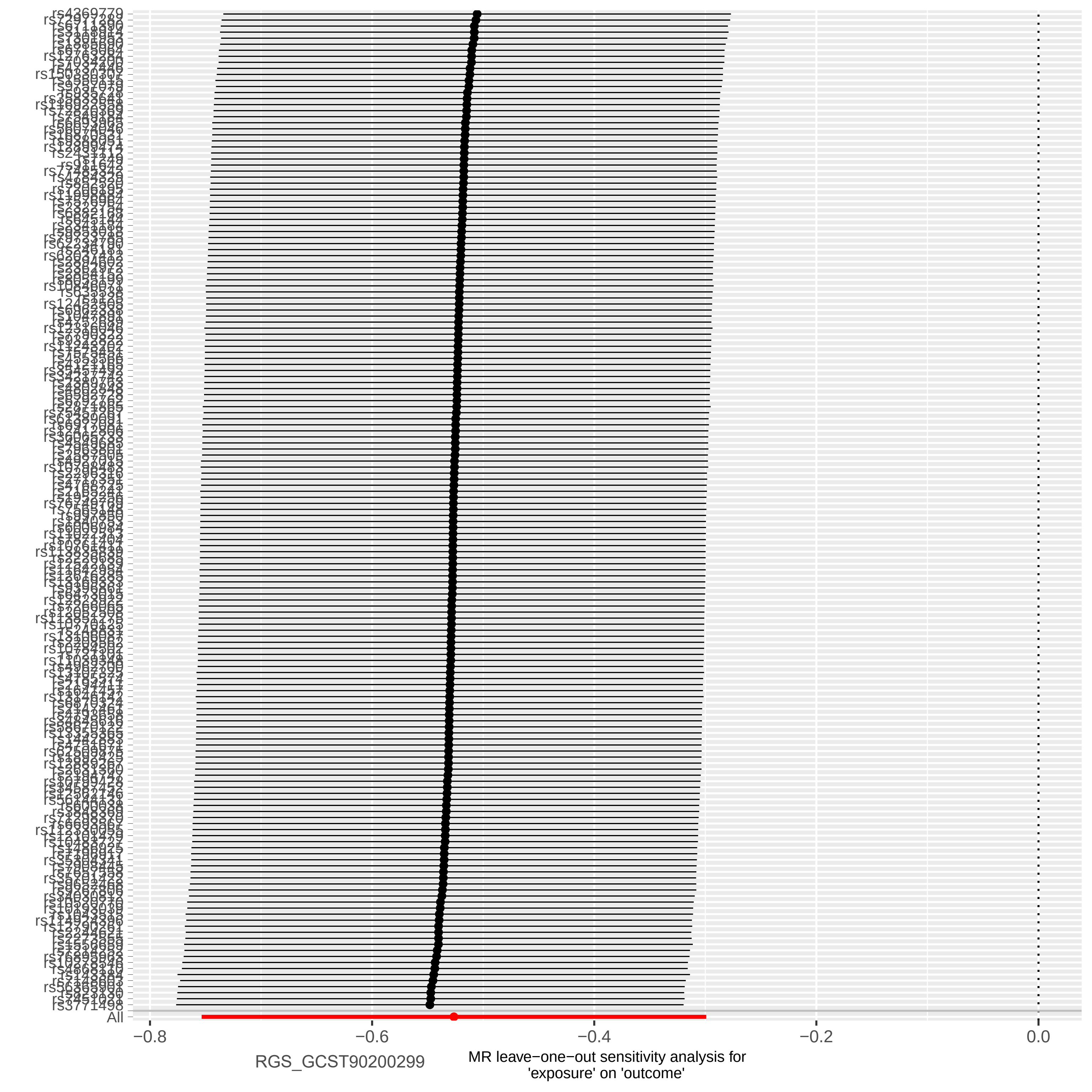
rs4269779
rs3771498
All
−0.8
−0.6
−0.4
−0.2
0.0
RGS_GCST90200299
MR leave−one−out sensitivity analysis for
'exposure' on 'outcome'

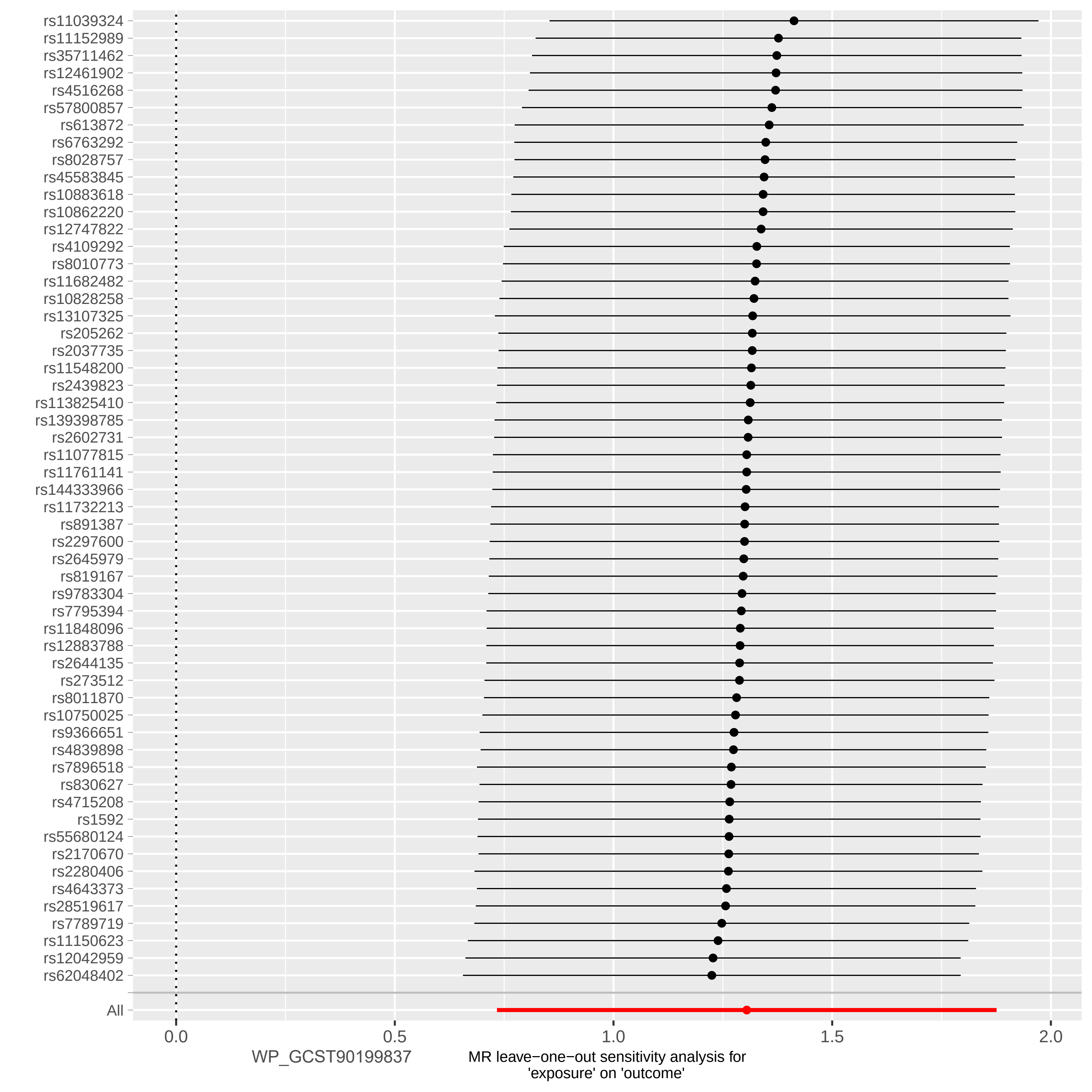

rs11039324
rs11152989
rs35711462
rs12461902
rs4516268
rs57800857
rs613872
rs6763292
rs8028757
rs45583845
rs10883618
rs10862220
rs12747822
rs4109292
rs8010773
rs11682482
rs10828258
rs13107325
rs205262
rs2037735
rs11548200
rs2439823
rs113825410
rs139398785
rs2602731
rs11077815
rs11761141
rs144333966
rs11732213
rs891387
rs2297600
rs2645979
rs819167
rs9783304
rs7795394
rs11848096
rs12883788
rs2644135
rs273512
rs8011870
rs10750025
rs9366651
rs4839898
rs7896518
rs830627
rs4715208
rs1592
rs55680124
rs2170670
rs2280406
rs4643373
rs28519617
rs7789719
rs11150623
rs12042959
rs62048402
All
0.0
0.5
1.0
1.5
2.0
WP_GCST90199837
MR leave−one−out sensitivity analysis for
'exposure' on 'outcome'

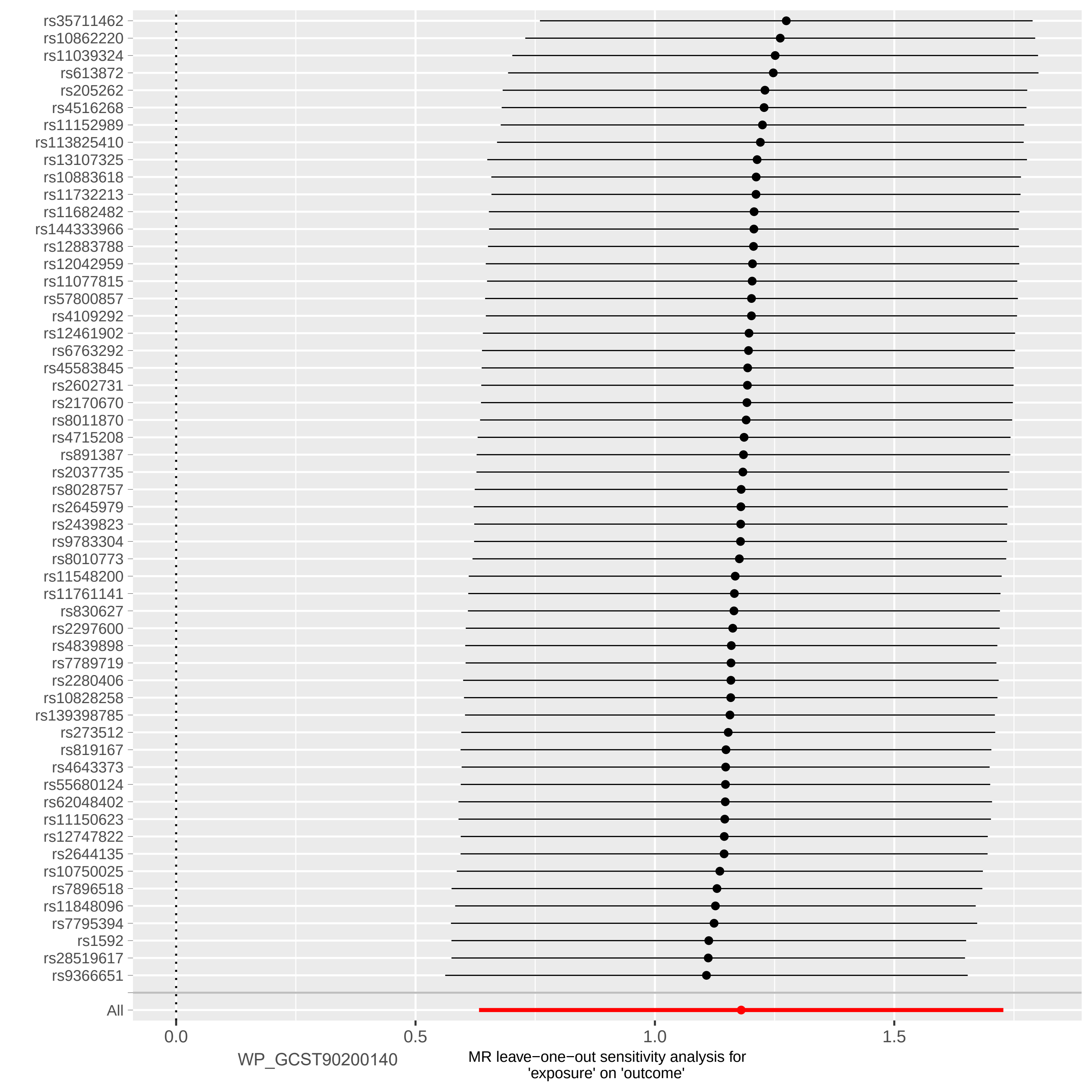
rs35711462
rs10862220
rs11039324
rs613872
rs205262
rs4516268
rs11152989
rs113825410
rs13107325
rs10883618
rs11732213
rs11682482
rs144333966
rs12883788
rs12042959
rs11077815
rs57800857
rs4109292
rs12461902
rs6763292
rs45583845
rs2602731
rs2170670
rs8011870
rs4715208
rs891387
rs2037735
rs8028757
rs2645979
rs2439823
rs9783304
rs8010773
rs11548200
rs11761141
rs830627
rs2297600
rs4839898
rs7789719
rs2280406
rs10828258
rs139398785
rs273512
rs819167
rs4643373
rs55680124
rs62048402
rs11150623
rs12747822
rs2644135
rs10750025
rs7896518
rs11848096
rs7795394
rs1592
rs28519617
rs9366651
All
0.0
0.5
1.0
1.5
WP_GCST90200140
MR leave−one−out sensitivity analysis for
'exposure' on 'outcome'

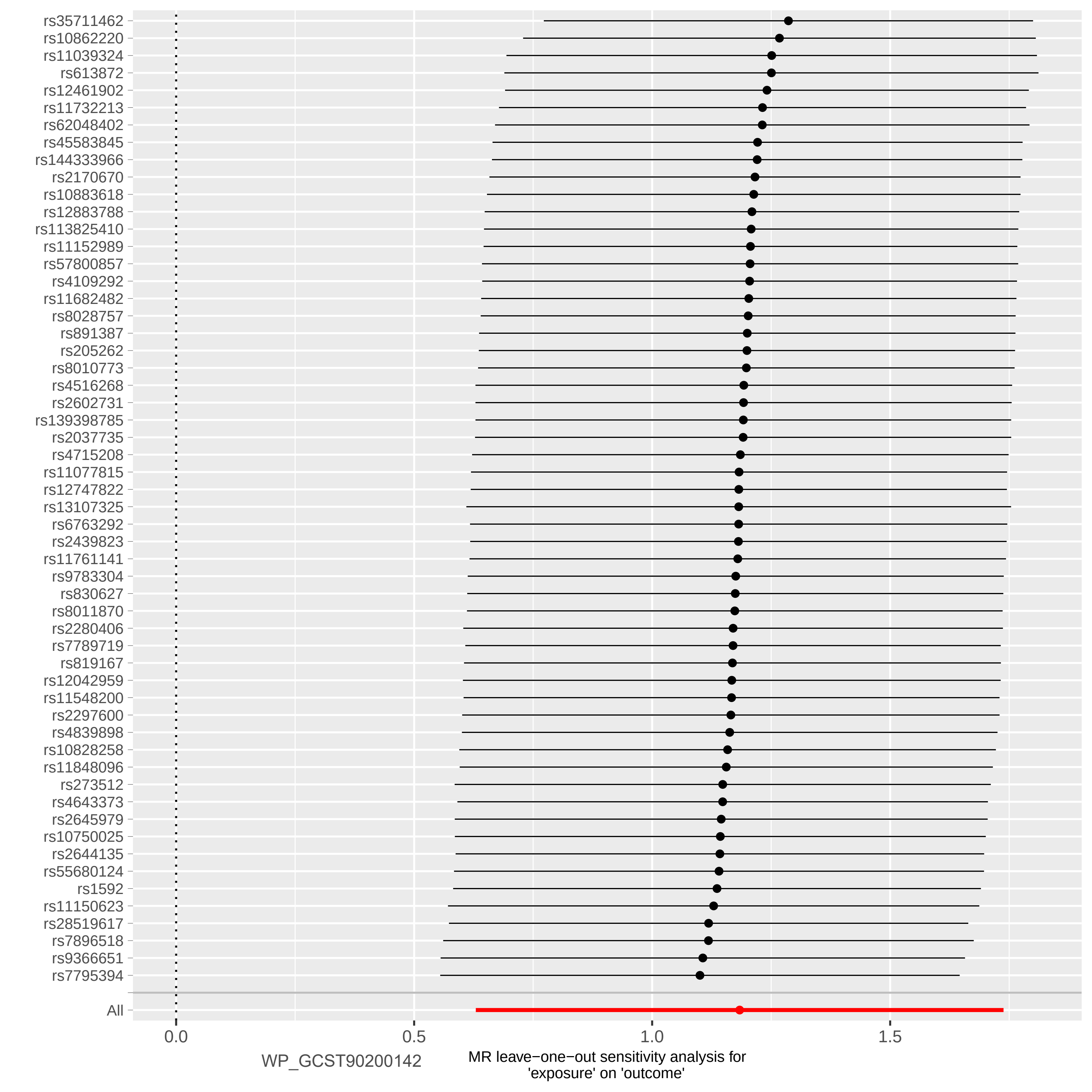
rs35711462
rs10862220
rs11039324
rs613872
rs12461902
rs11732213
rs62048402
rs45583845
rs144333966
rs2170670
rs10883618
rs12883788
rs113825410
rs11152989
rs57800857
rs4109292
rs11682482
rs8028757
rs891387
rs205262
rs8010773
rs4516268
rs2602731
rs139398785
rs2037735
rs4715208
rs11077815
rs12747822
rs13107325
rs6763292
rs2439823
rs11761141
rs9783304
rs830627
rs8011870
rs2280406
rs7789719
rs819167
rs12042959
rs11548200
rs2297600
rs4839898
rs10828258
rs11848096
rs273512
rs4643373
rs2645979
rs10750025
rs2644135
rs55680124
rs1592
rs11150623
rs28519617
rs7896518
rs9366651
rs7795394
All
0.0
0.5
1.0
1.5
WP_GCST90200142
MR leave−one−out sensitivity analysis for
'exposure' on 'outcome'

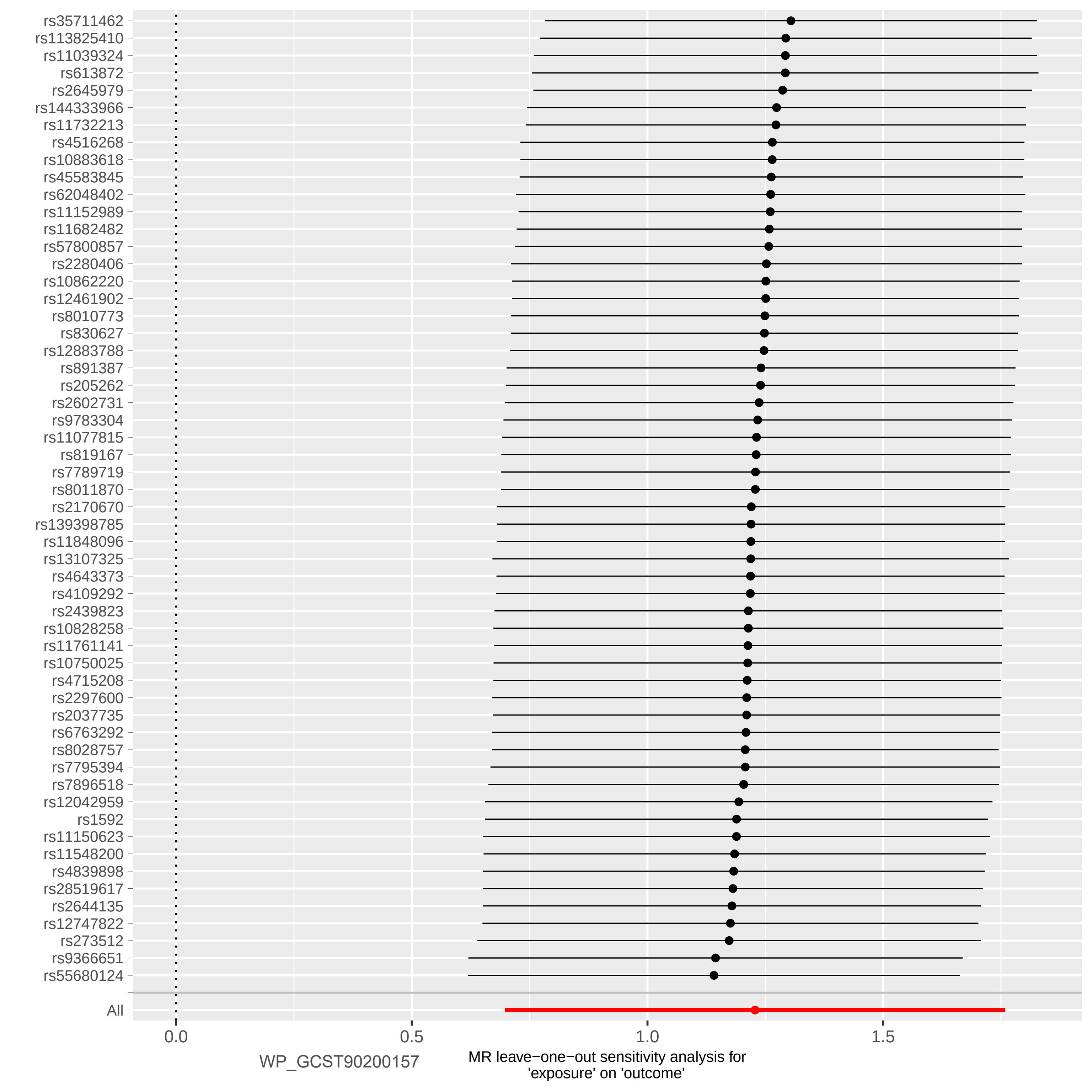
rs35711462
rs113825410
rs11039324
rs613872
rs2645979
rs144333966
rs11732213
rs4516268
rs10883618
rs45583845
rs62048402
rs11152989
rs11682482
rs57800857
rs2280406
rs10862220
rs12461902
rs8010773
rs830627
rs12883788
rs891387
rs205262
rs2602731
rs9783304
rs11077815
rs819167
rs7789719
rs8011870
rs2170670
rs139398785
rs11848096
rs13107325
rs4643373
rs4109292
rs2439823
rs10828258
rs11761141
rs10750025
rs4715208
rs2297600
rs2037735
rs6763292
rs8028757
rs7795394
rs7896518
rs12042959
rs1592
rs11150623
rs11548200
rs4839898
rs28519617
rs2644135
rs12747822
rs273512
rs9366651
rs55680124
All
0.0
0.5
1.0
1.5
WP_GCST90200157
MR leave−one−out sensitivity analysis for
'exposure' on 'outcome'
