# Supplementary material for: Causal Relationship Between Circulating Metabolites and Sarcopenia‐Related Traits: A Mendelian Randomization and Experimental Study
Source: Food Sci Nutr. 2025 Jan 9;13(1):e4624. doi: 10.1002/fsn3.4624 (PMC11717068; doi:10.1002/fsn3.4624)
Supplement: Supplementary file 6 — Table S4. MR sensitivity analyses of plasma metabolites and sarcopenia‐related traits as metabolites are the exposure. [file FSN3-13-e4624-s007.docx]

| **Supplementary Table 4. Sensitivity analysis of plasma metabolites and sarcopenia-related traits as metabolites is the exposure.** | | | | | | | | | | | | | | |
| --- | --- | --- | --- | --- | --- | --- | --- | --- | --- | --- | --- | --- | --- | --- |
| **Exposures** | **Outcomes** | **Heterogeneity test** | | | | | | | | | **Pleiotropy test** | | | |
|  |  | **IVW** | | | | | **MR-Egger** | | | **MR-Egger intercept** | | | | |
|  |  | Q | pval | | Q | | | pval | | | Intercept | | pval | |
| Gamma-glutamylglycine levels | ALM | 67.92 | | <0.01 | | 62.60 | | | <0.01 | | | -0.001728 | | 0.17 |
| Glycine levels | ALM | 88.06 | | <0.01 | | 76.48 | | | <0.01 | | | -0.002322 | | 0.09 |
| Glycine to alanine ratio | ALM | 87.32 | | <0.01 | | 83.25 | | | <0.01 | | | -0.002063 | | 0.35 |
| Propionylglycine levels | ALM | 92.94 | | <0.01 | | 77.85 | | | <0.01 | | | -0.004708 | | 0.06 |
| Propionylglycine levels | LGS | 14.49 | | 0.49 | | 13.80 | | | 0.47 | | | -0.000847 | | 0.42 |
| X-23665 levels | LGS | 13.97 | | 0.53 | | 13.68 | | | 0.47 | | | -0.000881 | | 0.60 |
| Glycine to alanine ratio | RGS | 26.78 | | 0.11 | | 26.20 | | | 0.10 | | | -0.000613 | | 0.54 |
| Serine to threonine ratio | RGS | 27.94 | | 0.14 | | 25.26 | | | 0.19 | | | -0.001346 | | 0.16 |
| Gamma-glutamylglycine levels | RGS | 42.95 | | 0.10 | | 44.55 | | | <0.01 | | | -0.000757 | | 0.35 |
| X-23665 levels | RGS | 16.16 | | 0.37 | | 16.02 | | | 0.31 | | | 0.000596 | | 0.73 |
