# Supplementary material for: Causal Relationship Between Circulating Metabolites and Sarcopenia‐Related Traits: A Mendelian Randomization and Experimental Study
Source: Food Sci Nutr. 2025 Jan 9;13(1):e4624. doi: 10.1002/fsn3.4624 (PMC11717068; doi:10.1002/fsn3.4624)
Supplement: Supplementary file 7 — Table S5. MR sensitivity analyses of plasma metabolites and sarcopenia‐related traits as metabolites are the outcome. [file FSN3-13-e4624-s006.docx]

| **Supplementary Table 5. Sensitivity analysis of plasma metabolites and sarcopenia-related traits** **as metabolites is the outcome.** | | | | | | | | | | | | | | | |  |
| --- | --- | --- | --- | --- | --- | --- | --- | --- | --- | --- | --- | --- | --- | --- | --- | --- |
| **Exposures** | **Outcomes** | **Heterogeneity test** | | | | | | | | | **Pleiotropy test** | | | | |  |
|  |  | **IVW** | | | | | **MR-Egger** | | | **MR-Egger intercept** | | | | | |  |
|  |  | Q | pval | | Q | | | pval | | | | | Intercept | pval | | |
| ALM | Oleoyl-linoleoyl-glycerol (18:1/18:2) [2] levels | 699.86 | | <0.01 | | 695.80 | | | <0.01 | | | 0.003530 | | | 0.06 |  |
| ALM | N-palmitoyl-sphingosine to N-stearoyl-sphingosine ratio | 769.40 | | <0.01 | | 769.31 | | | <0.01 | | | -0.000526 | | | 0.78 |  |
| ALM | Benzoate to oleoyl-linoleoyl-glycerol (18:1 to 18:2) [2] ratio | 587.76 | | 0.65 | | 585.33 | | | 0.65 | | | 0.001076 | | | 0.51 |  |
| ALM | N-acetylneuraminate levels | 686.14 | | <0.01 | | 686.02 | | | <0.01 | | | 0.000614 | | | 0.74 |  |
| ALM | 1-(1-enyl-palmitoyl)-2-oleoyl-gpc (p-16:0/18:1) levels | 14.49 | | 0.49 | | 13.80 | | | 0.47 | | | -0.002051 | | | 0.31 |  |
| RGS | Oleoyl ethanolamide levels | 137.99 | | 0.93 | | 137.99 | | | 0.92 | | | 0.000023 | | | 1.00 |  |
| RGS | Eicosenoate (20:1) levels | 160.05 | | 0.57 | | 159.52 | | | 0.56 | | | -0.003614 | | | 0.47 |  |
| WP | Carotene diol (3) levels | 72.64 | | 0.06 | | 72.45 | | | <0.05 | | | 0.004164 | | | 0.71 |  |
| WP | Carotenoid levels (cryptoxanthin) | 66.26 | | 0.14 | | 66.25 | | | 0.12 | | | 0.001177 | | | 0.91 |  |
| WP | Carotene diol (2) levels | 68.48 | | 0.10 | | 68.48 | | | 0.09 | | | 0.000758 | | | 0.94 |  |
| WP | Carotene diol (1) levels | 57.79 | | 0.37 | | 57.57 | | | 0.34 | | | -0.004657 | | | 0.65 |  |
