# Supplementary material for: Causal Relationship Between Circulating Metabolites and Sarcopenia‐Related Traits: A Mendelian Randomization and Experimental Study
Source: Food Sci Nutr. 2025 Jan 9;13(1):e4624. doi: 10.1002/fsn3.4624 (PMC11717068; doi:10.1002/fsn3.4624)
Supplement: Supplementary file 8 — Table S6. Sensitivity analysis of the glycine level and sarcopenia‐related traits in validation. [file FSN3-13-e4624-s004.docx]

| **Supplementary Table 7. Sensitivity analysis of glycine level and sarcopenia-related traits in validation.** | | | | | | | | | | | | | | |
| --- | --- | --- | --- | --- | --- | --- | --- | --- | --- | --- | --- | --- | --- | --- |
| **Exposures** | **Outcomes** | **Heterogeneity test** | | | | | | | | | **Pleiotropy test** | | | |
|  |  | **IVW** | | | | | **MR-Egger** | | | **MR-Egger intercept** | | | | |
|  |  | Q | pval | | Q | | | pval | | | Intercept | | pval | |
| Glycine levels (GCST90301967) | ALM | 1260.90 | | <0.01 | | 1234.98 | | | <0.01 | | | 0.001404 | | 0.20 |
| Glycine levels (GCST90301967) | LGS | 168.94 | | <0.01 | | 167.69 | | | <0.01 | | | 0.000411 | | 0.45 |
| Glycine levels (GCST90301967) | RGS | 170.86 | | <0.01 | | 169.21 | | | <0.01 | | | 0.000413 | | 0.38 |
